# π‑Conjugation Mediates Competing *J*_12_ and *J*_13_ Interactions at the Nanometric Scale in Linear Fe(III) Porphyrin Trimers

**DOI:** 10.1021/acs.inorgchem.6c03219

**Published:** 2026-08-05

**Authors:** Iago Pozo, Lidao Li, Ramón Torres-Cavanillas, Wojciech Stawski, William K. Myers, Michael Slota, Igor Rončević, Lapo Bogani, Harry L. Anderson

**Affiliations:** † Department of Chemistry, Chemistry Research Laboratory, 6396 Oxford University, Oxford OX1 3TA, United Kingdom; ‡ Centro Singular de Investigación en Química Biolóxica e Materiais Moleculares (CIQUS) and Departamento de Química Orgánica, Universidade de Santiago de Compostela, Santiago de Compostela, 15782, Spain; § Department of Materials, 9300 Oxford University, Oxford OX1 3PH, United Kingdom; ∥ Department of Chemistry and Department of Physics, University of Florence, Florence 50019, Italy; ⊥ Centre for Advanced ESR, Department of Chemistry, Inorganic Chemistry Laboratory, University of Oxford, South Parks Road, Oxford OX1 3QR, United Kingdom; # Department of Chemistry, The University of Manchester, Manchester M13 9PL, United Kingdom

## Abstract

The control of spin–spin interactions is a central challenge in designing materials for quantum technologies. A promising avenue for increasing magnetic coupling between spin centers is to connect them via a delocalized π-conjugated system. Here, we synthesized a family of porphyrins (monomers, dimers, and trimers) with high-spin Fe^III^ centers and studied their magnetic interactions. The linearly fused Fe­(III)Cl porphyrin trimers exhibit nearest-neighbor and second-neighbor couplings of the same sign and order of magnitude (*J*_12_ = −24.3 GHz; *J*_13_ = −10.8 GHz) between spin centers at 0.84 and 1.7 nm separation, respectively. These antiferromagnetic interactions lead to frustration, realizing a nontrivial Heisenberg spin system. The persistence of the long-range exchange coupling (*J*_13_) demonstrates that π-mediation is effective despite the spin polarization of the π-system induced by the central iron center. This work establishes a design principle for constructing nontrivial antiferromagnetic Heisenberg spin chains in which metal spin centers interact over several nanometers.

## Graphical abstract

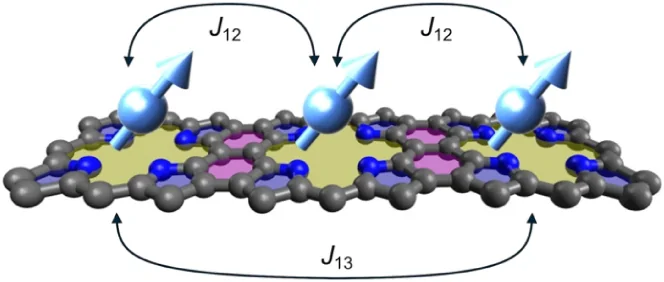

## Introduction

Molecular materials are important for the development of quantum technologies.^1–3^ In particular, π-conjugated molecular architectures provide a versatile means to engineer spin interactions over well-defined nanometric distances. In this context, graphene nanoribbons^4,5^ and porphyrin tapes^6–8^ illustrate how extended molecular frameworks can sustain efficient electronic communication, underscoring their promise as structurally precise quantum materials.^9^ Nevertheless, the realization of molecular systems that combine robust magnetic centers with long-range spin–spin communication remains a major challenge, largely because their rational design, synthesis, and characterization are inherently demanding.

One-dimensional spin chains are among the most promising candidates for magnetic information transfer.^10–12^ They exhibit collective quantum phenomena, including propagating magnetic excitations that behave as quasiparticles and are known as “spinons”. Despite their conceptual appeal, the experimental realization of linear spin chains with strong isotropic exchange interactions remains difficult. Organic materials are appealing for this purpose because they can support spin delocalization and entanglement over long distances.^13,14^ The few structurally defined organic spin chains reported so far have already revealed nontrivial magnetic states^15^ and cooperative spin behaviors.^12,16,17^ In most of these systems, however, the spins arise from organic radicals that are highly reactive, complicating their solution-phase manipulation and limiting their processability.

In contrast to organic radicals, metal spin centers are chemically stable and have rich magnetic functionality, considering the multitude of paramagnetic *d*- and *f*- metal ions.^18,19^ However, in most inorganic complexes the spin density is predominantly confined to the metal ion,^20^ which intrinsically limits long-range magnetic communication. Moreover, inorganic spin systems lack delocalized orbitals that would enable electric transport, and thus the possibility of electrical readout in a potential transistor-type device.^9^

Porphyrins offer a robust platform for creating molecular materials that combine a delocalized π-conjugated system with metal spin centers (Figure 1). Recently, a *meso,meso*-linked porphyrin trimer with two vanadyl porphyrins bridged by a free-base porphyrin has been reported.^21^ While in the ground state of this system the VO ions behave as two noninteracting spin centers (2 × [*S* = 1/2]), photoexcitation of the central free-base porphyrin generates a long-range ferromagnetically coupled system (*S* = 2).^21^ The superexchange coupling^22^ between electron spins in contiguous metal centers of porphyrin dimers depends on the degree of conjugation, as observed between Cu^II^,^23,24^ V^IV^,^25^ and lanthanide­(III) ions.^26^ Fe^III^ chloride porphyrins are known to exhibit a spin of *S* = 5/2 per high-spin *d* ^5^ iron center.^20,27^ Although edge-fused porphyrin nanoribbons are synthetically accessible,^28,29^ and a Cu^II^-containing edge-fused porphyrin trimer was reported 25 years ago,^30^ very little is known about the magnetic properties of these systems.

**Figure 1:**
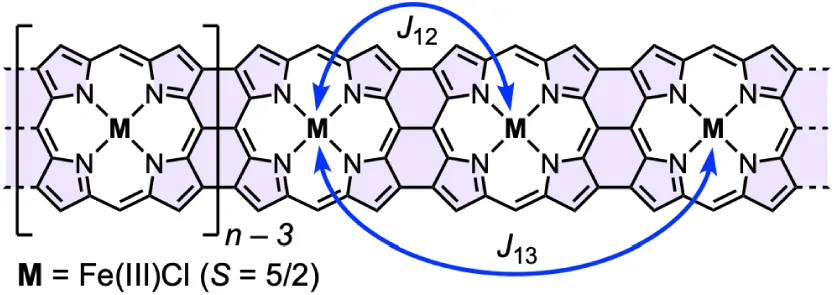
Structure of a triply fused porphyrin spin chain with *n* units. Nearest neighbor (*J*_12_) and second neighbor (*J*_13_) superexchange couplings mediated by a conjugated π-system.

Here, we synthesize and study the Fe^III^–Cl porphyrins presented in Scheme 1. The chemical design of this family enabled us to develop an adaptive model and to identify the most relevant interactions present in the trimeric systems. We demonstrate that spin–spin communication through a conjugated π-system is significant over nanometric distances (1.7 nm) and extends to nearest and second neighbors. This finding disentangles the effects of competing magnetic interactions and provides the basis for modeling longer spin chains of this type. In most studies of spin chains, it is assumed that exchange coupling between next nearest neighbors (*J*_13_) is negligible compared with *J*_12_,^31,32^ but here we show that *J*_12_ and *J*_13_ are similar in magnitude in edge-linked Fe^III^ porphyrin trimers. Furthermore, we show that *J*_12_ and *J*_13_ are both negative (antiferromagnetic coupling) which leads to spin frustration, since the competing 1–2 and 1–3 interactions cannot be simultaneously fulfilled.^33,34^

**Scheme 1:**
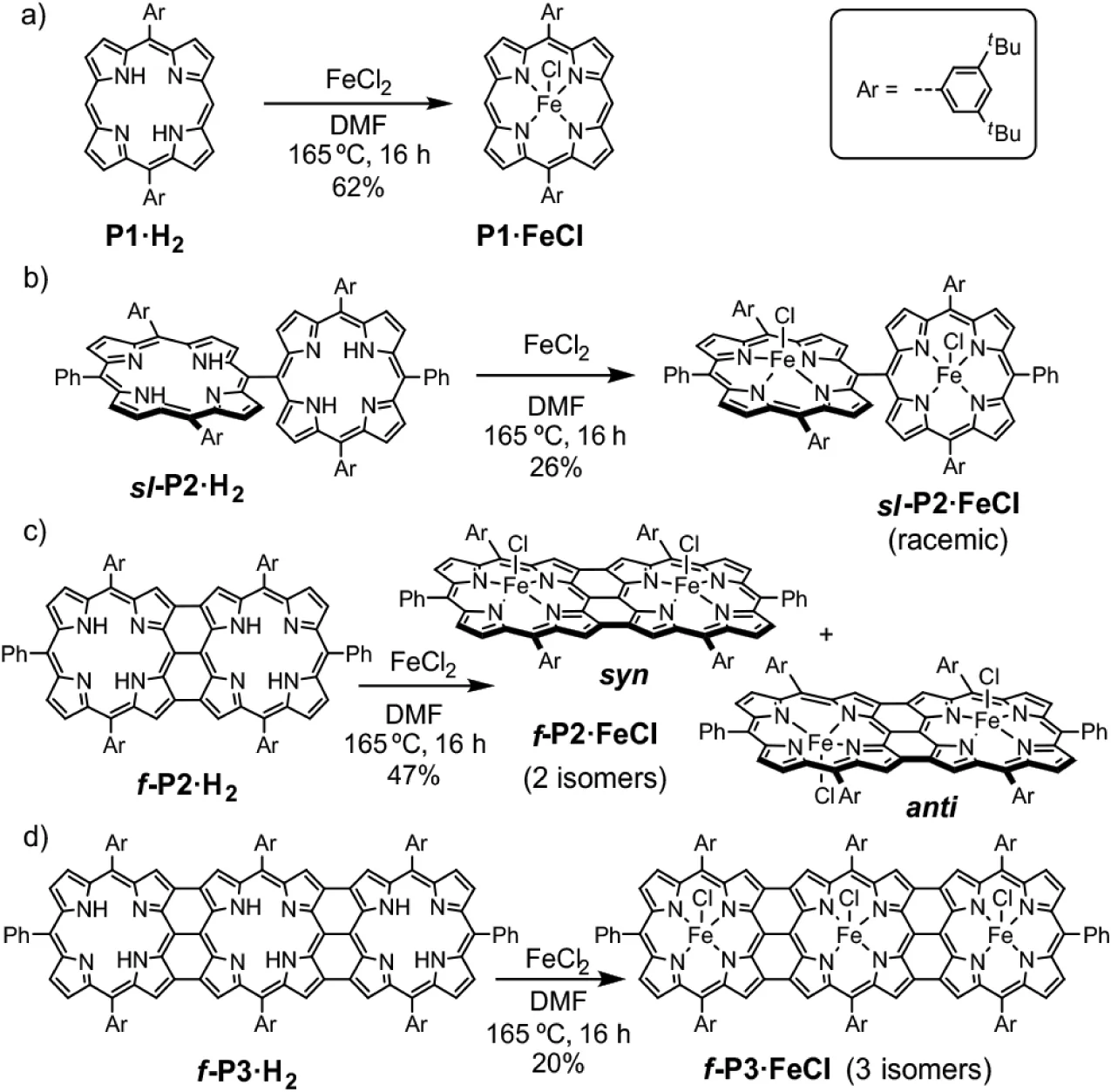
Synthesis of a) **P1·FeCl**, b) ***sl-*** **P2·FeCl**, c) ***f-*** **P2·FeCl** and d) ***f-*** **P3·FeCl**

## Results and Discussion

### Synthesis and Characterization

We synthesized compounds **P1·FeCl**, ***sl*** **-P2·FeCl**, and ***f*** **-P2·FeCl**, as models for understanding the trimer ***f*** **-P3·FeCl** and predicting the behavior of longer oligomers. They were synthesized following similar procedures, by heating the free-base porphyrins with FeCl_2_ in DMF, under an inert atmosphere for 16 h, followed by aerobic oxidation of Fe^II^ to Fe^III^, and purification by recrystallization.^35,36^ **P1·FeCl**, ***sl*** **-P2·FeCl,** ***f*** **-P2·FeCl** and ***f*** **-P3·FeCl** were isolated in yields of 62%, 26%, 47% and 20%, respectively (Scheme 1 and SI Section 1). We did not attempt to separate the *syn-* and *anti*-isomers of ***f*** **-P2·FeCl** and *f* **-P3·FeCl**, which arise because the chlorine atom sits on one face of the porphyrin. All compounds were characterized by ^1^H NMR, MALDI-MS, IR, UV–vis–NIR and EPR spectroscopy and SQUID magnetometry (see SI).

### X-ray Diffraction Analysis

**P1·FeCl**, ***sl*** **-P2·FeCl**, and ***f*** **-P2·FeCl** provided suitable crystals for X-ray diffraction analysis (Figure 2). The crystal structures of two other metal complexes of this porphyrin monomer have been reported previously (**P1·VO** and **P1·MnCl**).^25,37^ **P1·FeCl** presents the Fe^III^ ion 0.477 Å above the mean plane of the four nitrogen atoms, with Fe···Cl and Fe···N distances of 2.2110(8) and 2.070(5) Å, respectively, similarly to other Fe^III^–Cl porphyrins.^38^ ***sl*** **-P2·FeCl** has a torsion angle between the nitrogen planes of 86.9° and an Fe···Fe distance of 8.322 Å. Crystals of ***f*** **-P2·Fe** were grown from two different solvent systems, chloroform and 1,2-dichlorobenzene. In both cases, the crystals were of the *anti*-isomer, with a crystallographic inversion center at the center of the molecule. The core π-system of ***f*** **-P2·FeCl** is flat with Fe···Fe distances of 8.425 Å and 8.439 Å in the two pseudo polymorphs. The structural resemblance between ***f*** **-P2·FeCl** and ***f-*** **P3·FeCl** allows us to estimate the distance between the iron centers at the extremes of the molecule of ***f-*** **P3·FeCl** as 16.9 Å. It is interesting to compare the crystal structure of ***f*** **-P2·FeCl** with that of a *meso,β-*fused Fe^III^–Cl porphyrins dimer, which has an Fe···Fe distance of 9.08 Å.^39,40^ Both structures have *anti* stereochemistry.

**Figure 2:**
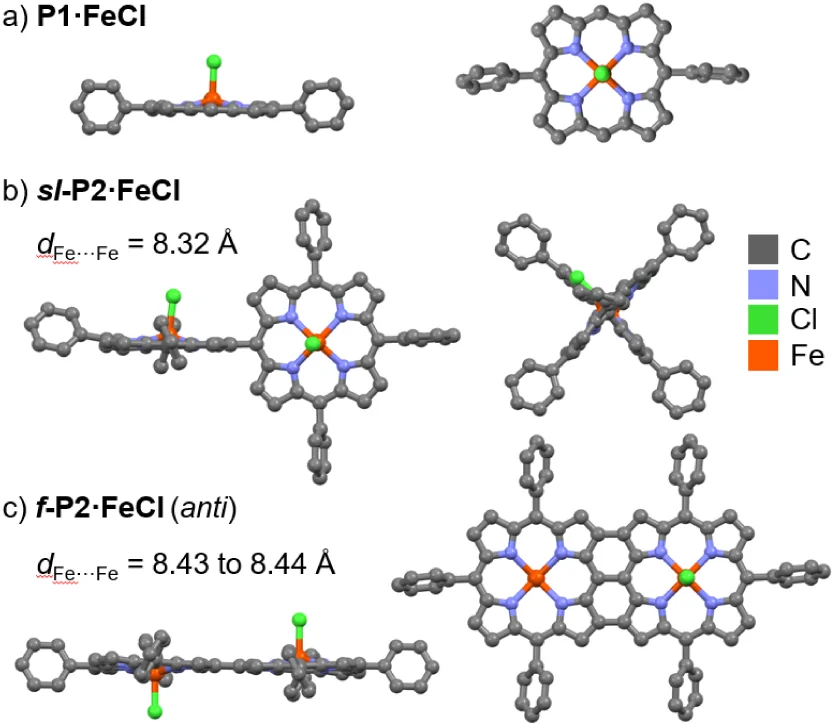
X-ray structure (two views) of a) **P1·FeCl**, b) ***sl-*** **P2·FeCl** and c) ***anti-f-*** **P2·FeCl** (hydrogen atoms, *t*-Bu groups and solvent molecules omitted for clarity).

### SQUID Magnetometry

SQUID experiments at *T* = 300 K and 0.1 T give a molar magnetic susceptibility (χ_M_ *T*) of 4.23 cm^3^ mol^–1^ K for **P1·FeCl**, 8.27 and 8.23 cm^3^ mol^–1^ K for ***sl*** **-P2·FeCl** and ***f*** **-P2·FeCl**, and 12.94 cm^3^ mol^–1^ K for ***f*** **-P3·FeCl**, in agreement with the presence of one, two and three *S* = 5/2 centers (Figure 3a). The variable-temperature SQUID data for **P1·FeCl**, ***sl-*** **P2·FeCl**, ***f*** **-P2·FeCl** and ***f*** **-P3·FeCl** were analyzed using various models. We evaluated the zero-field splitting *D*, considering both axial anisotropy and isotropic behavior, yielding similar *J* values for the dimeric and trimeric systems, even though the extracted *D* values differed substantially. We therefore adopted a model that assumes that the iron centers are isotropic and neglects dipolar coupling and hyperfine interactions, as their contributions are small compared to the zero-field splitting (*D*), and the nearest neighbor (*J*_12_) and second neighbor (*J*_13_) exchange couplings. This model corresponds to the following Hamiltonian:

$$H=\mu_{B}\overset{n}{\underset{i=1}{\sum }}Bg_{i}{Ŝ}_{i}+\overset{n}{\underset{i=1}{\sum }}{Ŝ}_{i}D{Ŝ}_{i}-2\overset{n-1}{\underset{i=1}{\sum }}{Ŝ}_{i}J_{12}{Ŝ}_{i+1}-2\overset{n-2}{\underset{i=1}{\sum }}{Ŝ}_{i}J_{13}{Ŝ}_{i+2}$$

where ***μ** **_B_*** is the Bohr magneton, *B* is the external magnetic field, ***Ŝ*** and ***Î*** are the electron and nucleus spin operators.

**Figure 3:**
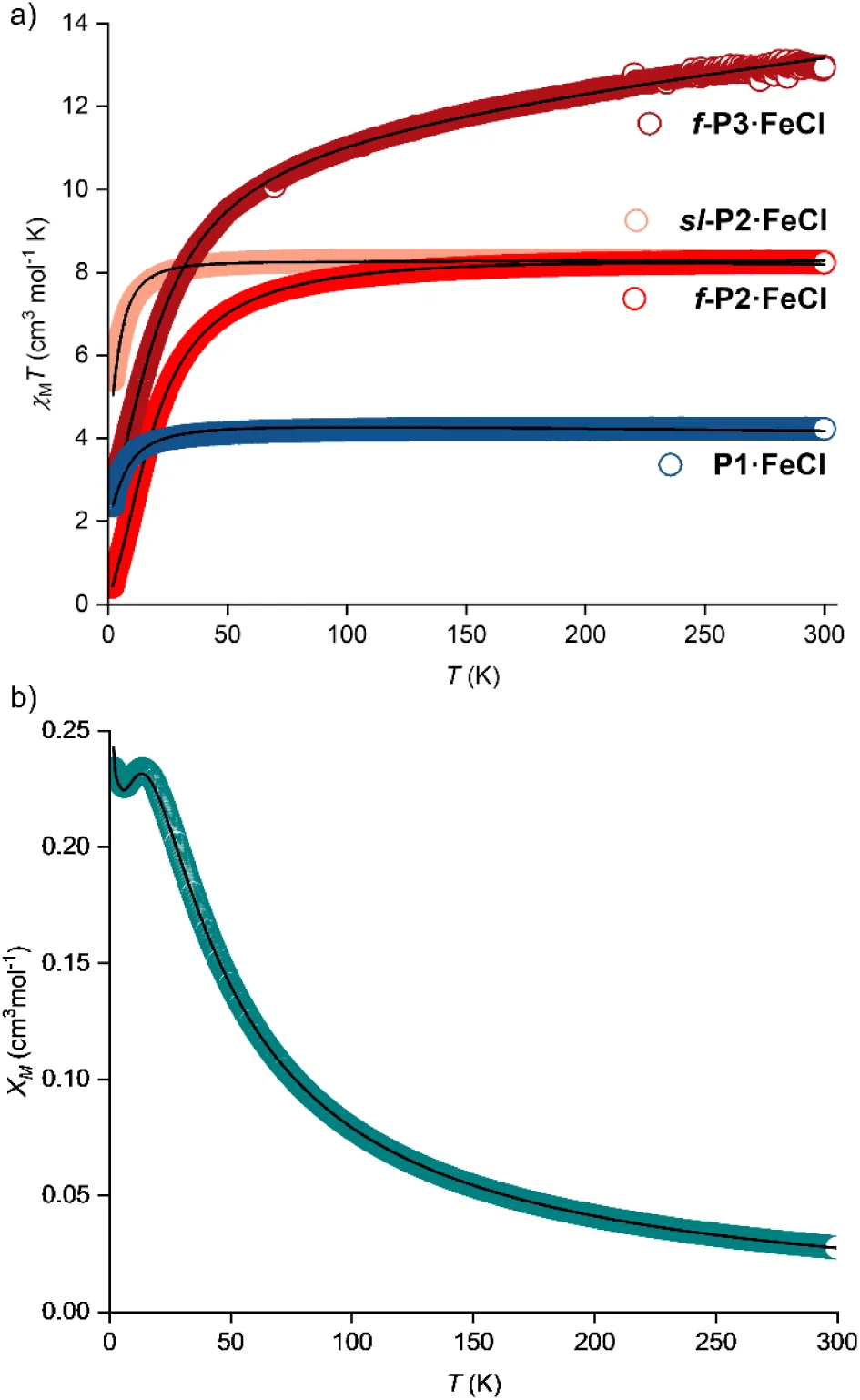
a) Magnetic susceptibility measurements of **P1·FeCl**, ***sl-*** **P2·FeCl**, ***f-*** **P2·FeCl** and *f-* **P3·FeCl**, and b) magnetization measurements of ***f-*** **P2·FeCl**, in solid state, at variable temperature (fitting lines in black).

The temperature-dependent magnetization data for **P1·FeCl** were fitted with *g* fixed at 2.00, as estimated from CW EPR (Figure S33), giving *D* = 99.2 ± 0.1 GHz (SI, Figure S37), in agreement with reported values for other Fe^III^–Cl porphyrins.^38,41^ For the singly linked ***sl*** **-P2·FeCl**, the data enabled us to test the proposed model Hamiltonian in a dimeric (*n* = 2) system. Fitting the data gave zero-field splittings on the two Fe centers of *D*_1_ = *D*_2_ = 73.75 ± 1.0 GHz. It was not possible to reliably determine the value of *J*_12_, but the data indicate that 0 < *J*_12_ < + 1 GHz (i.e., ferromagnetic coupling; Figure S38, Table S5). The positive sign of *J*_12_ extracted from the SQUID fittings is consistent with the DFT calculations (*J*_12,DFT_ = +0.3 ± 0.1 GHz) and the^1^ H NMR data (both included below). This weak ferromagnetic exchange can be attributed to the nearly perpendicular orientation of the porphyrin planes (Figure 2b), which minimizes π-orbital overlap between the two porphyrins and thus favors a ferromagnetic alignment.^42,43^

In the case of the triply linked ***f*** **-P2·FeCl** (Figures 3b and S39), reliable fitting of *J*_12_ was achieved, but determination of *D* was more challenging. To circumvent this issue, *D*_1_ and *D*_2_ were fixed at 73.75 GHz, as previously determined for ***sl*** **-P2·FeCl**. The resulting fit gave *J*_12_ = −32.98 ± 0.02 GHz, indicating strong antiferromagnetic coupling (Figure S39 and Table S4). This experimentally derived coupling constant is in agreement with DFT predictions (*J*_12,DFT_ = −40 ± 8 GHz, discussed below). The effect of this strong antiferromagnetic interaction is evident in the peak observed in the temperature-dependent magnetization curve at 13.6 K (Figure 3b).

Fitting the magnetization data for ***f*** **-P3·FeCl** was complicated by the presence of paramagnetic impurities (which may correlate with the fitted magnitude of *J*_13_) and by temperature-independent paramagnetism, both of which tend to obscure the intrinsic contributions of *J* and *D*. Therefore, as for the other compounds, the *g*-value was fixed at 2.00 for all three spins, and *D*_1_, *D*_2_, and *D*_3_ were set to 73.75 GHz. Under these conditions, the fitting yielded *J*_12_ = −24.28 ± 0.06 GHz and *J*_13_ = −10.8 ± 0.1 GHz (Figures S40, S41; Table S3). Thus, *J*_12_ in ***f-*** **P3·FeCl** is slightly weaker than in ***f-*** **P2·FeCl**, in agreement with the DFT-predicted trend (presented below).

### NMR Spectroscopy

The fast electron spin relaxation times of iron­(III) porphyrins lead to moderately sharp ^1^H NMR spectra (so-called paramagnetic NMR).^20^ The NMR chemical shift (δ) of compounds containing paramagnetic metal ions can be dissected into diamagnetic (δ_dia_) and paramagnetic (δ_para_) contributions. The diamagnetic shift can be estimated from a closely analogous diamagnetic compound, e.g., by replacing Fe^III^–Cl with Zn^II^. The paramagnetic shift results from the sum of contact (δ_con_, through bonds, π and σ) and dipolar (δ_dip_, through space or pseudocontact) terms. Therefore, the observed shift δ can be described by eq 1:

1 $$\delta =\delta_{\mathrm{dia}}+\delta_{\mathrm{para}}=\delta_{\mathrm{dia}}+\delta_{\mathrm{con}\left(\sigma \right)}+\delta_{\mathrm{con}\left(\pi \right)}+\delta_{\mathrm{dip}}$$

The spin density is defined as the difference in density between the spin-up and spin-down electrons.^44^ The contact term δ_con_ originates from delocalization of a small fraction of spin density onto the organic framework and spin polarization of the paired electrons.^45^ Both mechanisms occur simultaneously, and are described collectively as *spin polarization*. The contact contribution to the chemical shift (δ_con_), directly varies with the hyperfine coupling constant *A*_H_,^46^ which is proportional to the spin density on the carbon to which the observed proton is attached (ρ_c_) and the McConnell constant (*Q*),^45,47^ according to eq 2)­

2 $$A_{H}=Q\rho_{c}/2S$$

where *S* = 5/2. When spin polarization is propagated by *σ-*orbitals, *Q* > 0, whereas propagation through π-orbital gives *Q* < 0, modifying the sign of the resulting chemical shift (δ_con(σ)_ > 0 > δ_con(π)_).^45,47^ When two or more paramagnetic ions produce spin polarization, the combined effect depends on whether the sign is the same or opposite. If it is the same, the effect on the spin density is constructive, whereas if it is the opposite the effect is destructive.

The ^1^H NMR spectra of high-spin iron­(III) porphyrins have been widely studied.^20,27,36,46,48^ The chemical shift of hydrogen atoms at the periphery is mostly determined by a large contact contribution and much smaller dipolar and zero-field-splitting terms, especially at high temperatures since δ_dip_ ∝ *D*/*T* ^2^ whereas δ_con_ ∝ 1/*T*.^20,45,47^ The half-occupied $d_{x^{2}-y^{2}}$ orbital overlaps with the σ-skeleton (as in Cu^II^ porphyrins) whereas the half-occupied *d*_*xz*_ and *d*_*yz*_ orbitals overlap with the π-system.^45,47^ The ^1^H NMR spectrum of **P1·FeCl** (Figure 4a) is typical of a high-spin five-coordinate Fe^III^ porphyrin monomer, with a *meso*-resonance at negative chemical shift (−71.3 ppm, Table 1 and Figure S6), due to the π-mediated contact shift, and β-proton signals at high positive chemical shift (77.7 and 80.3 ppm for H_ex_ and H_exex_, hydrogen atoms respectively; Figure 4a) due to the *σ-*mediated contact shift. H_ex_ is shielded relative to H_exex_ by the proximal aryl group. Considering the chemical shift of the corresponding protons in **P1·Zn**,^49^ the experimental *σ-*contribution is δ_σ_(H_ex_) = 68.5 ppm (Table S1).

**Figure 4:**
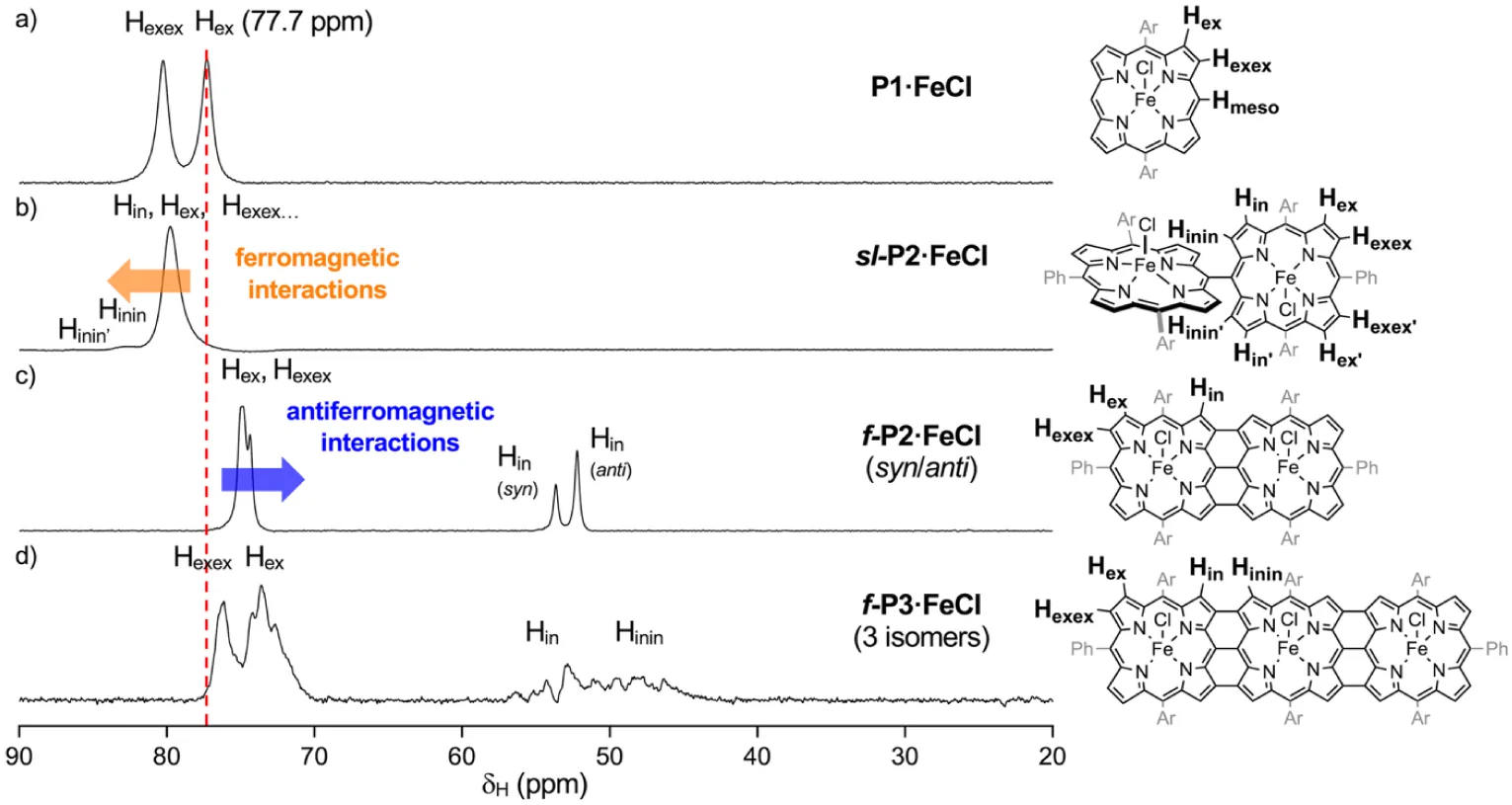
^1^H NMR spectra of a) **P1·FeCl**, b) ***sl-*** **P2·FeCl**, c) ***f-*** **P2·FeCl** and d) ***f-*** **P3·FeCl** (CDCl_3_ solution, 400 MHz, 25 °C).

**Table 1:** ^1^H NMR Chemical Shift (in ppm) of Zn^II^ and Fe^III^–Cl Porphyrins

M =	Zn^II^ experimental δ_H_				Fe^III^–Cl – experimental δ_H_				Fe^III^–Cl – DFT calculated δ_H_			
H =	H_inin_	H_in_	H_ex_	H_exex_	H_inin_	H_in_	H_ex_	H_exex_	H_inin_	H_in_	H_ex_	H_exex_
**P1·M**	-	10.4^a^	9.17	9.47	-	–71.3^a^	77.7	80.3	-	–83.2^a^	45.5	
***sl-*** **P2·M** ^b^	8.11	8.66	8.95	9.00	86.0	79.8	79.8	79.8	87.6	79.6	73.7	78.3
					83.0				87.2	79.5	73.5	77.95
***syn-*** **P2·M**	-	7.28	7.44	7.62	-	52.2	74.7	74.7	-	59.1	78.2	76.4
***anti-*** **P2·M**					-	53.7	74.7	74.7	-	57.9	77.3	76.4
***f-*** **P3·M** ^c^	6.23	6.97	7.52	7.53	46.4–51.0	51.0–56.8	72.8–74.2	76.2	37.1	43.9	59.8	61.7

a For **P1·M**, H_in_ protons refer to the H at the *meso* position (H_meso_).

b Cells with two values due to the symmetry break introduced by the Fe­(III)Cl center. The upper row refers to the protons closer to the iron atom in the other porphyrin (H_inin_, H_in_, H_ex_ and H_exex_), whereas the lower row refers to the protons further from the iron center in the other porphyrin (H_inin′_, H_in′_, H_ex′_ and H_exex′_).

c For ***f*** **-P3·FeCl**, DFT calculations gave minimal chemical shift differences between isomers, however, they strongly depend on the spin configuration. The calculated chemical shifts in this table are the average over all isomers and spin configurations.

The ^1^H NMR spectrum of ***sl-*** **P2·FeCl** (Figure 4b) can be assigned by comparison with that of a closely related compound reported by Wojaczyński et al.^50^ The internal protons H_inin_ and H_inin′_, give rise to extremely broad peaks at 86 and 83 ppm, due to their proximity to the iron center of the other porphyrin (see X-ray structure, Figure 2b). The additional downfield shift of the *β-*signals (Figure 4b), relative to H_ex_ of **P1·FeCl** (Figure 4a, vertical red line) is attributed to π-electron spin density from the second iron center. We estimate this contact contribution (δ_con_) to be 2.32 ppm (Figure 4b, Table S1). The sign of δ_con_ is opposite to that of the π-contribution within the porphyrin unit due to the s-orbital of the *meso–meso* bond, resulting in a positive hyperfine coupling with the remote iron center. Since the propagation pathway of the electron spin density is the same for both hyperfine and exchange couplings, the positive sign of δ_con_ (and the corresponding *A*_H_) reveals a ferromagnetic interaction between the iron centers (SI, Section 3). Ferromagnetic coupling was also reported in a similar heterometallic Cu^II^–V^IV^ singly linked porphyrin dimer.^42^

The ^1^H NMR spectrum of the mixture of *syn* and *anti* edge-fused porphyrin dimers ***f-*** **P2·FeCl** presents H_in_ protons at 53.7 and 52.2 ppm and the rest of the β-proton signals around 74.7 ppm. The assignment of the signals at 53.7 and 52.2 to H_in_ of *syn* and *anti* isomers is supported by the observation that these resonances coalesce when excess tetrabutylammonium chloride is added to the sample, as rapid exchange of chloride ligands interconverts the isomers.^50^ This assignment assumes that external protons are less influenced by the distal iron center, because the σ-term becomes negligible beyond four bonds.^51^ All β-protons are shifted upfield due to the constructive effect of the spin density from the other iron center, which is antiferromagnetic coupled, as predicted computationally and confirmed by SQUID magnetometry; see above. Conjecturing that the *anti*-isomer predominates due to its smaller dipole moment, the peaks at 53.7 and 52.2 ppm can be assigned to H_in_ of *syn* and *anti*-***f-*** **P2·FeCl**, respectively, based on their relative integral. The DFT calculated chemical shifts support this assignment (Table 1 and SI, Section 9). The following paramagnetic contributions from the adjacent iron center can be deduced from the ^1^H NMR of ***f-*** **P2·Zn**:^22^ δ_π1_ = −1.27 ppm (*syn*/*anti* H_ex_), δ_π2_ = −22.1 ppm (*syn* H_in_) and δ_π2_ = −23.6 ppm (*anti* H_in_) (Table S1).

The ^1^H NMR spectrum of ***f-*** **P3·FeCl** is expected to be complex due to the presence of the three stereoisomers. Considering the analysis of the previous compounds and our theoretical study (next section) we concluded the following assignment. H_exex_: 76.2 ppm, H_ex_: 74.2–72.8 ppm, H_in_: 56.8–51.0 ppm and H_inin_: 51.0–46.5 ppm. The chemical shift of H_inin_ below 50 ppm is expected due to the accumulative δ_σ_, δ_π1_and δ_π2_ contributions (Table S1, δ_dia_ from ***f-*** **P3·Zn** ^52^). The upfield shift in H_ex_ in ***f-*** **P3·FeCl** compared with ***f-*** **P2·FeCl** is consistent with an additional contribution to the spin density from the remote second neighboring iron center (δ_π3_ = −1.17 ppm), which is of the same sign and thus antiferromagnetic. This implies that the exchange couplings between the Fe^III^ centers are described by 0 > *J*_13_ > *J*_12_ (antiferromagnetic), as deduced from the theoretical study in the next section.

### Theoretical Calculation of ^1^H NMR Shifts and Exchange Coupling

We computed the NMR shifts for the iron­(III) porphyrins using broken-symmetry density functional theory (BS-DFT; Table 1, right-hand columns). The calculations were performed by weighting the populations of the Ising states according to the Boltzmann distribution and including the Zeeman effect at 298 K (M06–2X, SI Section 9). Good agreement was obtained between experimental and computational ^1^H NMR shifts (Table 1 and Figure S43) confirming the assignments of the experimental signals.

Exchange couplings (*J*) for ***sl-*** **P2·FeCl**, ***f-*** **P2·FeCl** and ***f-*** **P3·FeCl** were calculated using the broken symmetry approach, using a range of different density functional approximations (Figure 5a), including OX-B3LYP, which is optimized for edge-fused porphyrins.^53^ All the functionals predict the same signs for *J*_12_ and *J*_13_, and the magnitudes of these couplings are not strongly dependent on the choice of functional.^54^ The calculated exchange coupling in ***sl-*** **P2·FeCl** is ferromagnetic (as also deduced from the ^1^H NMR data above), with *J*_12,DFT_ = +0.3 ± 0.1 GHz. In ***f-*** **P2·FeCl**, all the functionals predict strong antiferromagnetic coupling (*J*_12,DFT_ = −40 ± 8 GHz). In the case of ***f-*** **P3·FeCl**, DFT predicts a reduction in the nearest neighbor interaction (*J*_12,DFT_ = −33 ± 8 GHz), which may be due to three-spin exchange,^55^ and the second-neighbor interaction (*J*_13,DFT_ = −11 ± 4 GHz). (These calculated *J* values are averaged over the five functionals listed in Figure 5a, and they are for the most stable *anti* and *anti-anti*-isomers; the *J* values are predicted to be insensitive to the stereochemistry – see Tables S6 and S7.)

**Figure 5:**
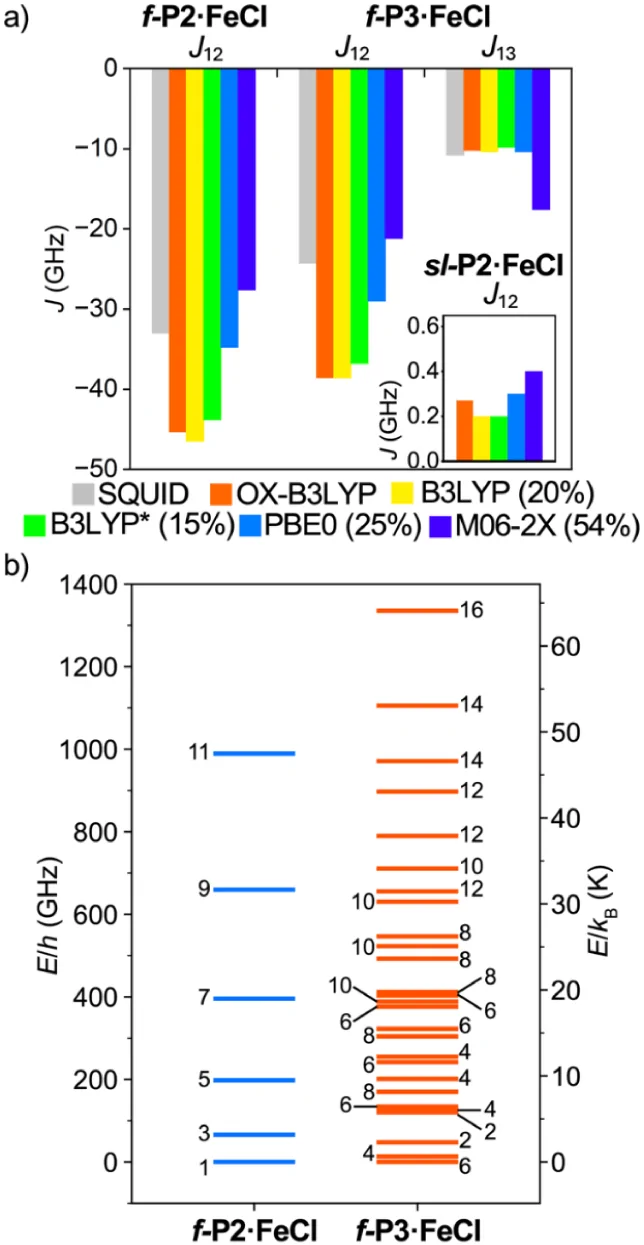
a) Exchange coupling values for *s* ***l-*P2·FeCl**, ***f-*P2·FeCl** and ***f-*P3·FeCl** calculated by the broken symmetry approach. b) Energy spectra (energy *E* in GHz and kelvin, at *B* = 0) of ***f-*P2·FeCl** (blue) and ***f-*P3·FeCl** (orange) obtained by applying the Heisenberg Hamiltonian with experimentally obtained values: *J*_12_ = −33.0 GHz for ***f-*P2·FeCl**; *J*_12_ = −24.3 and *J*_13_ = −10.8 GHz for *f-*P3·FeCl. States are labeled according to their multiplicity (2*S*+1).

The strong antiferromagnetic couplings may be explained by a superexchange^22^ mechanism, where *J* = 4*t* ^2^/*U*, with *t* as the (effective) through-bond coupling between the two sites and *U* as the Hubbard on-site repulsion. Since all metals have the same local environment, *U*_1_ = *U*_2_ = *U*_3_. Therefore *J*_12_ ≈ *J*_13_ implies *t*_12_ ≈ *t*_13_, meaning that the effective coupling is length-independent, which is consistent with strongly delocalized bridging orbitals. This suggests that superexchange proceeds through π-orbitals, which are known to support length-independent conductance in diamagnetic porphyrin nanoribbons.^7,56^

For spin systems to be useful in quantum technologies, the separation between their individual energy levels must be larger than thermal noise (*k*_B_ *T*).^57^ In Figure 5b, we show the low-energy energy spectra of ***f-*** **P2·FeCl** (left) and ***f-*** **P3·FeCl** (right), which imply that we could distinguish many energy states at a temperature of 4 K. We also note that the presence of a substantial *J*_13_ results in a nontrivial perturbation of the energy spectrum (see SI), i.e., it does not correspond to a simple reduction of the (effective) *J*_12_, as one might expect by considering the Ising Hamiltonian.

## Conclusions

Examination of the exchange coupling interactions between three linearly arranged spin centers embedded into a π-conjugated framework reveals, surprisingly, that the nearest neighbor interaction is comparable to the second nearest neighbor coupling (*J*_12_ = −24.3 GHz; *J*_13_ = −10.8 GHz). This means that theoretical models of longer porphyrin ribbon spin chains (Figure 1) must include at least second neighbor interactions. The spin polarization of the delocalized π-conjugated systems propagates magnetic interactions over nanometric distances in the edge fused oligomers ***f-*** **P2·FeCl** and ***f-*** **P3·FeCl**, as observed by the ^1^H NMR spectra, resulting in a much stronger magnetic coupling than in the singly linked dimer ***sl-*** **P2·FeCl** or in recently reported alkyne-linked porphyrin dimers.^58–60^ The effective superexchange mechanism^61^ results in the π-system behaving like a confined electron gas, used to connect spin centers in electronic devices.^62–66^ This is a key feature to enable spin–spin communication between localized spin centers at relatively long distances (>1 nm). This work expands the concept of *spin polarization* and demonstrates how this phenomenon can be exploited in molecular systems, which offer versatility by chemical design. It will be interesting to extend this study to longer linear porphyrin nanoribbons^67^ and cyclic nanobelts.^68^

## Experimental Section

### General Methods

All reactions were carried out under argon using oven-dried glassware. Commercial chemicals were purchased from ABCR GmbH, TCI, FluoroChem or Sigma-Aldrich and used without further purification. TLC was performed on Merck silica gel 60 F_254_ and chromatograms were visualized with UV light (254 and 365 nm). Column chromatography was performed on Merck silica gel 60 (ASTM 230–400 mesh). NMR spectra were recorded in commercially available deuterated solvents as indicated for each case at 298 K using Bruker AVIII HD 400 and Bruker AVIII HD 500 instruments. CDCl_3_ for NMR was stored over anhydrous K_2_CO_3_ and passed through neutral activated alumina (Brockmann I) before use. For paramagnetic Fe porphyrin samples, NMR spectra were recorded at 298 K on Bruker AVIII HD 400 or Bruker NEO 600 with broadband helium cryoprobe. Paramagnetic NMR spectra, with an additional wide-spectral width ^1^H NMR, were taken acquiring scans with acquisition time shortened to 0.1 s and a modified pulse angle of 10° when needed. Chemical shift values are referenced against residual solvent peaks. UV–vis-IR absorption spectra were recorded in dichloromethane solution at 298 K using a PerkinElmer Lambda 20 spectrophotometer and a Jasco V-770 UV–vis–NIR spectrophotometer in fused silica cuvettes with a 1 cm path length. Molar absorption coefficients are reported in L mol^–1^ cm^–1^. Infrared spectra were recorded in a PerkinElmer spectrum FT-IR spectrometer, using an ATR Top Plate – Diamond. MALDI-TOF mass spectra were measured at the EPSRC UK National Mass Spectrometry Facility (NMSF, Swansea) on a Bruker ultrafleXtreme MALDI-TOF/TOF instrument and Autoflex MALDI-TOF/TOF instrument. Size exclusion chromatography (SEC) was carried out using Bio-Rad Bio-Beads S-X1 (40–80 μm bead size). Semipreparative GPC was carried out on a Shimadzu recycling GPC system equipped with an LC-20 AD pump, SPD-20A UV detector and a set of JAIGEL 3H (20 × 600 mm) and JAIGEL 4H (20 × 600 mm) columns in toluene with a of 1% pyridine as the eluent at a flow rate of 3.5 mL/min. Compounds **P1·H**_**2**_,^69^ **P1·Zn**,^25^ **Ph-P1·Zn** (5,15-bis­(3,5-di-*tert*-butylphenyl)-10-phenyl zinc­(II) porphyrin),^25^ ***sl-*** **P2·H**_**2**_,^25^ and ***f-*** **P2·H**_**2**_,^25^ were prepared according to published procedures.

Variable-temperature direct current (dc) magnetic susceptibility data were collected on a Quantum Design MPMS-XL SQUID magnetometer equipped with a 7 T magnet and operating in the 2–300 K range, under a magnetic field of 1000 Oe (0.1 T). The diamagnetic contribution of the sample holder was subtracted from the raw data. Pascal’s constants were used to estimate the diamagnetic contribution of the different complexes, which were subtracted from the experimental susceptibilities to give the molar paramagnetic susceptibilities (χ_Μ_).

CW EPR spectra were recorded at 100 K of **P1·FeCl**, ***sl-*** **P2·FeCl**, and ***f-*** **P2·FeCl** using solid-state powder samples. These EPR spectra can be satisfactory simulated using similar parameters to those obtained from SQUID magnetometry.

### Computational Methodology

All structures were optimized at the M06–2X/def2-SVP level of theory, which was also used for computing chemical shifts. To investigate the effect of exact exchange (EE), broken symmetry calculations were done using a series of functionals: TPSSh (10% EE),S10 B3LYP* (15%),^70^ B3LYP (20%),^71^ OX-B3LYP (19–100%),^53^ PBE0 (25%),^72^ and M06–2X (54%).^73^ PBE0, OX-B3LYP, and M06–2X (which is highly empirical) produced good agreement with experiment, suggesting that ∼25% exchange is appropriate for these systems. *J* values were computed using Yamaguchi’s equation.^74,75^ All calculations were done using ORCA.^76^

### Synthetic Procedures

#### Synthesis of ***sl-*** **P3·Zn**

To a solution of **Ph-P1·Zn** (297 mg, 360 μmol, 6 equiv) and **P1·Zn** (45.0 mg, 60.0 μmol, 1 equiv) in dry CHCl_3_ (210 mL), another solution of AgPF_6_ (183 mg, 725 μmol, equiv) in dry acetonitrile (3.80 mL) and anhydrous dimethylacetamide (0.50 mL) was added. The reaction mixture was stirred at 30 °C for 18 h, then passed through a silica plug using petroleum ether:CHCl_3_ (1:1) as eluent. Volatiles were removed under reduced pressure and the residue was dissolved in CHCl_3_ (30 mL), and a solution of Zn­(OAc)_2_·2H_2_O (250 mg, 1.14 mmol, 19 equiv) in MeOH (10 mL) was added. The mixture was stirred at 50 °C for 1 h. Then, volatiles were removed and the residue was purified by flash column chromatography on silica gel using petroleum ether:CHCl_3_ (1:1) as eluent, followed by a gel exclusion chromatography column (2 m long, Bio-Rad Bio-Beads S-X1, 1% pyridine in toluene). Then, volatiles were removed and the residue was dissolved in CH_2_Cl_2_, washed three times with NaOAc/AcOH buffer solution (0.98 g NaOAc·3H_2_O and 1.88 mL AcOH dissolved in 200 mL H_2_O) and washed with NaHCO_3_ (sat.). The combined organic layers were concentrated and recrystallized from CHCl_3_:MeOH to give the desired product ***sl-*** **P3·Zn** as a pink powder (59 mg, 15% yield). ^1^H NMR (400 MHz, CDCl_3_) δ: 8.95 (s, 8H), 8.64 (d, *J* = 4.6 Hz, 4H), 8.59 (d, *J* = 4.7 Hz, 4H), 8.32–8.28 (m, 4H), 8.08 (d, *J* = 1.8 Hz, 8H), 8.06 (d, *J* = 4.6 Hz, 4H), 8.02 (d, *J* = 1.9 Hz, 4H), 7.98 (d, *J* = 4.7 Hz, 4H), 7.83–7.74 (m, 6H), 7.70 (t, *J* = 1.8 Hz, 4H), 7.56 (t, *J* = 1.9 Hz, 2H), 1.46 (s, 74H), 1.35 (s, 38H) ppm. MALDI-TOF *m*/*z* = 2400 (calcd for C_156_H_161_N_12_Zn_3_ [M + H]^+^: 2400).

#### Synthesis of ***f-*** **P3·Zn**

***sl-*** **P3·Zn** (21.0 mg, 8.75 μmol, 1 equiv), 4,5-dichloro-3,6-dioxocyclohexa-1,4-diene-1,2-dicarbonitrile (DDQ, 19.9 mg, 87.5 μmol, 10 equiv) and tris­(((trifluoromethyl)­sulfonyl)­oxy)­scandium (43.1 mg, 87.5 μmol, 10 equiv) were dissolved in dry toluene (6.00 mL). The mixture was degassed by three freeze–pump–thaw cycles, then stirred at 110 °C and monitored by TLC (on silica gel using petroleum ether:CH_2_Cl_2_, 1:1, as eluent). After 30 min, full conversion was observed, and the reaction was quenched by addition of Et_3_N (1 mL). Volatiles were removed under reduced pressure and the reaction crude was purified by flash column chromatography on silica gel using a gradient from petroleum ether:CH_2_Cl_2_ (1:1) to 5–20% Et_3_N in CH_2_Cl_2_ as eluent. Then, volatiles were removed and the residue was dissolved in CH_2_Cl_2_, washed three times with NaOAc/AcOH buffer solution (0.98 g NaOAc·3H_2_O and 1.88 mL AcOH dissolved in 200 mL H_2_O) and washed with NaHCO_3_ (sat.). The combined organic layers were concentrated and recrystallized from CHCl_3_:DMF and CHCl_3_:MeOH to give ***f-*** **P3·Zn** as a black powder (14 mg, 67% yield). ^1^H NMR (400 MHz, d6-benzene +1% d5-pyridine) δ: 7.96 (d, *J* = 4.5 Hz, 4H), 7.90 (d, *J* = 4.5 Hz, 4H), 7.86 (d, *J* = 2.2 Hz, 2H), 7.84 (d, *J* = 1.9 Hz, 8H), 7.71 (t, *J* = 1.9 Hz, 4H), 7.59 (d, *J* = 1.8 Hz, 2H), 7.57 (d, *J* = 1.8 Hz, 4H), 7.50 (s, 4H), 7.31 (d, *J* = 2.2 Hz, 4H), 7.30 (m, 4H), 6.80 (s, 4H), 1.37 (s, 72H), 1.36 (s, 36H) ppm. MALDI-TOF *m*/*z* = 2392 (calcd for C_156_H_153_N_12_Zn_3_ [*M*]^+^: 2392).

#### Synthesis of ***f-*** **P3·H**_**2**_

To a solution of ***f-*** **P3·Zn** (6.00 mg, 2.51 μmol, 1 equiv) in CHCl_3_ (2.0 mL), a 10% solution of trifluoroacetic acid in chloroform was dropwise added (180 μL, 2.35 mmol, in 1 mL of CHCl_3_, 900 equiv). The mixture was stirred at 20 °C for 3 h. Then, it was quenched with NaHCO_3_ (sat.) (20 mL) and extracted with CHCl_3_ (3 × 10 mL). The mixed organic layers were dried with Na_2_SO_4_ anhydrous and filtered. The combined organic layers were concentrated and recrystallized from CHCl_3_:MeOH to give ***f-*** **P3·H**_**2**_ as a black powder (4.7 mg, 84% yield). ^1^H NMR (400 MHz, CD_2_Cl_2_) δ: 7.78 (m, 8H), 7.65–7.55 (m, 26H), 7.41 (m, 8H), 1.42 (s, 72H), 1.41 (s, 36H) ppm. MALDI-TOF *m*/*z* = 2201 (calcd for C_156_H_159_N_12_ [M + H]^+^: 2201).

#### Synthesis of **P1·FeCl**

A solution of **P1·H**_**2**_ (380 mg, 553 μmol, 1 equiv) and iron­(II) chloride (631 mg, 4.98 mmol, 9 equiv) in dry DMF (130 mL) was heated at reflux temperature (165 °C) under argon atmosphere. After 3 h, DMF was partially removed under reduced pressure and CH_2_Cl_2_ was added (50 mL). The organic phase was washed with H_2_O (3 × 50 mL) and HCl (2 M aq., 50 mL). The organic layer was dried with Na_2_SO_4_, and solvent was removed under reduced pressure. The residue was recrystallized from dry CH_2_Cl_2_ and pentane to give **P1·FeCl** as a dark powder (267 mg, 62% yield). ^1^H NMR (400 MHz, CDCl_3_) δ: 80.26 (s, 4H), 77.29 (s, 4H), 8.11 (s, 2H), 6.12–6.11 (m, 2H), 5.04 (s, 2H), 2.64 (s, 18H), 2.13 (s, 18H), −71.26 (s, 2H) ppm. MALDI-TOF *m*/*z* = 775 (calcd for C_48_H_52_ClFeN_4_ [*M*]^+^: 775), 740 (calcd for C_48_H_52_FeN_4_ [*M – Cl*]^+^: 740). UV–vis–NIR (CH_2_Cl_2_) λ_max_ = 377 (ε = 0.08 μM^–1^ cm^–1^), 408­(ε = 0.13 μM^–1^ cm^–1^), 503 (ε = 0.02 μM^–1^ cm^–1^), 576 (ε = 0.01 μM^–1^ cm^–1^), 642 (ε = 0.01 μM^–1^ cm^–1^) nm. IR (ATR) (only selected signals) ν = 2964, 2906, 2964, 1595, 1365, 1250, 991 cm^–1^.

#### Synthesis of ***sl-*** **P2·FeCl**

A solution of ***sl-*** **P2·H**_**2**_ (62.0 mg, 40.7 μmol, 1 equiv) and iron­(II) chloride (103 mg, 814 μmol, 20 equiv) in dry DMF (20 mL) was heated at reflux temperature (165 °C) under argon atmosphere. After 3 h, DMF was partially removed under reduced pressure and CH_2_Cl_2_ was added (30 mL). The organic phase was washed with H_2_O (3 × 50 mL) and HCl (2 M aq., 50 mL). The organic layer was dried with Na_2_SO_4_, and solvent was removed under reduced pressure. The residue was precipitated by slow evaporation of pentane at −78 °C under constant flow of N_2_ (g) and decanted to give ***sl-*** **P2·FeCl** as a dark powder (18 mg, 26% yield). ^1^H NMR (400 MHz, CDCl_3_) δ: 86.00 (m, 16H), 83.00 (m, 16H), 79.76 (m, 16H), 13.64 (s, 4H), 12.40 (s, 4H), 8.64 (s, 4H, *H*_8_), 6.88 (s, 2H), 6.02 (s, 2H), 5.96 (s, 2H), 5.37 (s, 4H), 2.37 (s, 18H), 2.35 (m, 18H), 1.91 (s, 18H), 1.79 (s, 18H) ppm. MALDI-TOF *m*/*z* = 1703 (calcd for C_108_H_110_Cl_2_Fe_2_N_8_ [*M*]^+^: 1703), 1667 (calcd for C_108_H_110_ClFe_2_N_8_ [*M – Cl*]^+^: 1667). UV–vis–NIR (CH_2_Cl_2_) λ_max_ = 377 (ε = 0.12 μM^–1^ cm^–1^), 444 (ε = 0.16 μM^–1^ cm^–1^), 518 (ε = 0.04 μM^–1^ cm^–1^), 587 (ε = 0.01 μM^–1^ cm^–1^), 697 (ε = 0.01 μM^–1^ cm^–1^) nm. IR (ATR) (only selected signals) ν = 2959, 2901, 2868, 1596, 1361, 1000 cm^–1^.

#### Synthesis of ***f-*** **P2·FeCl**

A solution of ***f-*** **P2·H**_**2**_ (90 mg, 52.9 μmol, 1 equiv) and iron­(II) chloride (225 mg, 1.78 mmol, 30 equiv) in dry DMF (70 mL) was heated at reflux temperature (165 °C) under argon atmosphere. After 16 h, DMF was partially removed under reduced pressure and CH_2_Cl_2_ was added (50 mL). The organic phase was washed with H_2_O (3 × 50 mL) and HCl (2 M aq., 50 mL). The organic layer was dried with Na_2_SO_4_, and solvent was removed under reduced pressure. The residue was recrystallized from dry CH_2_Cl_2_ and pentane to give ***f-*** **P2·FeCl** as a dark powder (47 mg, 47% yield). ^1^H NMR (400 MHz, CDCl_3_) δ: 74.87–74.15 (m, 8H), 53.66–52.21 (2×s, 4H), 12.82 (s, 4H), 11.73 (s, 4H), 8.94 (s, 4H), 6.75 (s, 4H), 6.55–5.46 (m, 2H), 2.87–2.77 (2×s, 18H), 2.49–2.31 (2×s, 18H) ppm. MALDI-TOF *m*/*z* = 1698 (calcd for C_108_H_106_Cl_2_Fe_2_N_8_ [*M*]^+^: 1698), 1663 (calcd for C_108_H_106_ClFe_2_N_8_ [*M – Cl*]^+^: 1663), 1627 (calcd for C_108_H_106_Fe_2_N_8_ [*M – 2Cl*]^+^: 1627). UV–vis–NIR (CH_2_Cl_2_) λ_max_ = 412 (ε = 0.10 μM^–1^ cm^–1^), 559 (ε = 0.11 μM^–1^ cm^–1^), 1131 (ε = 0.02 μM^–1^ cm^–1^) nm. IR (ATR) (only selected signals) ν = 2964, 2911, 2868, 1596, 1485, 1005, 751 cm^–1^.

#### Synthesis of ***f-*** **P3·FeCl**

***f*** **-P3·H**_**2**_ (7.0 mg, 1 equiv) and FeCl_2_ (40.0 mg, 100 equiv) were dissolved in dry DMF (5.00 mL) by sonicating for 10 min. The reaction was heated at reflux temperature (165 °C) and stirred under argon overnight. The following day, the reaction was diluted with water (20 mL) and filtered. The residue on the filter paper was further washed with water (2 × 20 mL) and MeOH (2 × 20 mL). Then, the filter paper with the residue was dried under vacuum. Once it was dry, the filter paper with the residue was extracted by sonicating in CH_2_Cl_2_. This process was repeated up to the green color of the product was not observed in the solution. The combined organic layers (around 100 mL) were washed with HCl aqueous solution (2 M, 3 × 100 mL). The organic phase was dried with Na_2_SO_4_, filtered and dried under vacuum. The solid obtained (around 5 mg) was recrystallized in CHCl_3_:MeOH and filtered. The residue on the paper was further washed with MeOH (20 mL). Then, the filter paper with the residue was dried under vacuum and filter paper was extracted with CH_2_Cl_2_ and sonicated as described above. The combined organic layers (around 30 mL) were washed with HCl aqueous solution (2 M, 3 × 30 mL). The organic phase was dried with Na_2_SO_4_ filtered and dried under vacuum to give ***f-*** **P3·FeCl** as a dark-green powder (1.6 mg, 20% yield).^1^H NMR (400 MHz, CDCl_3_) δ: 76.15 (m), 74.20–72.80 (m), 56.82–51.00 (m,), 51.00–46.43 (m), 12.52 (d), 11.45 (d), 10.67–9.08 (m), 8.33 (m), 6.90 (s), 6.83–6.41 (m), 3.35–1.87 (m) ppm. MALDI-TOF *m*/*z* = 2468 (calcd for C_156_H_152_Cl_3_Fe_3_N_12_ [*M*]^+^: 2468), 2433 (calcd for C_156_H_152_Cl_2_Fe_3_N_12_ [*M – Cl*]^+^: 2433). UV–vis–NIR (CH_2_Cl_2_) λ_max_ = 412 (ε = 0.04 μM^–1^ cm^–1^), 640 (ε = 0.04 μM^–1^ cm^–1^), 1183 (ε = 0.01 μM^–1^ cm^–1^), 1405 (ε = 0.03 μM^–1^ cm^–1^) nm. IR (ATR) (only selected signals) ν = 2964, 2921, 2849, 1590, 1471, 1264, 1014, 804 cm^–1^.^77^

## Supplementary Material



## Notes

Synthetic procedures, EPR and SQUID data, UV–vis–NIR spectra, NMR, and DFT calculations of all compounds (PDF)

## Abbreviations

CW: continuous-wave
DFT: density functional theory
EPR: electron paramagnetic resonance
SQUID: superconducting quantum interference device
