# Supplementary material for: π‑Conjugation Mediates Competing J 12 and J 13 Interactions at the Nanometric Scale in Linear Fe(III) Porphyrin Trimers
Source: Inorg Chem. 2026 Aug 5;65(32):19001–10. doi: 10.1021/acs.inorgchem.6c03219 (PMC13488401; doi:10.1021/acs.inorgchem.6c03219)
Supplement: Supplementary file 1 [file ic6c03219_si_001.pdf]

Supporting Information for:

π-Conjugation Mediates Competing J_{12} and J_{13} Interactions at the Nanometric Scale in Linear Fe(III) Porphyrin Trimers

Iago Pozo,[a,b]* Lidao Li,[a] Ramón Torres-Cavanillas,[c,d] Wojciech Stawski,[a] William K. Myers,[e] Michael Slota,[c] Igor Rončević,[a,f]* Lapo Bogani[c,d]* and Harry L. Anderson[a]*

[a] Department of Chemistry, Oxford University, Chemistry Research Laboratory, Oxford OX1 3TA, United Kingdom.

[b] Centro Singular de Investigación en Química Biolóxica e Materiais Moleculares (CIQUS) and Departamento de Química Orgánica, Universidade de Santiago de Compostela, Santiago de Compostela, 15782, Spain.

[c] Department of Materials, Oxford University, Oxford OX1 3PH, United Kingdom.

[d] Department of Chemistry and Department of Physics, University of Florence, Florence, 50019, Italy.

[e] Centre for Advanced ESR, Department of Chemistry, University of Oxford, Inorganic Chemistry Laboratory, South Parks Road, Oxford OX1 3QR, United Kingdom.

[f] Department of Chemistry, The University of Manchester, Manchester M13 9PL, U.K.

Email addresses of corresponding authors: iago.pozo.miguez@usc.es, igor.roncevic@manchester.ac.uk, lapo.bogani@materials.ox.ac.uk, harry.anderson@chem.ox.ac.uk

Table of Contents

1. Experimental Procedures S2
2. NMR Spectra S9
3. Paramagnetic NMR Spectra S11
4. UV-vis-NIR and IR Spectra S19
5. MALDI Mass Spectra S23
6. X-Ray Crystallography S27
7. EPR Spectroscopy S31
8. SQUID Magnetometry S33
9. Computational Details S39
10. References S41

1. Experimental Procedures

1.1. General methods

All reactions were carried out under argon using oven-dried glassware. Commercial chemicals were purchased from ABCR GmbH, TCI, FluoroChem or Sigma-Aldrich and used without further purification. TLC was performed on Merck silica gel 60 F_{254} and chromatograms were visualized with UV light (254 and 365 nm). Column chromatography was performed on Merck silica gel 60 (ASTM 230–400 mesh). NMR spectra were recorded in commercially available deuterated solvents as indicated for each case at 298 K using Bruker AVIII HD 400 and Bruker AVIIIHD 500 instruments. $CDCl_3$ for NMR was stored over anhydrous K_2CO_3 and passed through neutral activated alumina (Brockmann I) before use. For paramagnetic Fe porphyrin samples, NMR spectra were recorded at 298 K on Bruker AVIII HD 400 or Bruker NEO 600 with broadband helium cryoprobe. Paramagnetic NMR spectra, with an additional wide-spectral width ^{1}H NMR, were taken acquiring scans with acquisition time shortened to 0.1 s and a modified pulse angle of 10° when needed. Chemical shift values are referenced against residual solvent peaks. UV-vis-IR absorption spectra were recorded in dichloromethane solution at 298 K using a Perkin-Elmer Lambda 20 spectrophotometer and a Jasco V-770 UV-vis-NIR spectrophotometer in fused silica cuvettes with a 1 cm pathlength. Molar absorption coefficients are reported in L mol^{-1} cm^{-1}. Infrared spectra were recorded in a Perkin-Elmer spectrum FT-IR spectrometer, using an ATR Top Plate - Diamond. MALDI-TOF mass spectra were measured at the EPSRC UK National Mass Spectrometry Facility (NMSF, Swansea) on a Bruker ultrafleXtreme MALDI-TOF/TOF instrument and Autoflex MALDI-TOF/TOF instrument. Size exclusion chromatography (SEC) was carried out using Bio-Rad Bio-Beads S-X1 (40 – 80 µm bead size). Semi-preparative GPC was carried out on a Shimadzu Recycling GPC system equipped with a LC-20 AD pump, SPD-20A UV detector and a set of JAIGEL 3H (20 × 600 mm) and JAIGEL 4H (20 × 600 mm) columns in toluene with a of 1% pyridine as the eluent at a flow rate of 3.5 mL/min. Compounds **P1·H₂**,[S1] **P1·Zn**,[S2] **Ph-P1·Zn**,[S2] ***sl*-P2·H₂**,[S2] and ***f*-P2·H₂**,[S2] were prepared according to literature procedures.

1.2. Synthesis of Fe(III)Cl porphyrins

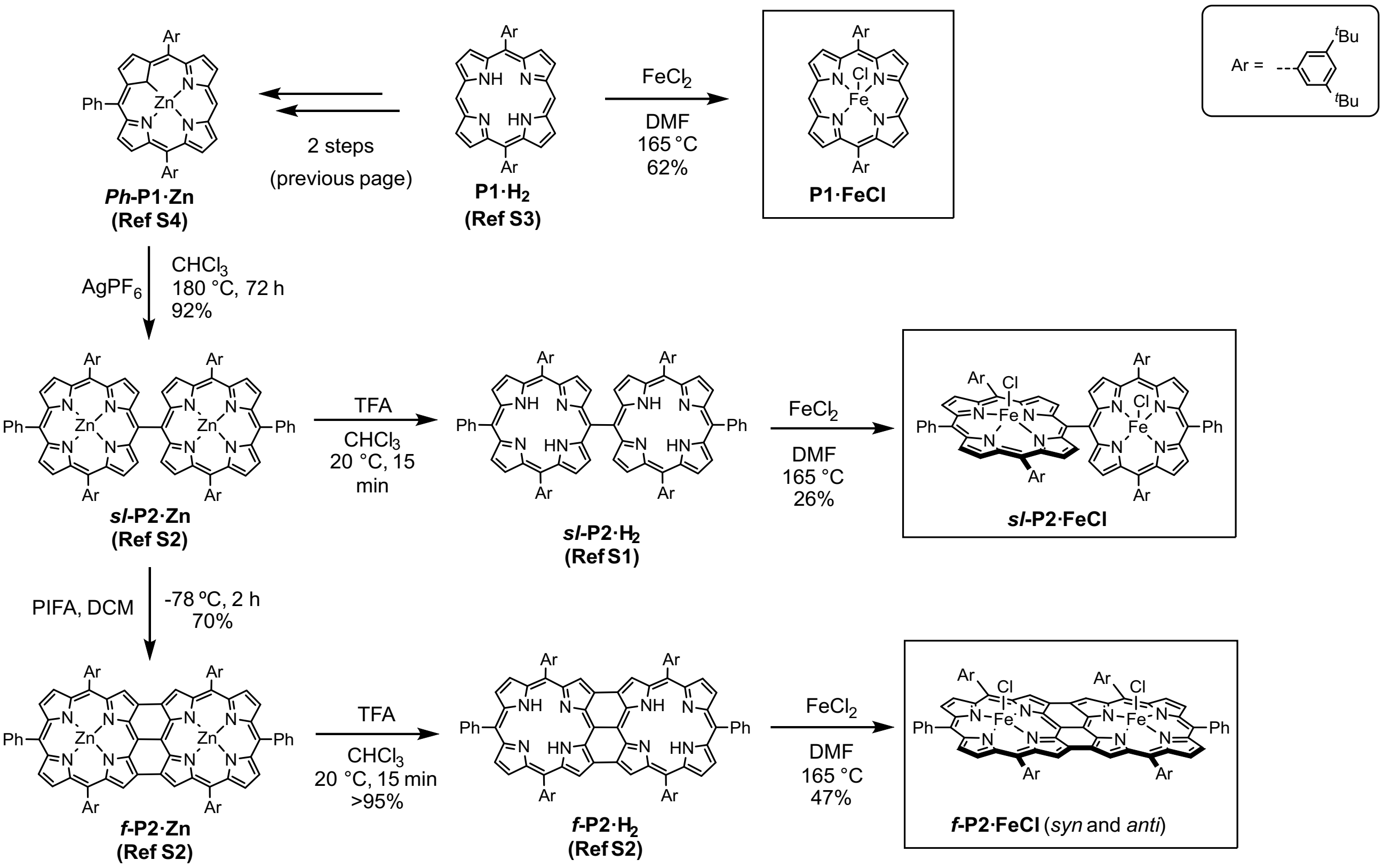

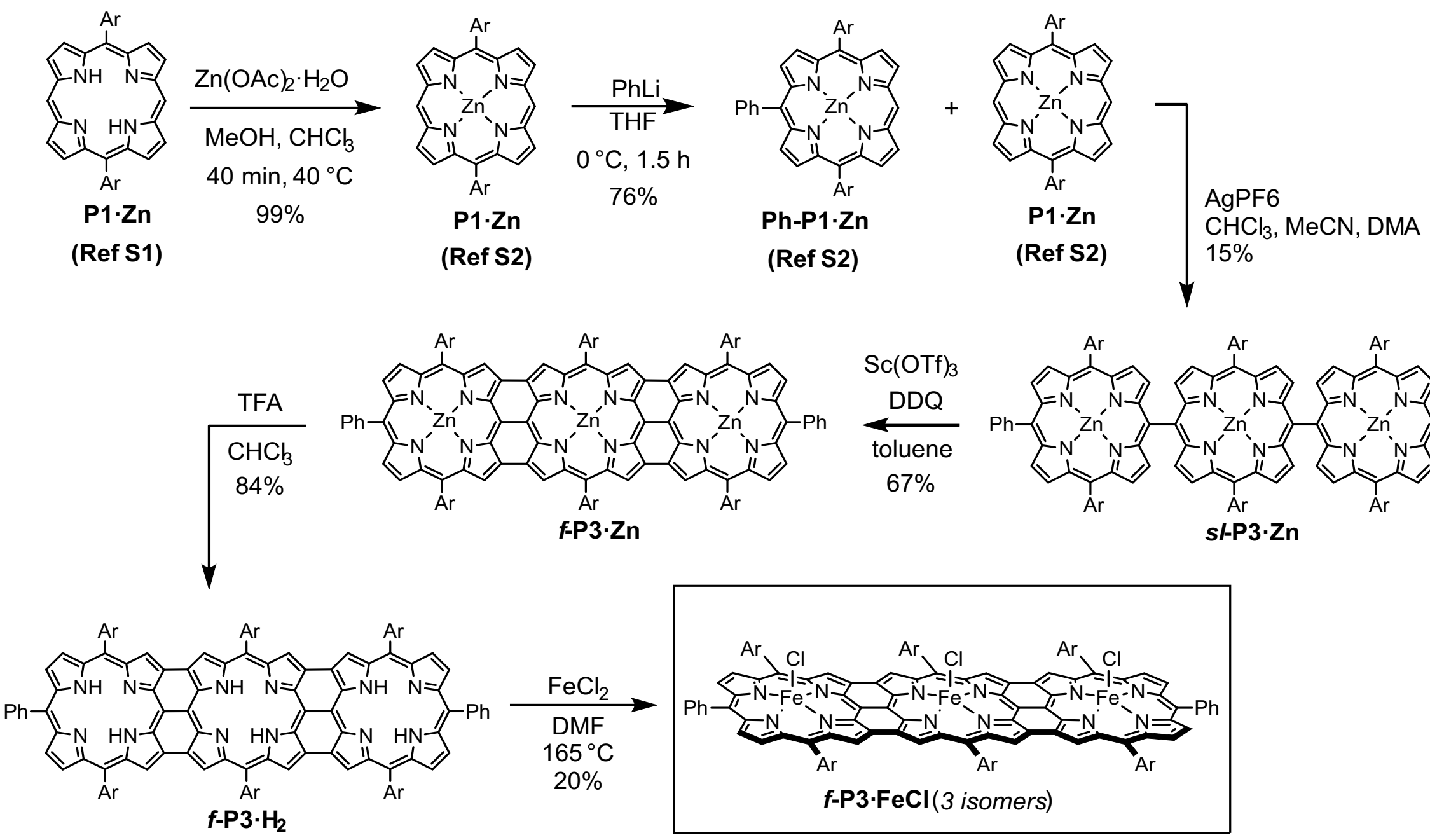

1.3. Experimental Synthetic Procedures (with reaction schemes and assignments)

Synthesis of ***sl*-P3·Zn**

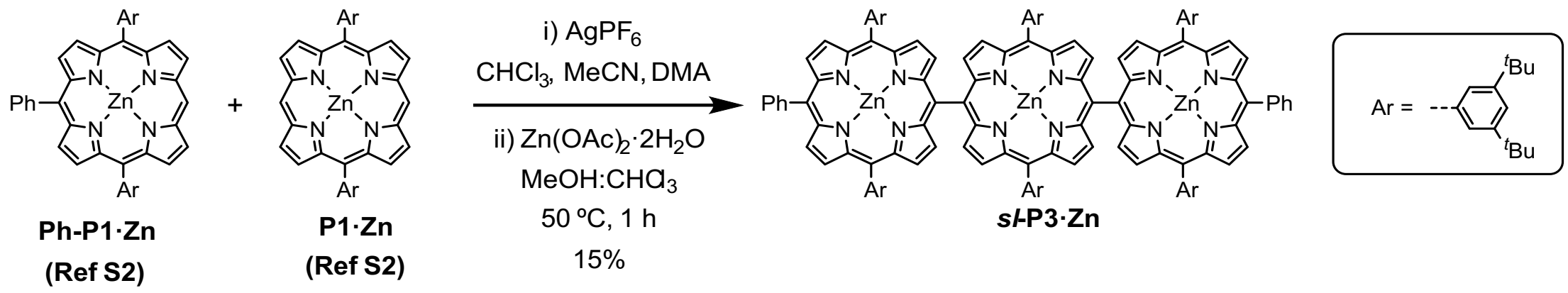

To a solution of **Ph-P1·Zn** (297 mg, 360 µmol, 6 equiv.) and **P1·Zn** (45.0 mg, 60.0 µmol, 1 equiv.) in dry $CHCl_3$ (210 mL), another solution of $AgPF_6$ (183 mg, 725 µmol, equiv.) in dry acetonitrile (3.80 mL) and anhydrous dimethylacetamide (0.50 mL) was added. The reaction was stirred at 30 °C for 18 hours. Then, the reaction mixture was passed through a silica plug using petroleum ether: $CHCl_3$ (1:1) as eluent. Volatiles were removed under reduced pressure and the residue was dissolved in $CHCl_3$ (30 mL), and a solution of $Zn(OAc)_2{\cdot}2H_2O$ (250 mg, 1.14 mmol. 19 equiv.) in MeOH (10 mL) was added. The mixture was stirred at 50 °C for 1 hour. Then, volatiles were removed and the residue was purified by flash column chromatography on silica gel using petroleum ether:$CHCl_3$ (1:1) as eluent, followed by 2 m long gel exclusion chromatography column using Bio-Rad Bio-Beads S-X1 beads (1% pyridine in toluene). Then, volatiles were removed and the residue was dissolved in CH_2Cl_2, washed three times with NaOAc/AcOH buffer solution (0.98 g $NaOAc{\cdot}3H_2O$ and 1.88 mL AcOH dissolved in 200 mL H_2O) and washed with $NaHCO_3$ (sat). The combined organic layers were concentrated and recrystallized from $CHCl_3$:MeOH to give the desired product ***sl*-P3·Zn** as a pink powder (59 mg, 15% yield).

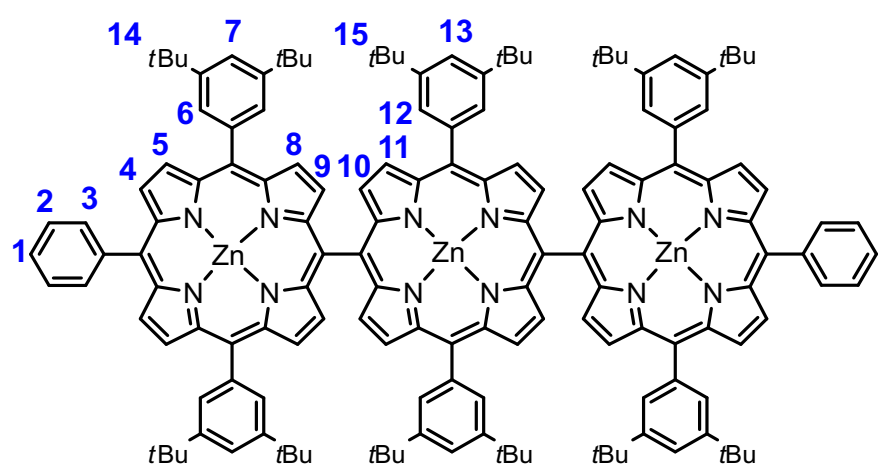

^{1}H NMR (400 MHz, CDCl3) δ: 8.95 (s, 8H, $H_{4,5}$), 8.64 (d, J = 4.6 Hz, 4H, H_8), 8.59 (d, J = 4.7 Hz, 4H, H_9), 8.32 – 8.28 (m, 4H, H_3), 8.08 (d, J = 1.8 Hz, 8H, H_6), 8.06 (d, J = 4.6 Hz, 4H, H_{10}), 8.02 (d, J = 1.9 Hz, 4H, H_{12}), 7.98 (d, J = 4.7 Hz, 4H, H_{10}), 7.83 – 7.74 (m, 6H, $H_{1,2}$), 7.70 (t, J = 1.8 Hz, 4H, H_7), 7.56 (t, J = 1.9 Hz, 2H, H_{13}), 1.46 (s, 74H, H_{14}), 1.35 (s, 38H, H_{15}) ppm. **MALDI-TOF** m/z = 2400 (calcd for $C_{156}H_{161}N_{12}Zn_3$ $[M+H]^+$: 2400).

Synthesis of ***f*-P3·Zn**

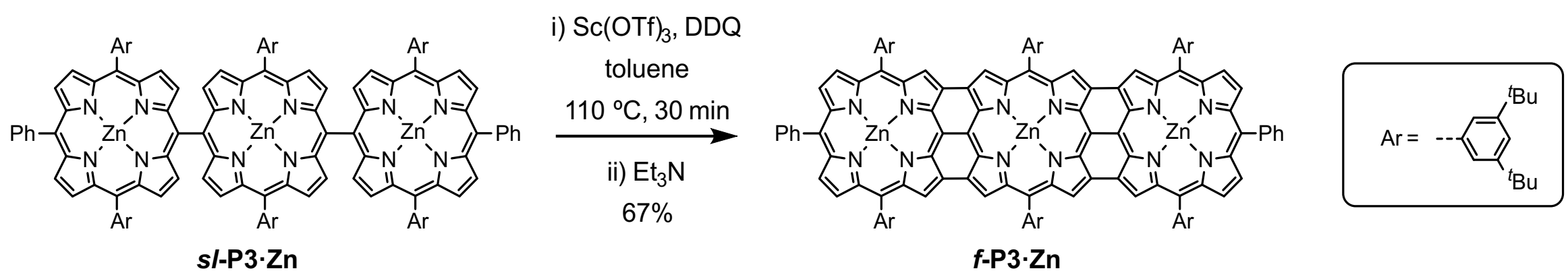

***sl*-P3·Zn** (21.0 mg, 8.75 µmol, 1 equiv.), 4,5-dichloro-3,6-dioxocyclohexa-1,4-diene-1,2-dicarbonitrile (DDQ, 19.9 mg, 87.5 µmol, 10 equiv.) and tris(((trifluoromethyl)sulfonyl)oxy)scandium (43.1 mg, 87.5 µmol, 10 equiv.) were dissolved in dry toluene (6.00 mL). The mixture was degassed by 3 freeze-pump-thaw cycles.

Then, the reaction mixture was stirred at 110 °C and monitored by TLC (on silica gel using petroleum other:CH_2Cl_2, 1:1, as eluent). After 30 min, full conversion was observed, and the reaction was quenched by addition of Et_3N (1 mL). Volatiles were removed under reduced pressure and the reaction crude was purified by flash column chromatography on silica gel using a gradient from petroleum ether:CH_2Cl_2 (1:1) to 5–20% Et_3N in CH_2Cl_2 as eluent. Then, volatiles were removed and the residue was dissolved in CH_2Cl_2, washed three times with NaOAc/AcOH buffer solution (0.98 g NaOAc·$3H_2O$ and 1.88 mL AcOH dissolved in 200 mL H_2O) and washed with $NaHCO_3$ (sat). The combined organic layers were concentrated and recrystallized from $CHCl_3$:DMF and $CHCl_3$:MeOH to give ***f*-P3·Zn** as a black powder (14 mg, 67% yield).

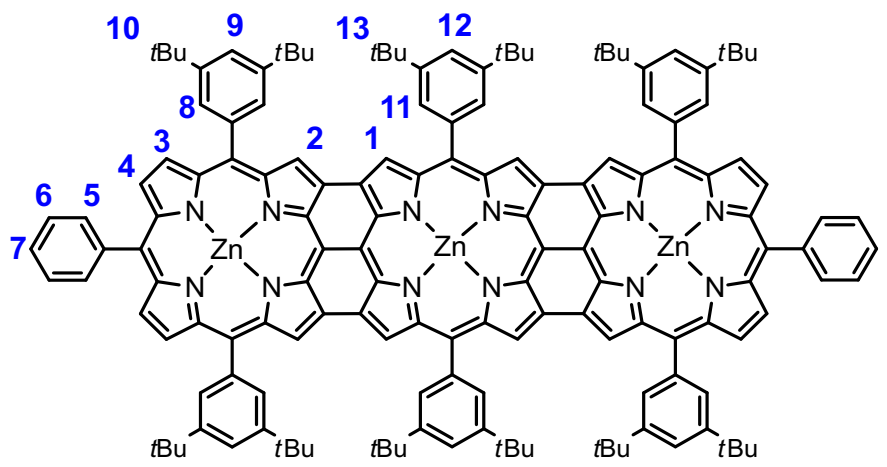

^{1}H NMR (400 MHz, d6-benzene + 1% d5-pyridine) δ: 7.96 (d, *J* = 4.5 Hz, 4H, ***H*₄**), 7.90 (d, *J* = 4.5 Hz, 4H, ***H*₃**), 7.86 (d, *J* = 2.2 Hz, 2H, ***H*₇**), 7.84 (d, *J* = 1.9 Hz, 8H, ***H*₈**), 7.71 (t, *J* = 1.9 Hz, 4H, ***H*₉**), 7.59 (d, *J* = 1.8 Hz, 2H, ***H*₁₂**), 7.57 (d, *J* = 1.8 Hz, 4H, ***H*₁₁**), 7.50 (s, 4H, ***H*₂**), 7.31 (d, *J* = 2.2 Hz, 4H, ***H*₆**), 7.30 (m, 4H, ***H*₅**), 6.80 (s, 4H, ***H*₁**), 1.37 (s, 72H, ***H*₁₀**), 1.36 (s, 36H, ***H*₁₃**) ppm. **MALDI-TOF** *m/z* = 2392 (calcd for $C_{156}H_{153}N_{12}Zn_3$ $[M]^+$: 2392).

Synthesis of ***f*-P3·H₂**

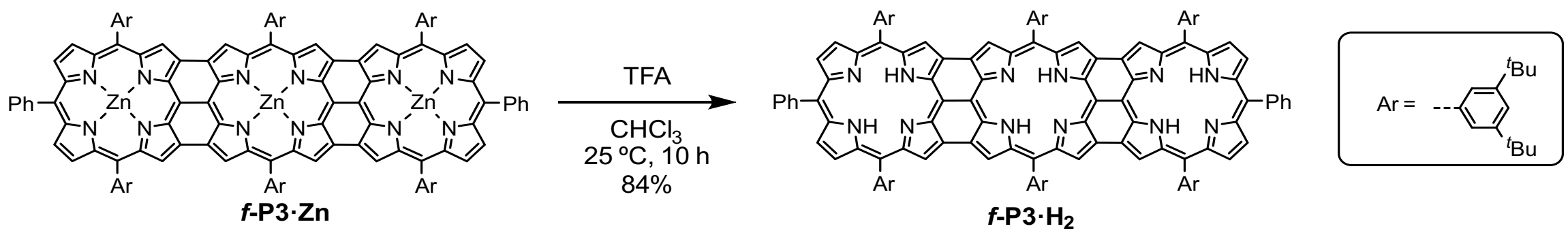

To a solution of ***f*-P3·Zn** (6.00 mg, 2.51 µmol, 1 equiv.) in $CHCl_3$ (2.0 mL), a 10% solution of trifluoroacetic acid in chloroform was dropwise added (180 µL, 2.35 mmol, in 1 mL of $CHCl_3$, 900 equiv.). The mixture was stirred at 20 °C for 3 h. Then, it was quenched with $NaHCO_3$ (sat) (20 mL) and extracted with $CHCl_3$ (3 x 10 mL). The mixed organic layers were dried with Na_2SO_4 anhydrous and filtered. The combined organic layers were concentrated and recrystallized from $CHCl_3$:MeOH to give ***f*-P3·H₂** as a black powder (4.7 mg, 84% yield).

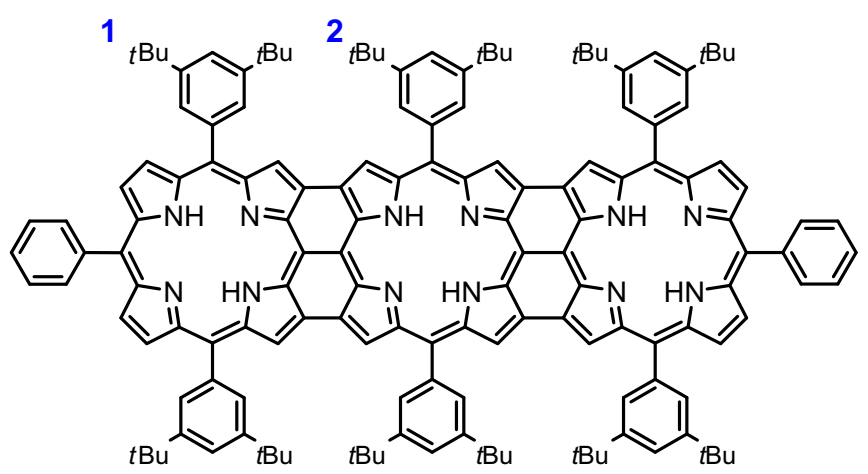

^{1}H NMR (400 MHz, CD_2Cl_2) δ: 7.78 (m, 8H), 7.65 – 7.55 (m, 26H), 7.41 (m, 8H), 1.42 (s, 72H, ***H*₁**), 1.41 (s, 36H, ***H*₂**) ppm. **MALDI-TOF** m/z = 2201 (calcd for $C_{156}H_{159}N_{12}$ $[M +H]^+$: 2201).

Synthesis of **P1·FeCl**

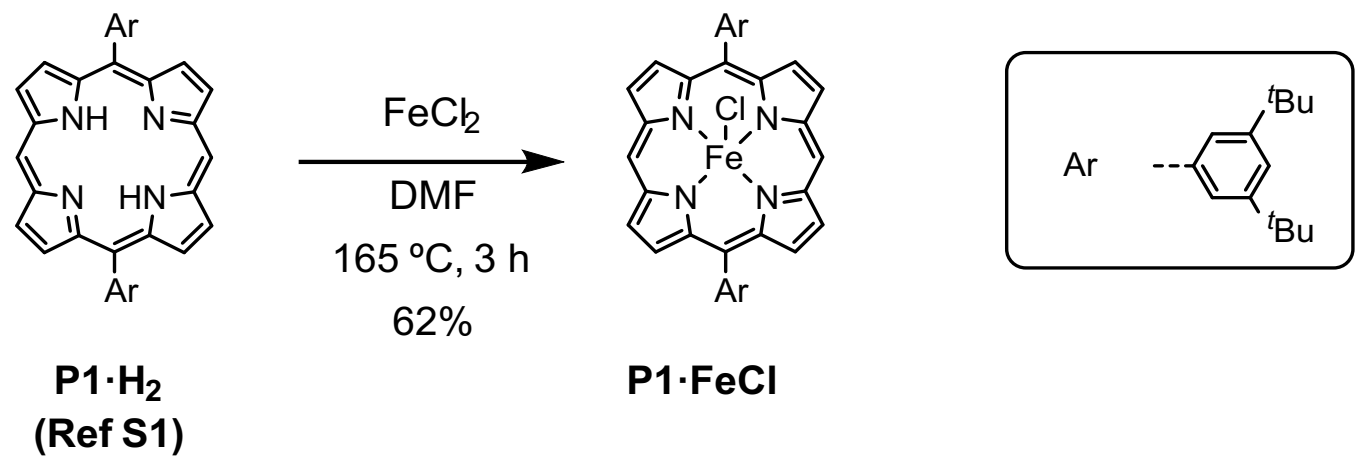

A solution of **P1·H₂** (380 mg, 553 µmol, 1 equiv.) and iron(II) chloride (631 mg, 4.98 mmol, 9 equiv.) in dry DMF (130 mL) was heated at reflux temperature (165 °C) under argon atmosphere. After 3 hours, DMF was partially removed under reduced pressure and CH_2Cl_2 was added (50 mL). The organic phase was washed with H_2O (3 × 50 mL) and HCl (2 M, 50 mL). The organic layer was dried with Na_2SO_4, and solvent was removed under reduced pressure. The residue was recrystallized from dry CH_2Cl_2 and pentane to give **P1·FeCl** as a dark powder (267 mg, 62% yield).

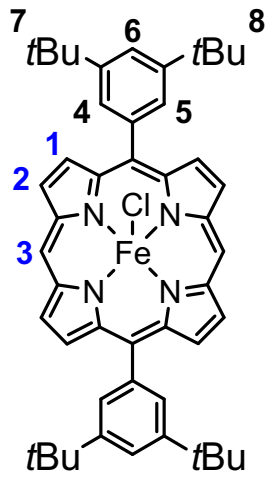

^{1}H NMR (400 MHz, $CDCl_3$) δ: 80.26 (s, 4H, ***H_2***), 77.29 (s, 4H, ***H_1***), 8.11 (s, 2H, ***H_4***), 6.12 – 6.11 (m, 2H, ***H_6***), 5.04 (s, 2H, ***H_5***), 2.64 (s, 18H, ***H_7***), 2.13 (s, 18H, ***H_8***), -71.26 (s, 2H, ***H_3***) ppm. **MALDI-TOF** *m/z* = 775 (calcd for $C_{48}H_{52}ClFeN_4$ $[M]^+$: 775), 740 (calcd for $C_{48}H_{52}FeN_4$ $[M\text{-}Cl]^+$: 740). **UV-vis-NIR** (CH_2Cl_2) λ_{max} = 377 (Ɛ = 0.08 µM^{-1} cm^{-1}), 408(Ɛ = 0.13 µM^{-1} cm^{-1}), 503 (Ɛ = 0.02 µM^{-1} cm^{-1}), 576 (Ɛ = 0.01 µM^{-1} cm^{-1}), 642 (Ɛ = 0.01 µM^{-1} cm^{-1}) nm. **IR (ATR)** (only selected signals) ν = 2964, 2906, 2964, 1595, 1365, 1250, 991 cm^{-1}.

Synthesis of ***sl*-P2·FeCl**

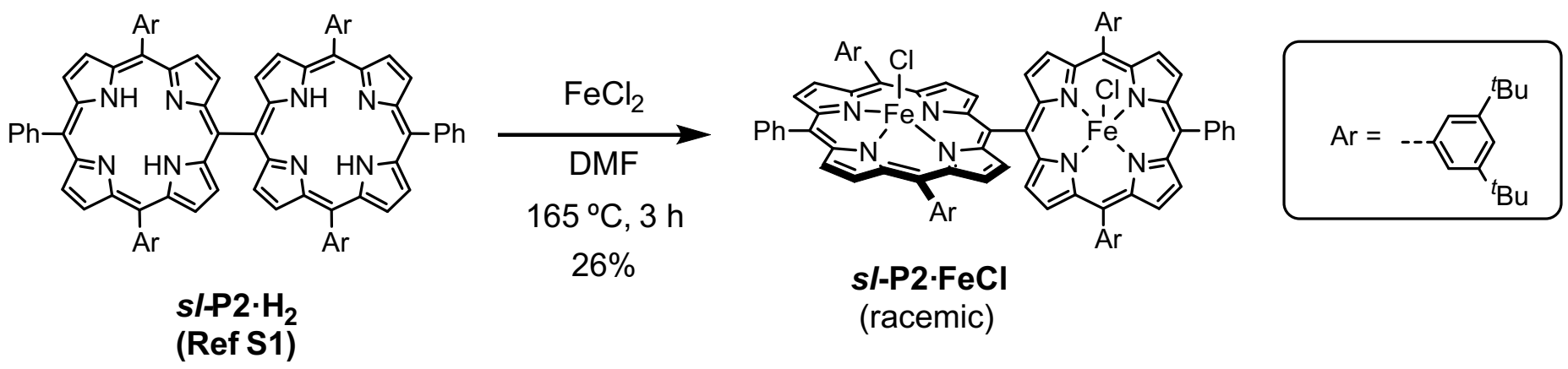

A solution of ***sl*-P2·H₂** (62.0 mg, 40.7 µmol, 1 equiv.) and iron(II) chloride (103 mg, 814 µmol, 20 equiv.) in dry DMF (20 mL) was heated at reflux temperature (165 °C) under argon atmosphere. After 3 hours, DMF was partially removed under reduced pressure and CH_2Cl_2 was added (30 mL). The organic phase was washed with H_2O (3 x 50 mL) and HCl (2 M, 50 mL). The organic layer was dried with Na_2SO_4, and solvent was removed under reduced pressure. The residue was precipitated by slow evaporation of pentane at –78 °C under constant flow of N_2 (g) and decanted to give ***sl*-P2·FeCl** as a dark powder (18 mg, 26% yield).

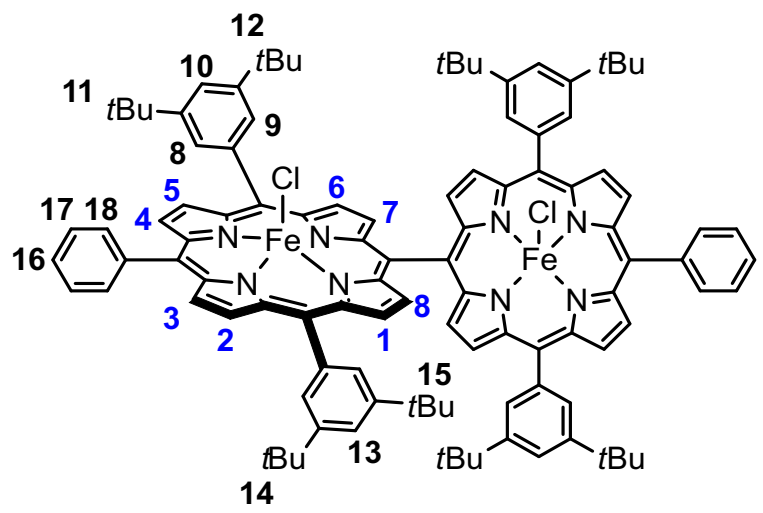

^{1}H NMR (400 MHz, $CDCl_3$) δ: 86.00 (m, 16H, ***H*$_8$**), 83.00 (m, 16H, ***H*$_7$**), 79.76 (m, 16H, ***H*$_{1-6}$**), 13.64 (s, 4H, ***H*$_{17}$**), 12.40 (s, 4H, ***H*$_{18}$**), 8.64 (s, 4H, ***H*$_8$**), 6.88 (s, 2H, ***H*$_{16}$**), 6.02 (s, 2H, ***H*$_{10}$**), 5.96 (s, 2H, ***H*$_{13}$**), 5.37 (s, 4H, ***H*$_9$**), 2.37 (s, 18H, ***H*$_{11}$**, 2.35 (m, 18H, ***H*$_{14}$**), 1.91 (s, 18H, ***H*$_{12}$**), 1.79 (s, 18H, ***H*$_{15}$**) ppm. **MALDI-TOF** *m/z* = 1703 (calcd for $C_{108}H_{110}Cl_2Fe_2N_8$ $[M]^+$: 1703), 1667 (calcd for $C_{108}H_{110}ClFe_2N_8$ $[M\text{-}Cl]^+$: 1667). **UV-vis-NIR** (CH_2Cl_2) λ_{max} = 377 (Ɛ = 0.12 μM^{-1} cm^{-1}), 444 (Ɛ = 0.16 μM^{-1} cm^{-1}), 518 (Ɛ = 0.04 μM^{-1} cm^{-1}), 587 (Ɛ = 0.01 μM^{-1} cm^{-1}), 697 (Ɛ = 0.01 μM^{-1} cm^{-1}) nm. **IR (ATR)** (only selected signals) ν = 2959, 2901, 2868, 1596, 1361, 1000 cm^{-1}.

Synthesis of ***f*-P2·FeCl**

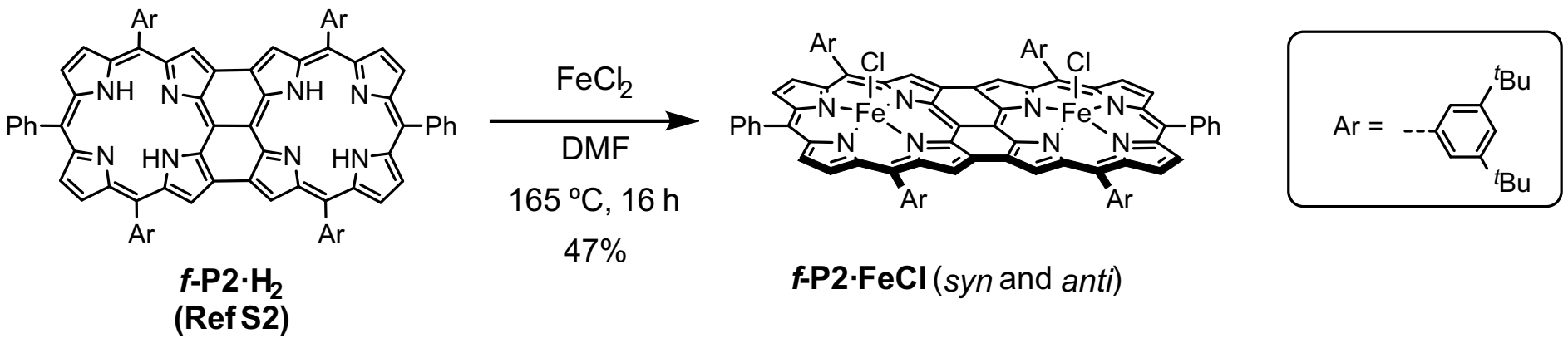

A solution of ***f*-P2·H$_2$** (90 mg, 52.9 µmol, 1 equiv.) and iron(II) chloride (225 mg, 1.78 mmol, 30 equiv.) in dry DMF (70 mL) was heated at reflux temperature (165 °C) under argon atmosphere. After 16 hours, DMF was partially removed under reduced pressure and CH_2Cl_2 was added (50 mL). The organic phase was washed with H_2O (3 x 50 mL) and HCl (2 M, 50 mL). The organic layer was dried with Na_2SO_4, and solvent was removed under reduced pressure. The residue was recrystallized from dry CH_2Cl_2 and pentane to give ***f*-P2·FeCl** as a dark powder (47 mg, 47% yield).

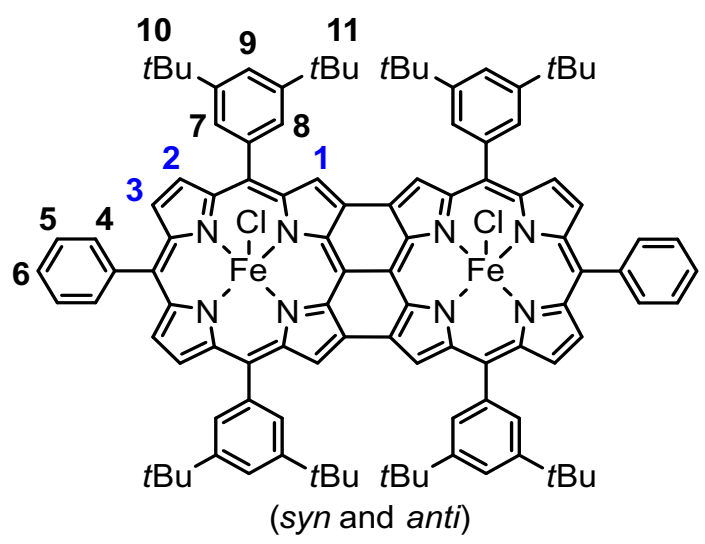

^{1}H NMR (400 MHz, $CDCl_3$) δ: 74.87– 74.15 (m, 8H, ***H*$_{2,3}$**), 53.66-52.21 (2xs, 4H, ***H*$_1$**), 12.82 (s, 4H, ***H*$_5$**), 11.73 (s, 4H, ***H*$_4$**), 8.94 (s, 4H, ***H*$_7$**), 6.75 (s, 4H, ***H*$_9$**), 6.55 – 5.46 (m, 2H, ***H*$_6$**), 2.87 - 2.77 (2xs, 18H, ***H*$_{10}$**), 2.49 – 2.31 (2xs, 18H, ***H*$_{11}$**) ppm. **MALDI-TOF** *m/z* = 1698 (calcd for $C_{108}H_{106}Cl_2Fe_2N_8$ $[M]^+$: 1698), 1663 (calcd for $C_{108}H_{106}ClFe_2N_8$ $[M\text{-}Cl]^+$: 1663), 1627(calcd for $C_{108}H_{106}Fe_2N_8$ $[M\text{-}2Cl]^+$: 1627). **UV-vis-NIR** (CH_2Cl_2) λ_{max} = 412 (Ɛ = 0.10 μM^{-1} cm^{-1}), 559 (Ɛ = 0.11 μM^{-1} cm^{-1}), 1131 (Ɛ = 0.02 μM^{-1} cm^{-1} nm. **IR (ATR)** (only selected signals) ν = 2964, 2911, 2868, 1596, 1485, 1005, 751 cm^{-1}.

Synthesis of ***f*-P3·FeCl**

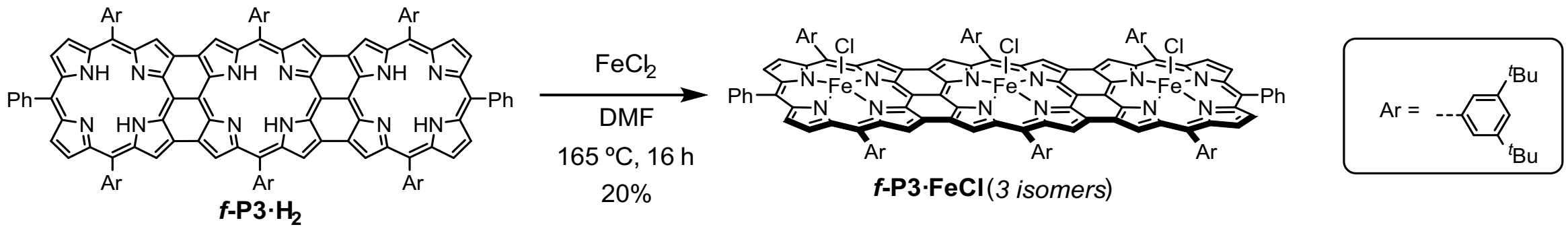

***f*-P3·H₂** (7.00 mg, 1 equiv.) and $FeCl_2$ (40.0 mg, 100 equiv.) were dissolved in dry DMF (5.00 mL) by sonicating for 10 minutes. The reaction was heated at reflux temperature (165 ºC) and stirred under argon overnight. The following day, the reaction was diluted with water (20 mL) and filtered. The residue on the filter paper was further washed with water (2 x 20 mL) and MeOH (2 x 20 mL). Then, the filter paper with the residue was dried under vacuum. Once it was dry, the filter paper with the residue was extracted by sonicating in CH_2Cl_2. This process was repeated up to the green color of the product was not observed in the solution. The combined organic layers (around 100 mL) were washed with HCl aqueous solution (2M, 3 x 100 mL). The organic phase was dried with Na_2SO_4, filtered and dried under vacuum. The solid obtained (around 5 mg) was recrystallized in $CHCl_3$:MeOH and filtered. The residue on the paper was further washed with MeOH (20 mL). Then, the filter paper with the residue was dried under vacuum and filter paper was extracted with CH_2Cl_2 and sonicated as described above. The combined organic layers (around 30 mL) were washed with HCl aqueous solution (2M, 3 x 30 mL). The organic phase was dried with Na_2SO_4 filtered and dried under vacuum to give ***f*-P3·FeCl** as a dark-green powder (1.6 mg, 20% yield).

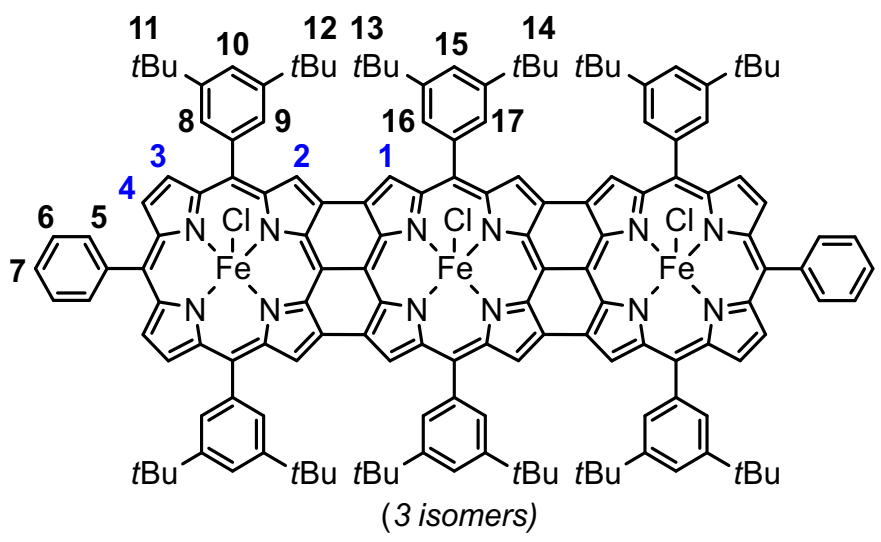

^{1}H NMR (400 MHz, $CDCl_3$) δ: 76.15 (m, ***H₄***), 74.20 – 72.80 (m, ***H₃***), 56.82 – 51.00 (m, ***H₂***), 51.00 – 46.43 (m, ***H₁***), 12.52 (d, ***H₆***), 11.45 (d, ***H₅***), 10.67 – 9.08 (m, ***H₈,₁₆***), 8.33 (m, ***H₁₀,₁₅***), 6.90 (s, ***H₇***), 6.83 – 6.41 (m, ***H₉,₁₇***), 3.35 – 1.87 (m, ***H₁₁ to ₁₄***) ppm. **MALDI-TOF** *m/z* = 2468 (calcd for $C_{156}H_{152}Cl_3Fe_3N_{12}$ [*M*]$^+$: 2468), 2433 (calcd for $C_{156}H_{152}Cl_2Fe_3N_{12}$ [*M-Cl*]$^+$: 2433). **UV-vis-NIR** (CH_2Cl_2) λ_{max} = 412 (Ɛ = 0.04 µM^{-1} cm^{-1}), 640 (Ɛ = 0.04 µM^{-1} cm^{-1}), 1183 (Ɛ = 0.01 µM^{-1} cm^{-1}), 1405 (Ɛ = 0.03 µM^{-1} cm^{-1}) nm. **IR (ATR)** (only selected signals) ν = 2964, 2921, 2849, 1590, 1471, 1264, 1014, 804 cm^{-1}.

2. NMR Spectra

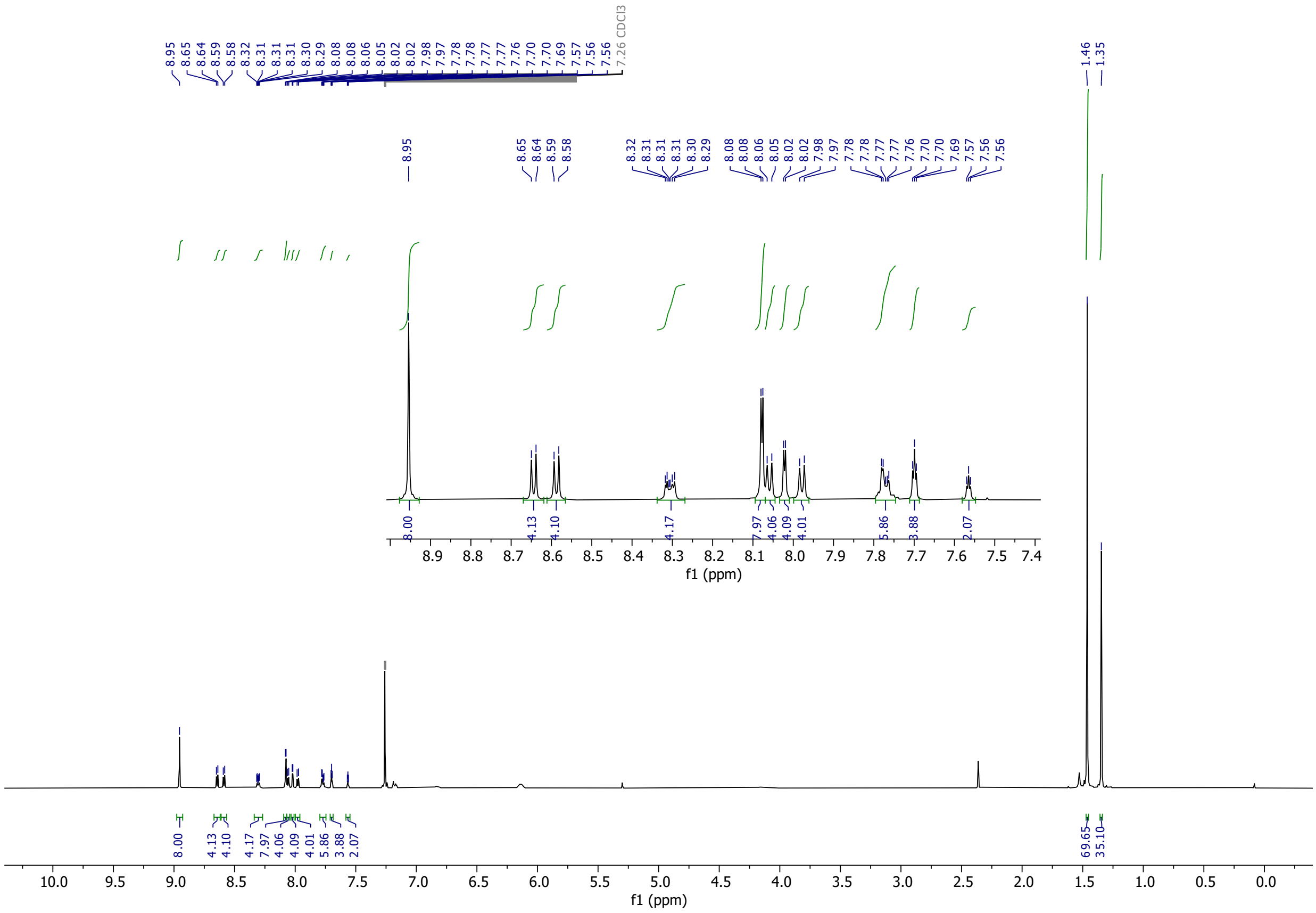

Figure S1. ^{1}H NMR spectrum of ***sl*-P3·Zn** in $CDCl_3$ (400 MHz, 298 K).

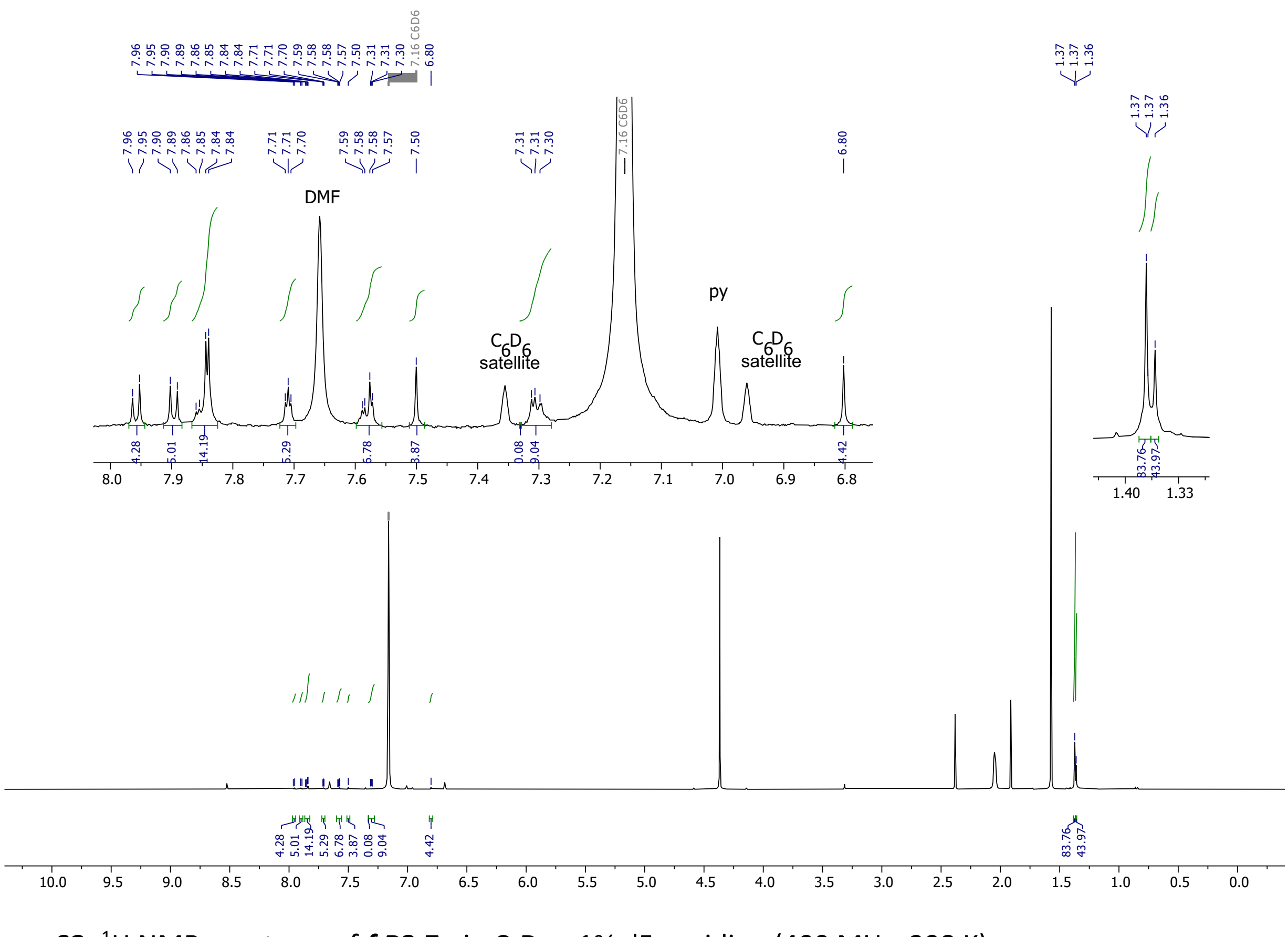

Figure S2. ^{1}H NMR spectrum of ***f*-P3·Zn** in C_6D_6 + 1% d5-pyridine (400 MHz, 298 K).

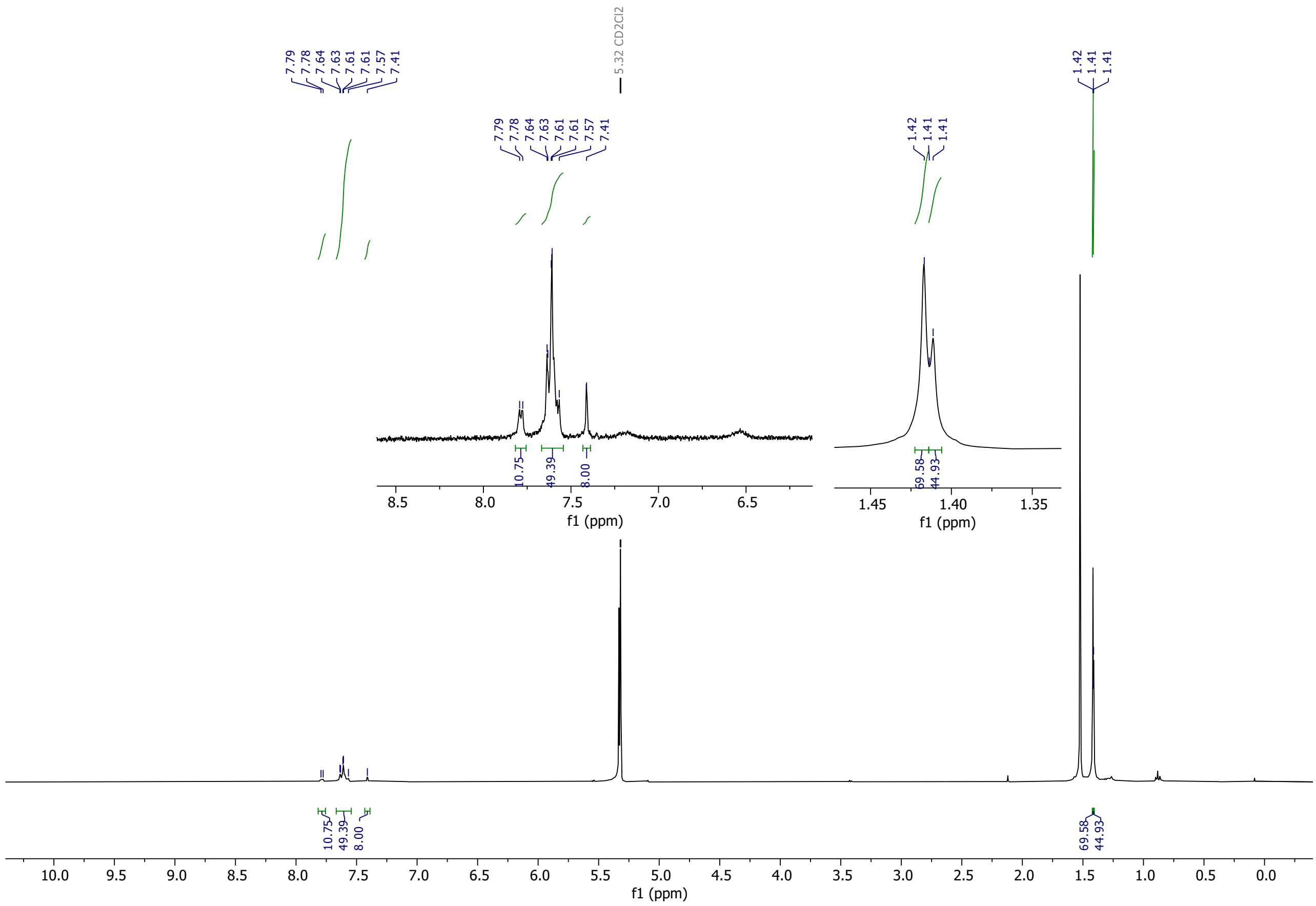

Figure S3. ^{1}H NMR spectrum of ***f*-P3·H₂** in CD_2Cl_2 (400 MHz, 298 K).

3. Paramagnetic NMR Spectra

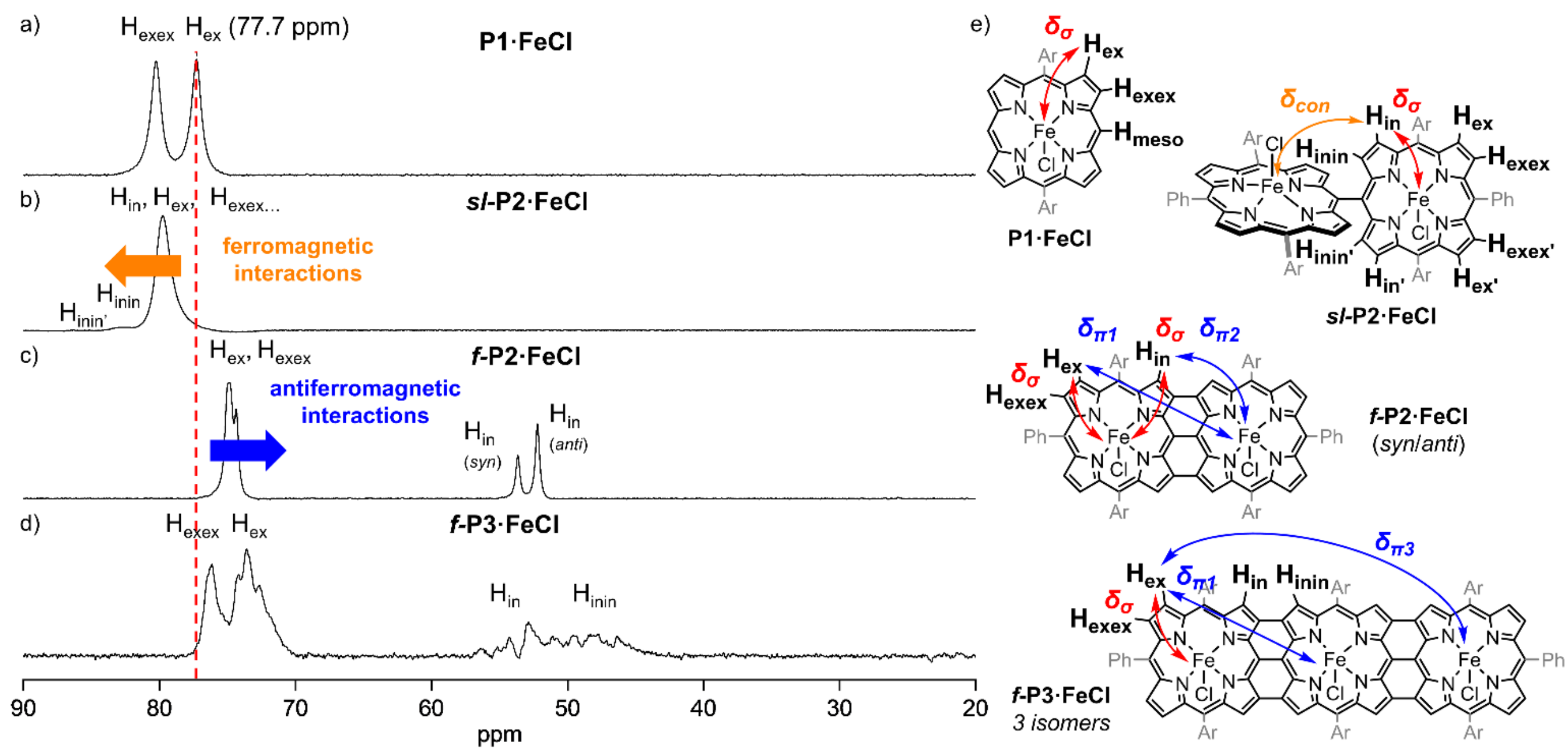

Figure S4. ^{1}H NMR spectra of a) **P1·FeCl**, b) ***sl*-P2·FeCl**, c) ***f*-P2·FeCl** and d) ***f*-P3·FeCl** ($CDCl_3$, 400 MHz, 25 °C). e) Paramagnetic contact contributions to the ^{1}H NMR chemical shift.

In this section, we proposed an experimental model based on Equation S1 (discussed in the main text) to disentangle the main contributions behind the observed chemical shifts ($\boldsymbol{\delta}$):

$$\boldsymbol{\delta} = \delta_{dia} + \delta_{para} = \delta_{dia} + \delta_{\sigma} + \delta_{\pi} + \delta_{dip} \qquad \text{(S1)}$$

In **P1·FeCl**, H_{ex} appears at $\boldsymbol{\delta}$ **= 77.7 ppm**, mainly due to the σ-mediated contact shift, as reported in the literature for the β-protons of high-spin five-coordinate Fe^{III} porphyrin monomers. The diamagnetic contribution is taken from the diamagnetic Zn^{II} analog (δ_{dia} = 9.17 ppm, Table S1). In this way, Equation S1 can be used to obtain experimental values for the paramagnetic interactions that produce the observed chemical shift, which we named δ_{σ} as it is the main contribution. The other contributions (δ_{dip} *and* δ_{π}) are considered negligible or included in δ_{σ}.

P1·FeCl, H_{ex}: $\quad \mathbf{77.7} = 9.17 + \delta_{\sigma}\ ;\ \delta_{\sigma} = \mathbf{77.7} - 9.17 = 68.53$ ppm

With this experimental value for δ_{σ}, the observed chemical shifts ($\boldsymbol{\delta}$) and the reported chemical shift of equivalent diamagnetic Zn^{II} porphyrins (δ_{dia}), it is now possible to extract additional paramagnetic contributions in the rest of the iron porphyrins. In this way, we are using H_{ex} of **P1·FeCl** as reference (vertical red line on Figs. 3 and S4), because all the Fe^{III} porphyrins studied here present protons with similar chemical environments.

In ***sl*-P2·FeCl**, the H_{inin} and $H_{inin'}$ appear at at 86.00 and 83.00 ppm, due to their proximity to the iron center of the other porphyrin, and thus, the rest of the β-protons appear at $\boldsymbol{\delta}$ **= 79.8 ppm**. The diamagnetic Zn^{II} analog, ***sl*-P2·Zn**, presents the H_{ex} at δ_{dia} = 8.95 ppm (Table S1). Adding, to Equation S1 the previously obtained value for δ_{σ} = 68.53 ppm, we can deduce an additional contribution; δ_{con}, which received this name because in it the electron spin density is plausibly transmitted by the π – σ – π orbitals.

***sl*-P2·FeCl**, H_{in}: $\ \mathbf{79.8} = 8.95 + 68.53 + \delta_{con}\ ;\ \delta_{con} = \mathbf{79.8} - 8.95 - 68.53 = 2.32$ ppm

In ***f*-P2·FeCl**, all peripheral β-protons present a similar chemical environment. The H_{in} protons appear at $\boldsymbol{\delta}$ **= 53.7 ppm** and $\boldsymbol{\delta}$ **= 52.2 ppm**, corresponding to the *syn* and *anti* isomers (discussed in the main text), and the rest of the β-protons, H_{ex} and H_{exex}, around $\boldsymbol{\delta}$ **= 74.7 ppm**. With these values ($\boldsymbol{\delta}$), the chemical shift of the equivalent protons in the diamagnetic Zn^{II} analogs (δ_{dia}, values in Table S1) and the σ-mediated contact contribution (δ_{σ}) obtained above, three new contributions are deduced. As the transmission of electron spin density beyond four σ-bonds is negligible and they produce an upfield chemical shift, we named them $\delta_{\pi 1}$ and $\delta_{\pi 2}$ (Fig. S4), assuming that the π-mediated contact contribution is the most relevant.

***f*-P2·FeCl**, H_{ex}:

$$\mathbf{74.7} = 7.44 + 68.53 + \delta_{\pi 1}\ ;\ \delta_{\pi 1} = \mathbf{74.7} - 7.44 - 68.53 = -1.27 \text{ ppm}$$

***f*-P2·FeCl**, H_{in}:

$$\mathbf{53.7} = 7.28 + 68.53 + \delta_{\pi 2}\ ;\ \delta_{\pi 2} = \mathbf{53.7} - 7.28 - 68.53 = -22.11 \text{ ppm for } \textit{syn} \text{ isomer}$$
$$\mathbf{52.2} = 7.28 + 68.53 + \delta_{\pi 2}\ ;\ \delta_{\pi 2} = \mathbf{52.2} - 7.28 - 68.53 = -23.61 \text{ ppm for } \textit{anti} \text{ isomer}$$

In ***f*-P3·FeCl**, the use of the experimental contributions obtained above, the average of the experimental $\boldsymbol{\delta}$ values (H_{ex}, $\boldsymbol{\delta}$ **= 72.8 to 74.2 ppm**; H_{in}, $\boldsymbol{\delta}$ **= 51.0 to 56.8 ppm**; H_{inin}, $\boldsymbol{\delta}$ **= 46.4 to 51.0 ppm**), and the diamagnetic contribution from the analog Zn^{II} porphyrin trimer (H_{ex}, δ_{dia} = 7.52 ppm; H_{in}, δ_{dia} = 6.97 ppm; H_{inin}, δ_{dia} = 6.23 ppm) allow to obtain an experimental value for the distal contribution $\delta_{\pi 3}$.

***f*-P3·FeCl**, H_{ex}:

$$\mathbf{73.5} = 7.44 + 68.53 + \delta_{\pi 1} + \delta_{\pi 3}\ ;\ \delta_{\pi 3} = \mathbf{73.5} - 7.44 - 68.53 - (-1.27) = -1.17 \text{ ppm}$$

Moreover, the usage of the experimentally obtained contributions in the monomer and dimers, allows to predict the presence of H signals below 52 ppm in ***f*-P3·FeCl**:

***f*-P3·FeCl**, H_{in}:

$$\boldsymbol{\delta} = \delta_{dia} + \delta_{\sigma} + \delta_{\pi 2} + \delta_{\pi 1}$$
$$\boldsymbol{\delta} = 7.44 + 68.53 + (-23.61 \text{ to } -22.11) + (-1.27) = 51.09 \text{ to } 52.56 \text{ ppm (Exp. value 46.4 – 56.8 ppm)}$$

Table S1 collects the experimental values for the different contributions:

Table S1. **^{1}H NMR chemical shift contributions from individual Fe(III)Cl centers.**

<table>
<tr><th rowspan="3">compound / δ (ppm)</th><th rowspan="3">H</th><th rowspan="3">δ_{dia}</th><th colspan="5">δ_{para}</th><th rowspan="3">δ_{exp}</th></tr>
<tr><th rowspan="2">δ_{dip}</th><th colspan="4">δ_{con}</th></tr>
<tr><th>δ_{σ}</th><th>$\delta_{\pi 1}$</th><th>$\delta_{\pi 2}$</th><th>$\delta_{\pi 3}$</th></tr>
<tr><td>P1·Fe</td><td>H_{ex}</td><td>9.17</td><td rowspan="7">0</td><td rowspan="7">68.5</td><td colspan="3">-</td><td>77.7</td></tr>
<tr><td>sl-P2·Fe</td><td>H_{in}</td><td>8.95</td><td colspan="3">2.32</td><td>79.8</td></tr>
<tr><td rowspan="2">syn-P2·Fe</td><td>H_{ex}</td><td>7.44</td><td>-1.27</td><td colspan="2">-</td><td>74.7</td></tr>
<tr><td>H_{in}</td><td>7.28</td><td>-</td><td>-22.11</td><td>-</td><td>53.7</td></tr>
<tr><td rowspan="2">anti-P2·Fe</td><td>H_{ex}</td><td>7.44</td><td>-1.27</td><td colspan="2">-</td><td>74.7</td></tr>
<tr><td>H_{in}</td><td>7.28</td><td>-</td><td>-23.61</td><td>-</td><td>52.2</td></tr>
<tr><td>f-P3·Fe</td><td>H_{ex}</td><td>7.52</td><td>-1.27</td><td>-</td><td>-1.17</td><td>73.5</td></tr>
</table>

These contributions ($\delta_{dia} + \delta_{\sigma} + \delta_{\pi 1} + \delta_{\pi 2} + \delta_{\pi 3}$) are proportional to the hyperfine couplings between the iron centers and the corresponding protons, according to equation (2),

$$\delta = \frac{A_{\mathrm{H}} g \mu_B S(S+1)}{3\gamma_{\mathrm{N}} \hbar k_B T} \qquad \text{(S2)}$$

where *S* is the total electron spin quantum number, γ_N is the magnetogyric ratio of the nucleus, *T* is the temperature and A_H the hyperfine coupling.

The spin polarization of diamagnetic orbitals is the propagation channel of hyperfine (A_H) and exchange (*J*) couplings. The sign of these magnetic interactions depends on the orbitals involved in their transmission. This phenomenon is evident in the ^{1}H NMR spectra of the iron porphyrins studied (Fig. S4). The analysis of their individual paramagnetic contributions to the chemical shift (and associated hyperfine couplings), reflects the sign of the exchange coupling between iron centers.

In ***sl*-P2·FeCl**, in which the exchange coupling between iron centers is ferromagnetic, the equivalent β-proton signals (H_{in}, H_{ex}, $H_{exex,}$ $H_{in'}$ $H_{ex'}$ and $H_{exex'}$) appear downfield shifted relative to H_{ex} in **P1·FeCl**. Therefore, we concluded that the hyperfine couplings (A_H) between each iron center and the protons on the remote porphyrin unit, as well as the exchange coupling (*J*) between the iron centers are positive (*J* and $A_H > 0$, ferromagnetic). This occurs because the π-electron spin density from the remote iron center is transmitted through a σ-bond to the π-system of the adjacent porphyrin unit, resulting in a sign inversion relative to the π-electron spin density within each porphyrin.

In contrast, the rest of the compounds exhibit antiferromagnetic exchange coupling ($J < 0$), and their equivalent β-protons (***f*-P2·FeCl**: H_{in} and H_{ex}; and ***f*-P3·FeCl**: H_{in}, H_{ex} and H_{exex}) are shifted upfield relative to H_{ex} in **P1·FeCl** (Fig. S4). This also indicates negative hyperfine couplings ($A_H < 0$) between each iron center and the protons on the other porphyrin units.

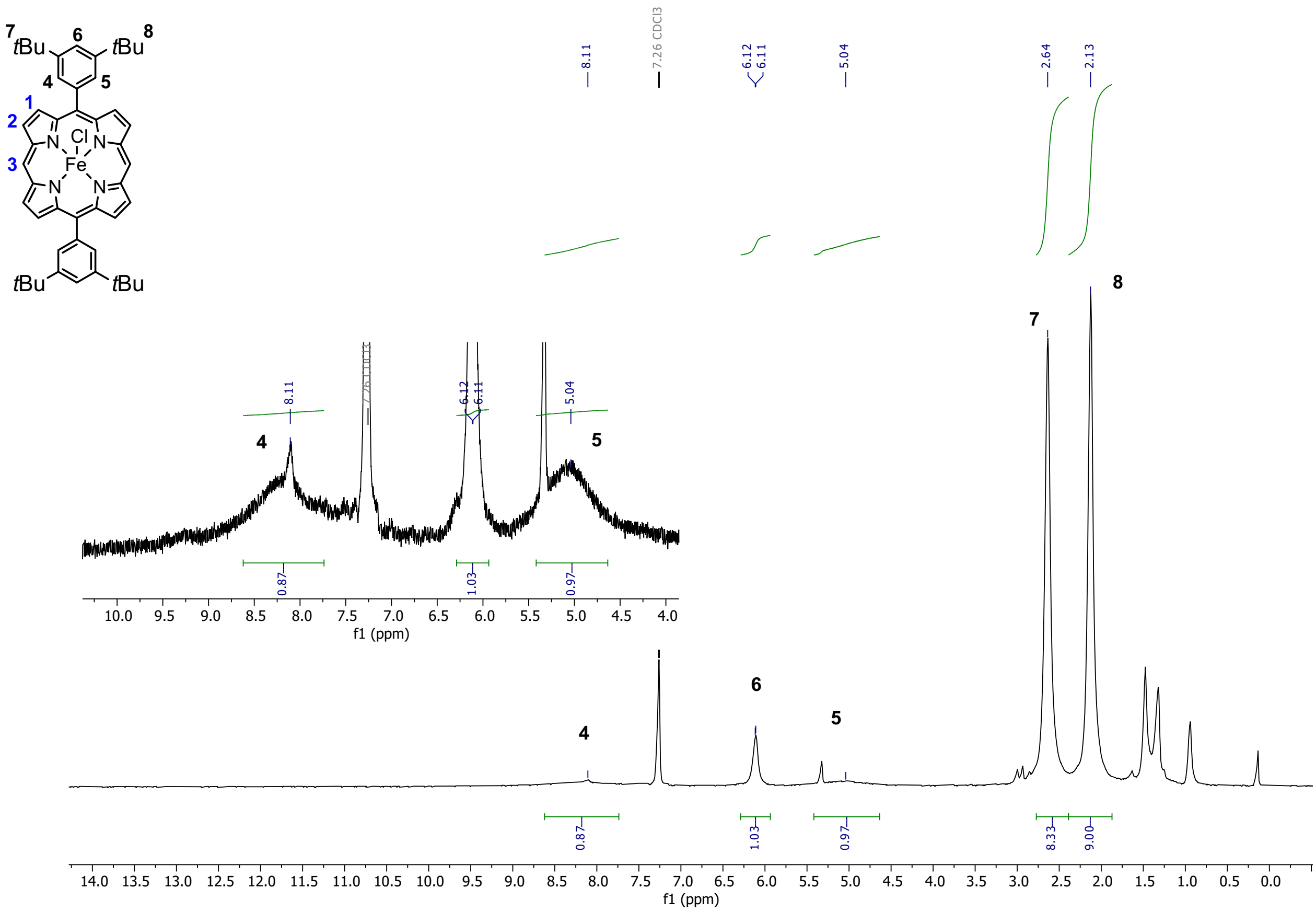

Figure S5. ^{1}H NMR spectrum of **P1·FeCl** in $CDCl_3$ (400 MHz, 298 K, narrow window).

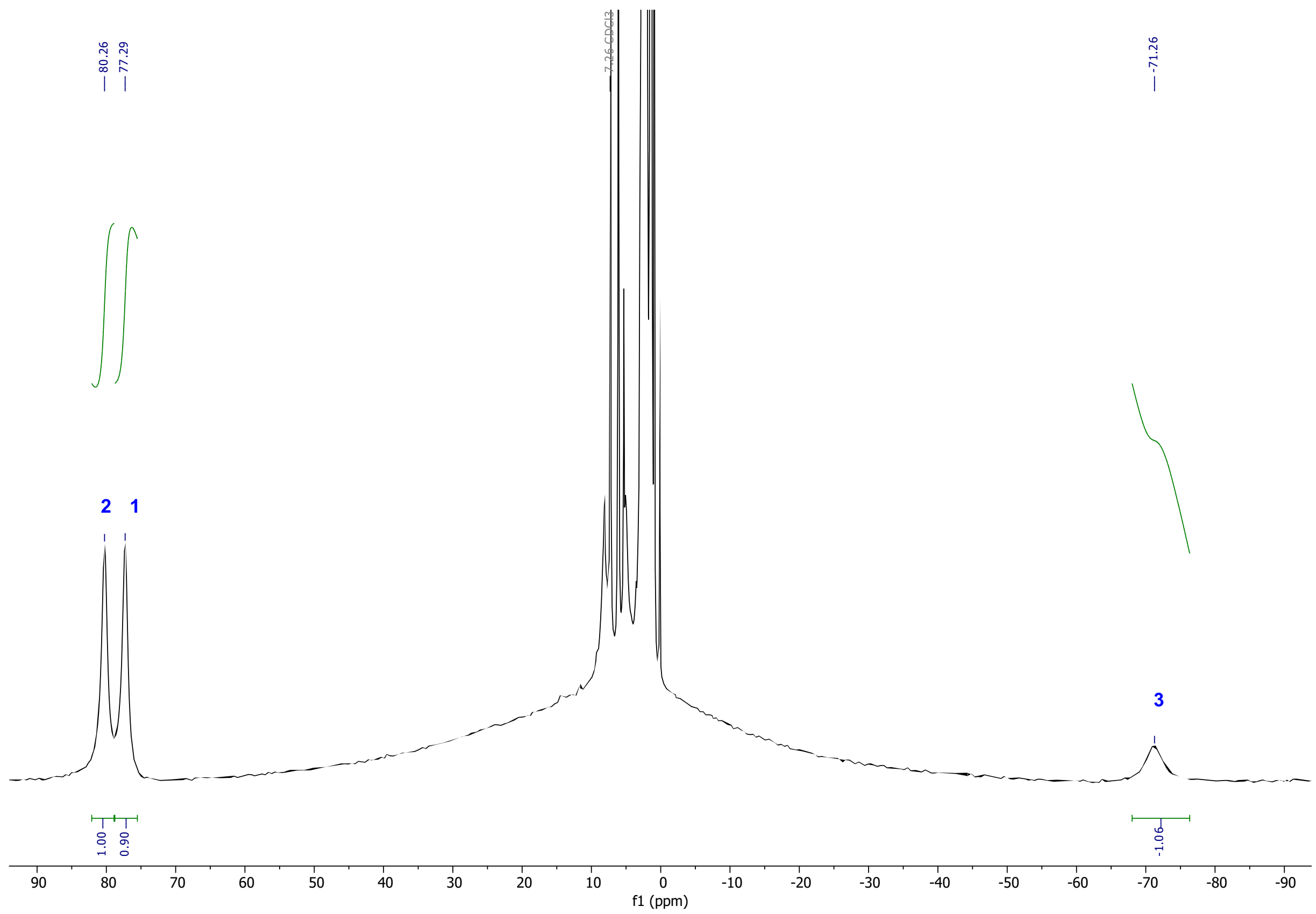

Figure S6. ^{1}H NMR spectrum of **P1·FeCl** in $CDCl_3$ (400 MHz, 298 K, wide window).

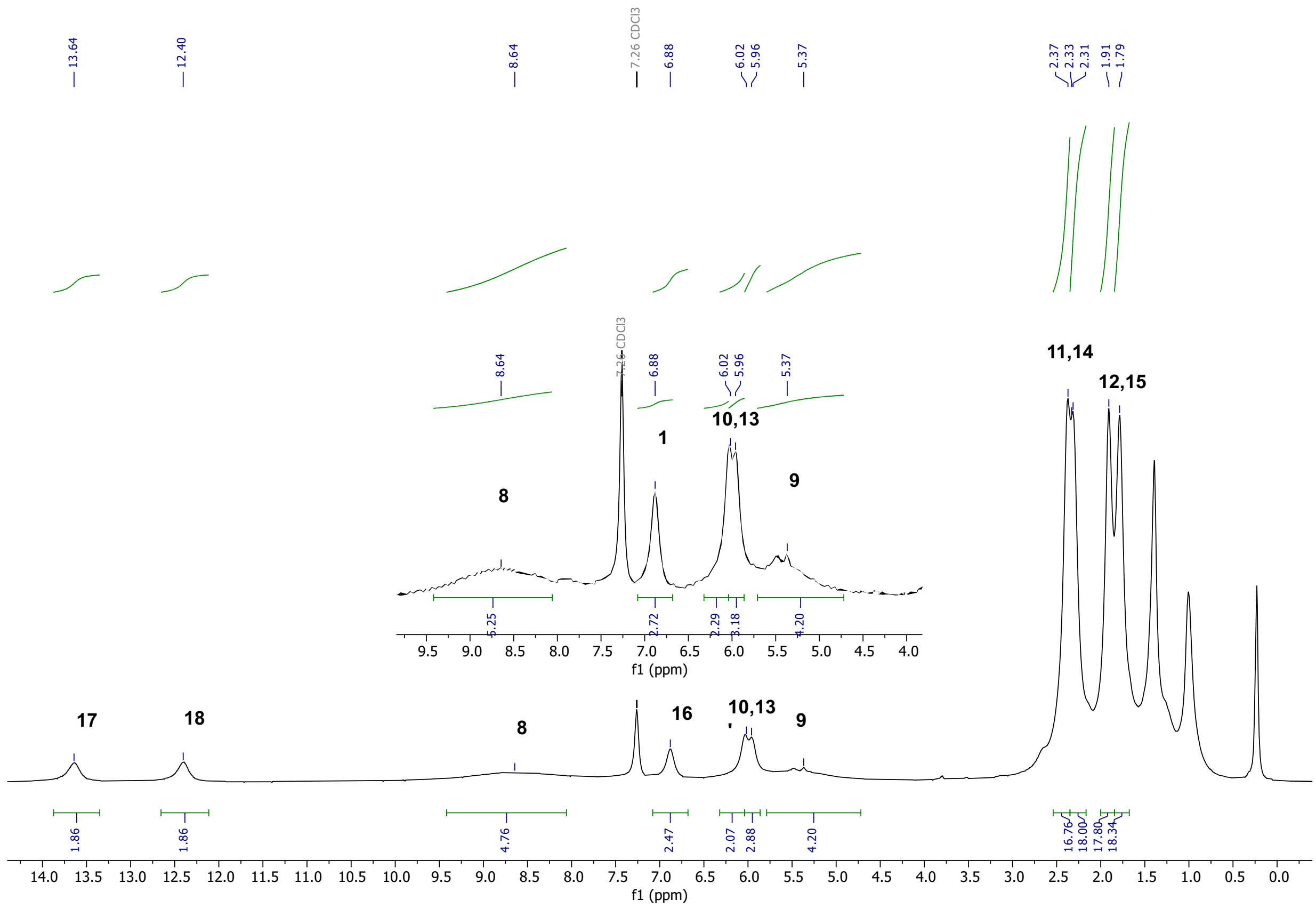

Figure S7. ^{1}H NMR spectrum of ***sl*-P2·FeCl** in $CDCl_3$ (400 MHz, 298 K, narrow window).

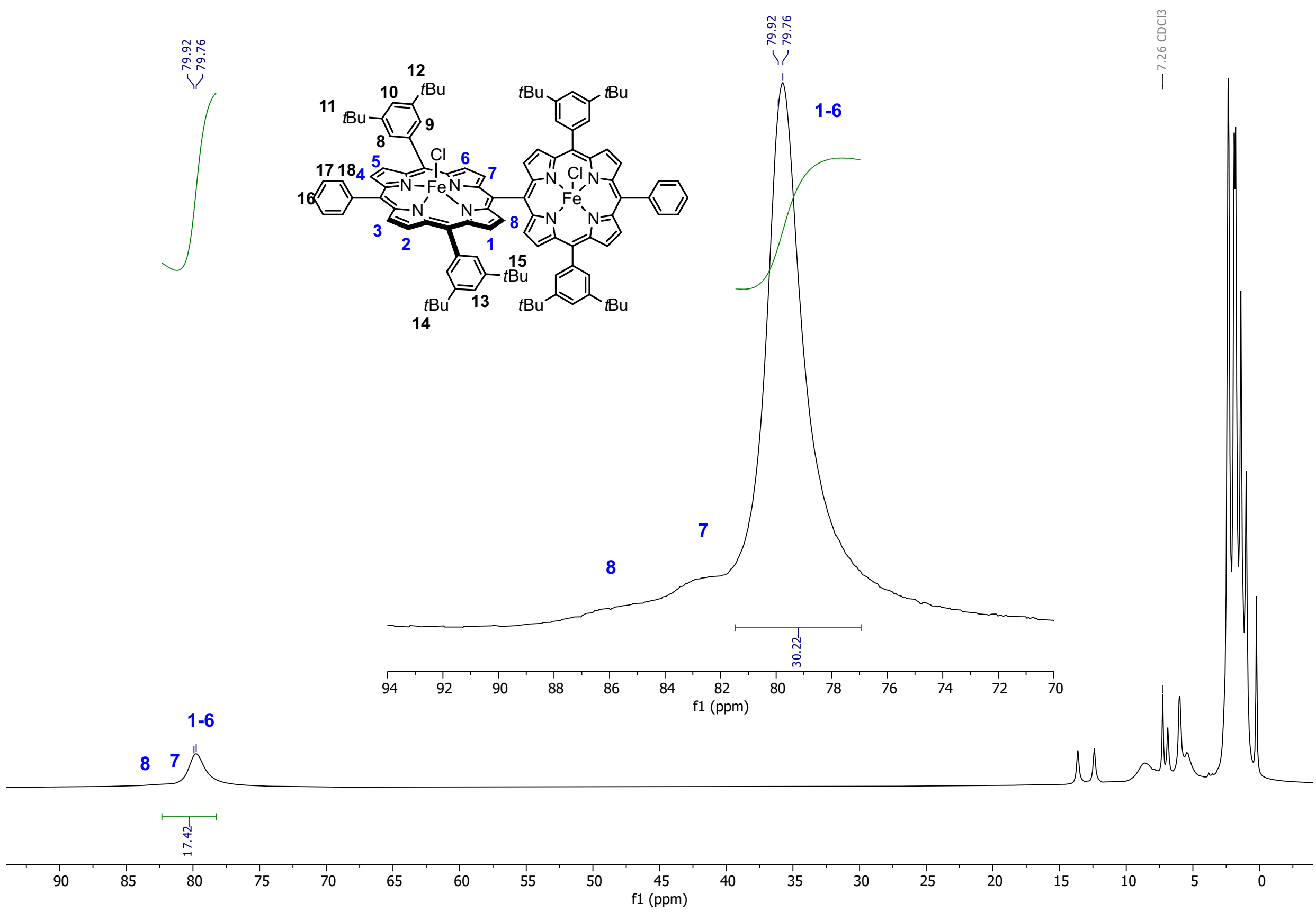

Figure S8. ^{1}H NMR spectrum of ***sl*-P2·FeCl** in $CDCl_3$ (400 MHz, 298 K, wide window).

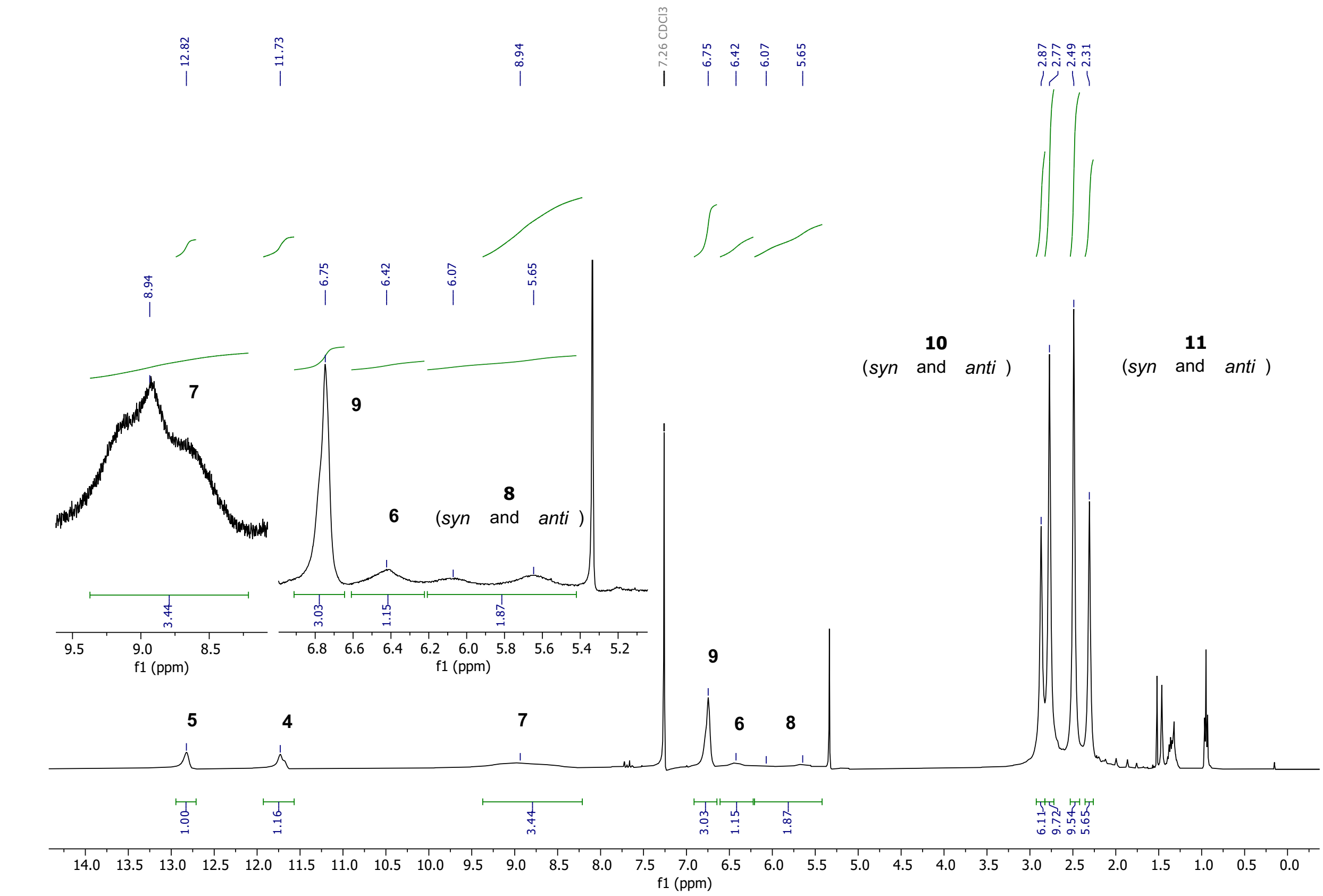

Figure S9. ^{1}H NMR spectrum of ***f*-P2·FeCl** in $CDCl_3$ (400 MHz, 298 K, narrow window).

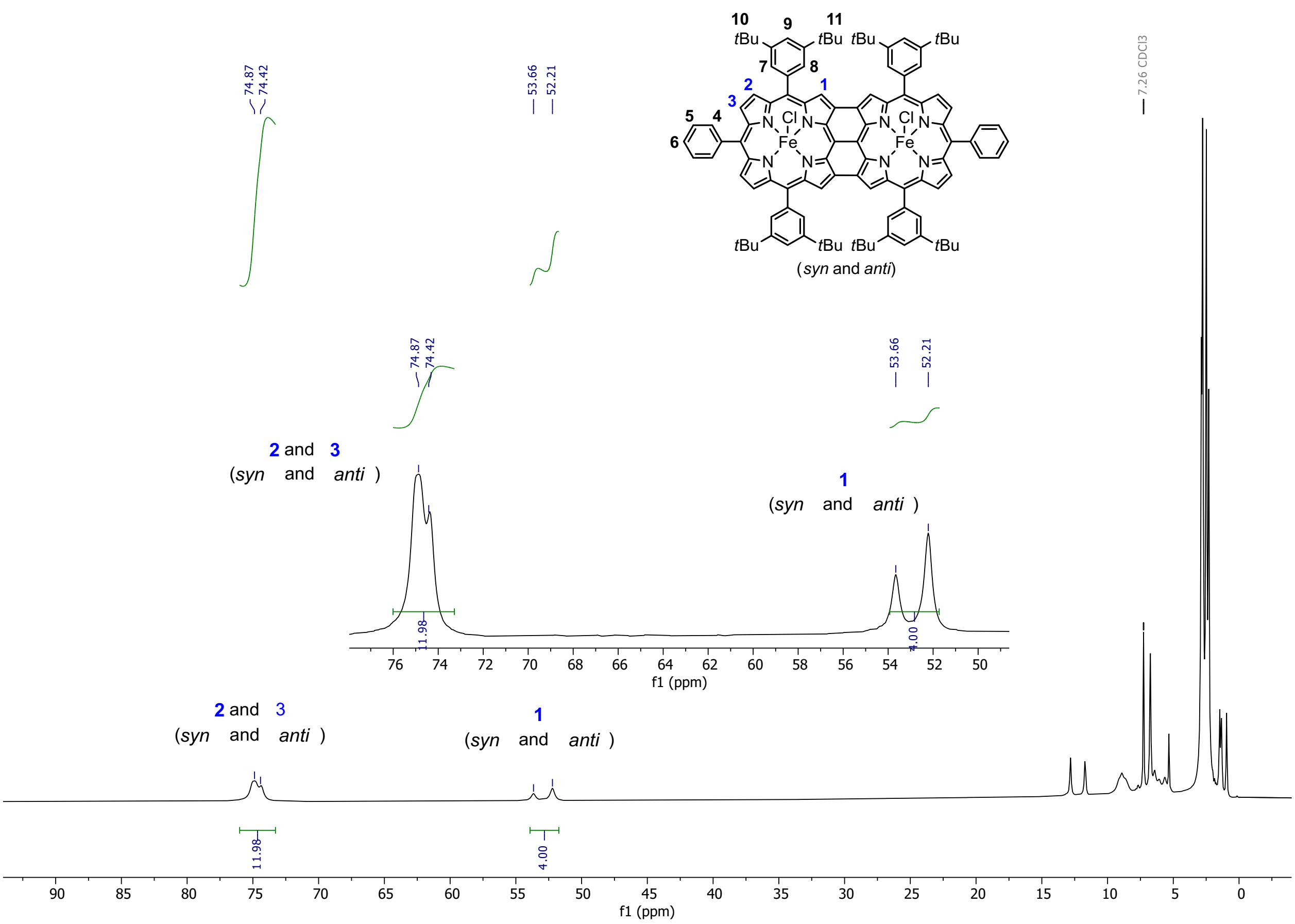

Figure S10. ^{1}H NMR spectrum of ***f*-P2·FeCl** in $CDCl_3$ (400 MHz, 298 K, wide window).

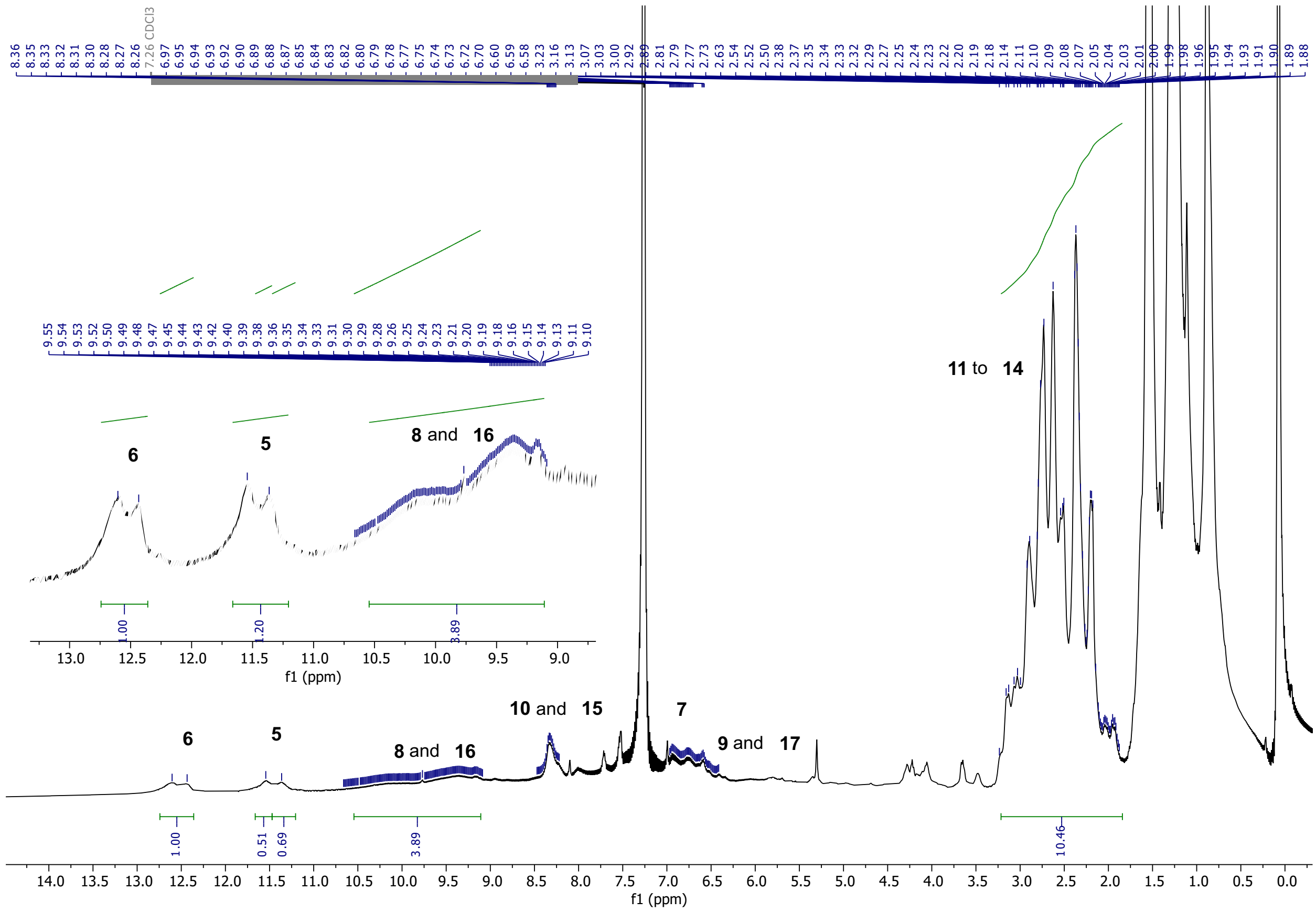

Figure S11. ^{1}H NMR spectrum of ***f*-P3·FeCl** in $CDCl_3$ (400 MHz, 298 K, narrow window).

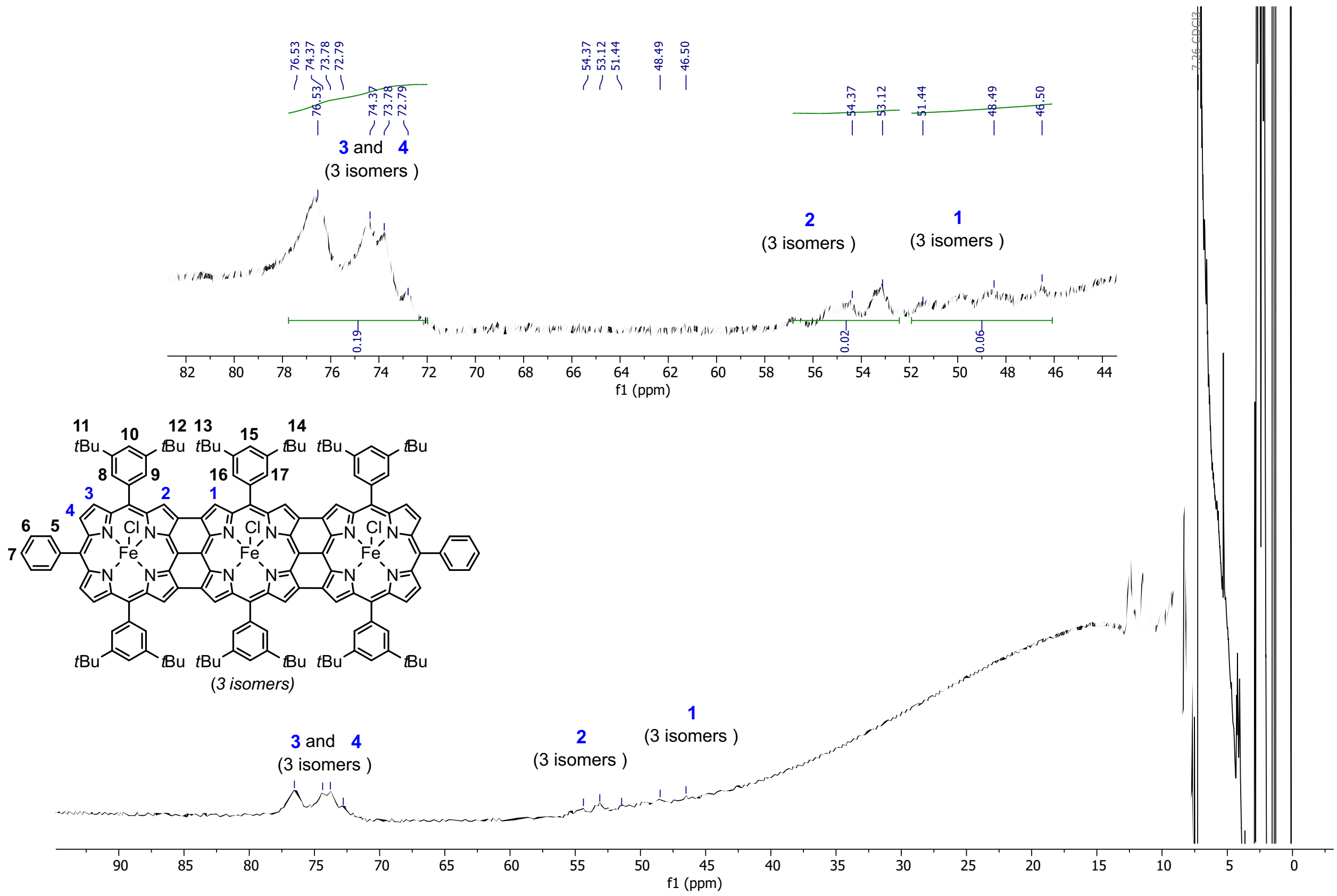

Figure S12. ^{1}H NMR spectrum of ***f*-P3·FeCl** in $CDCl_3$ (400 MHz, 298 K, wide window).

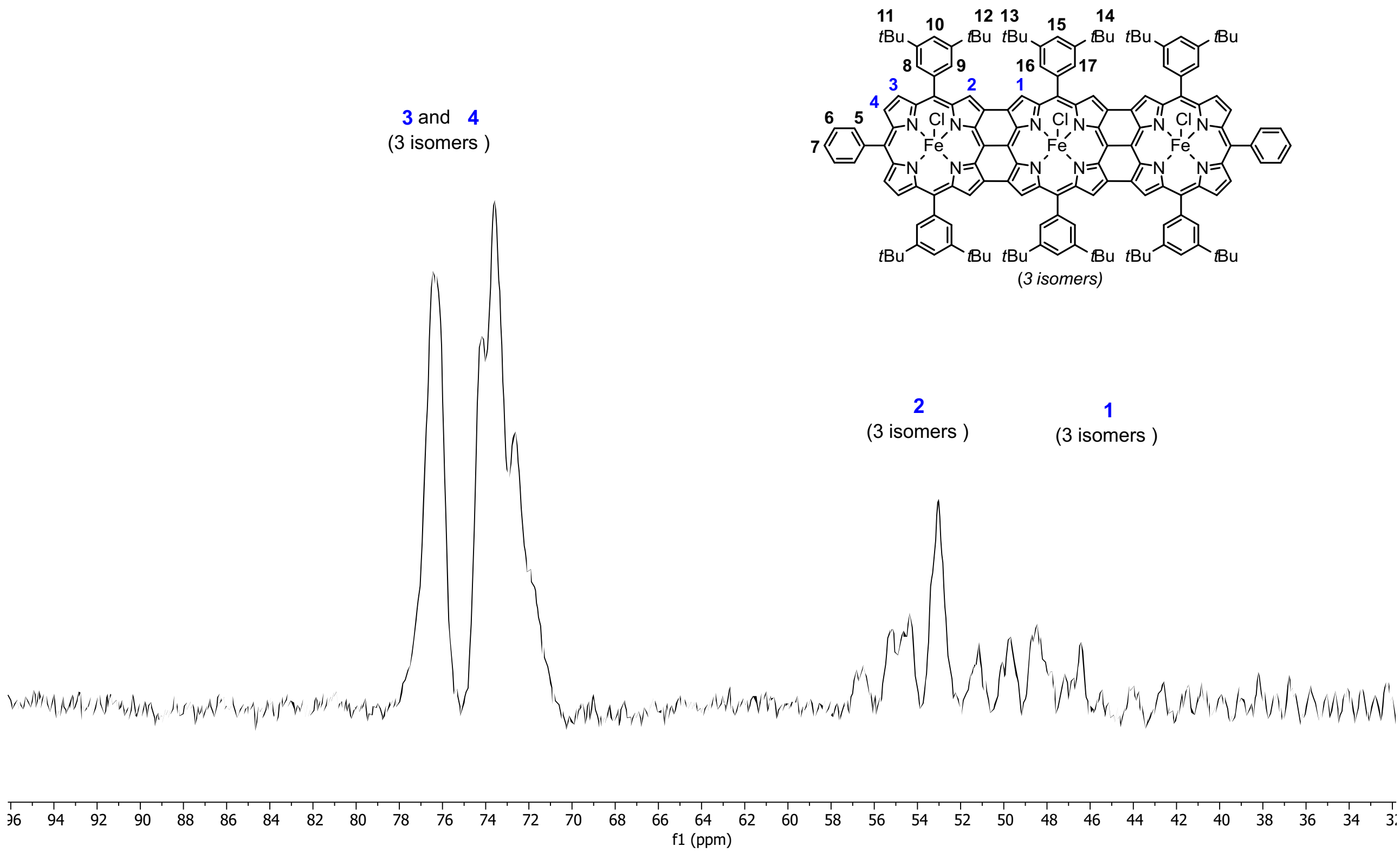

Figure S13. ^{1}H NMR spectrum of ***f*-P3·FeCl** in $CDCl_3$ (400 MHz, 298 K, baseline corrected).

4. UV-vis-NIR and IR Spectra

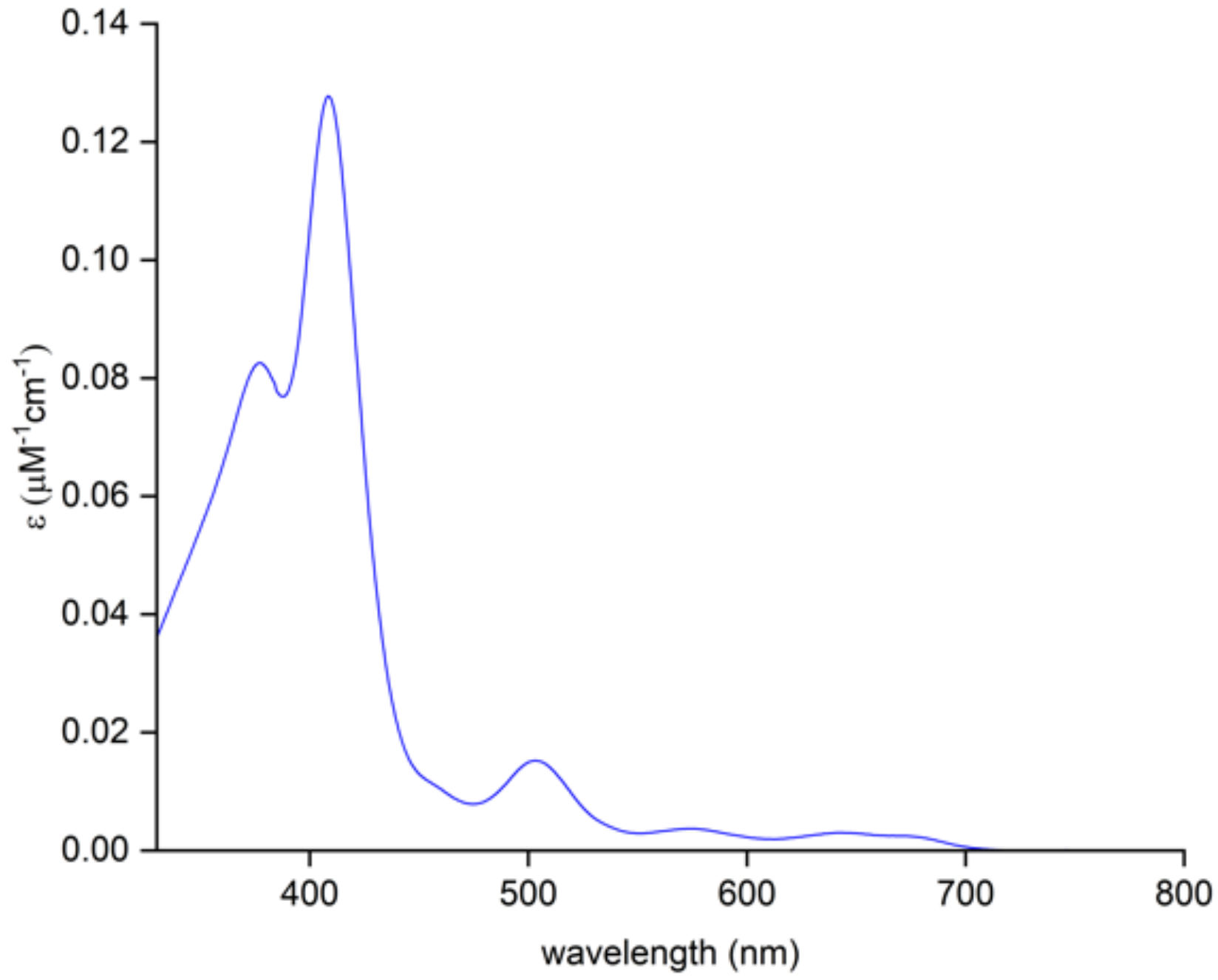

Figure S14. UV-vis-NIR spectrum of **P1·FeCl** in dichloromethane (298 K).

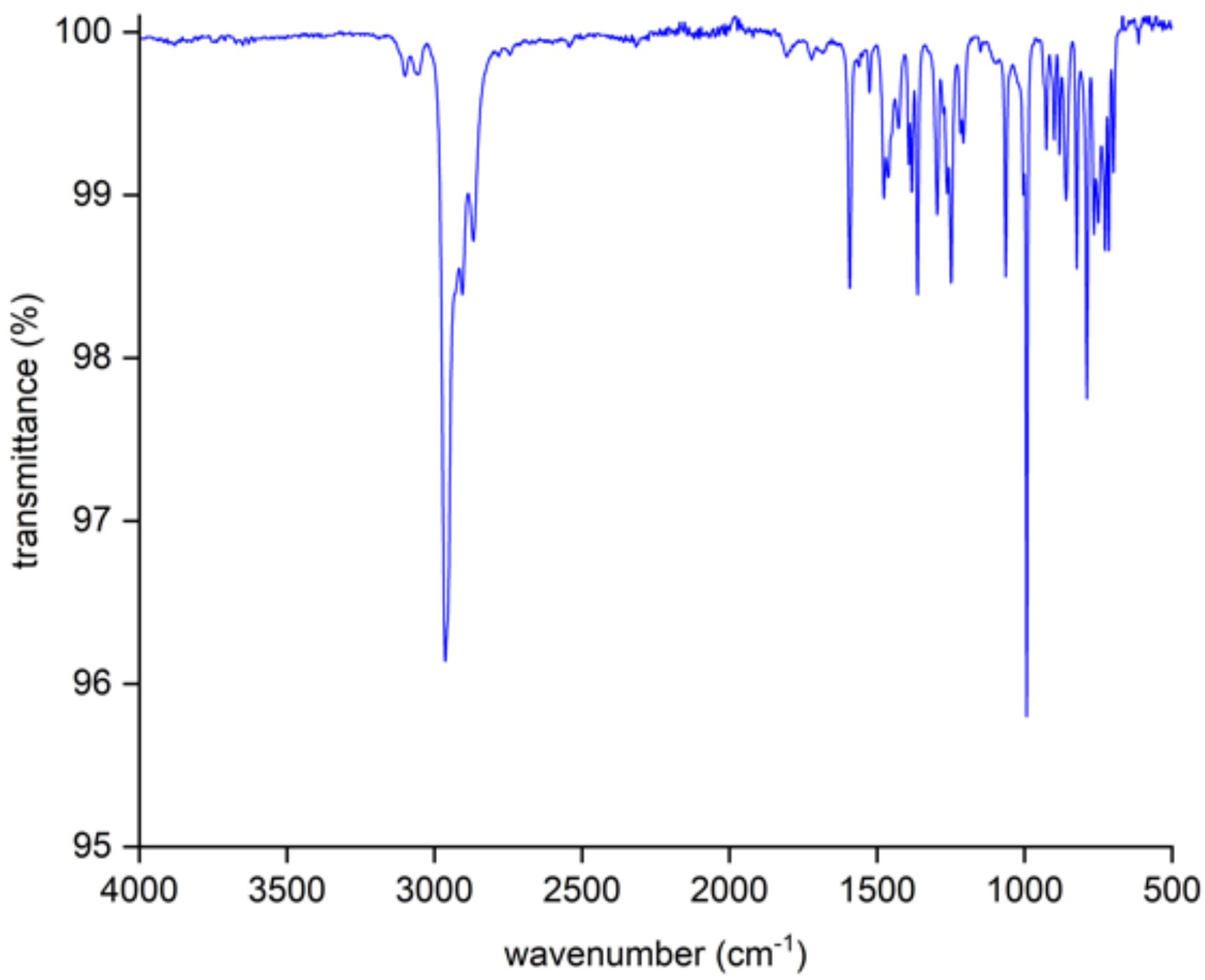

Figure S15. IR spectrum of **P1·FeCl** dry film.

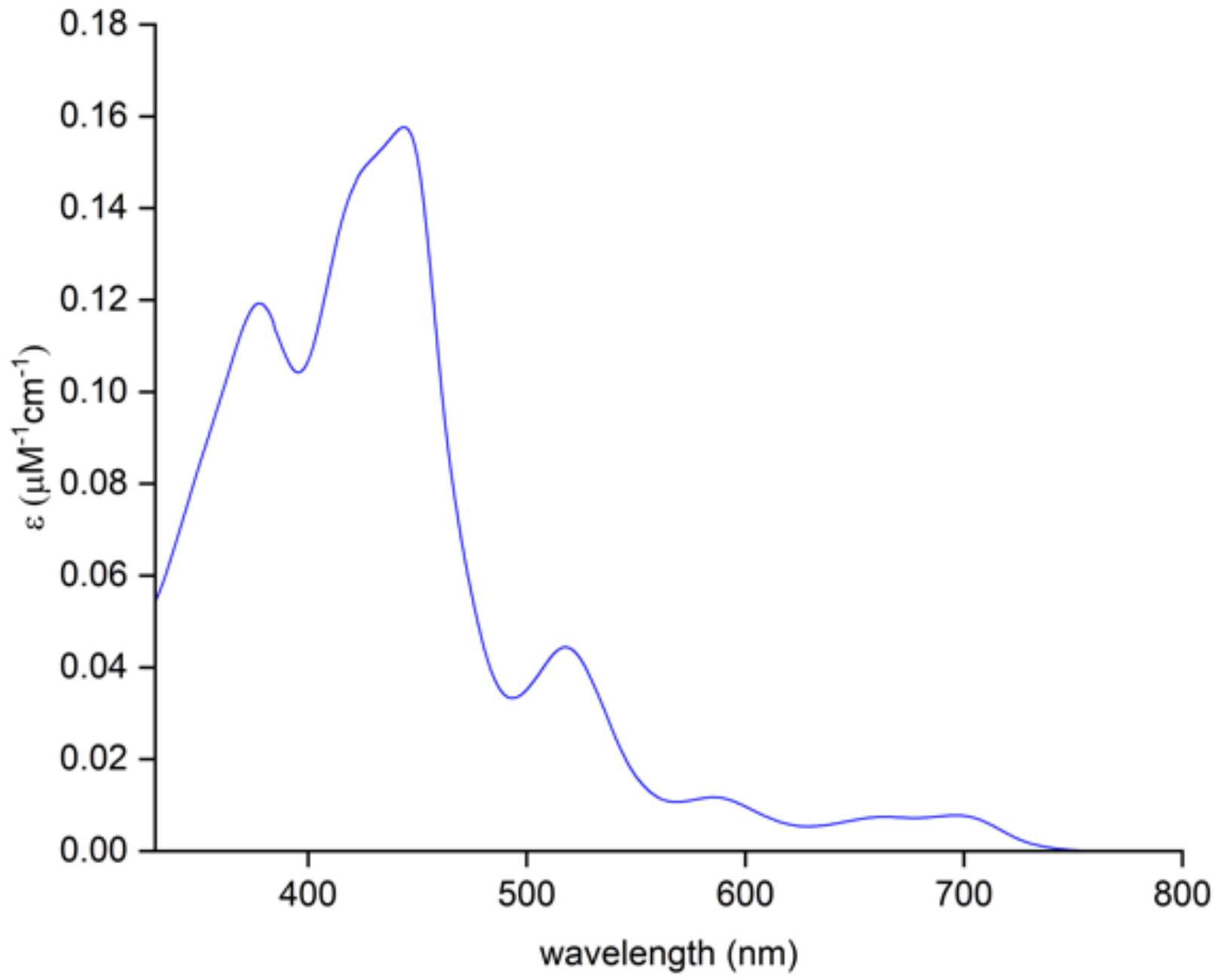

Figure S16. UV-vis-NIR spectrum of ***sl*-P2·FeCl** in dichloromethane (298 K).

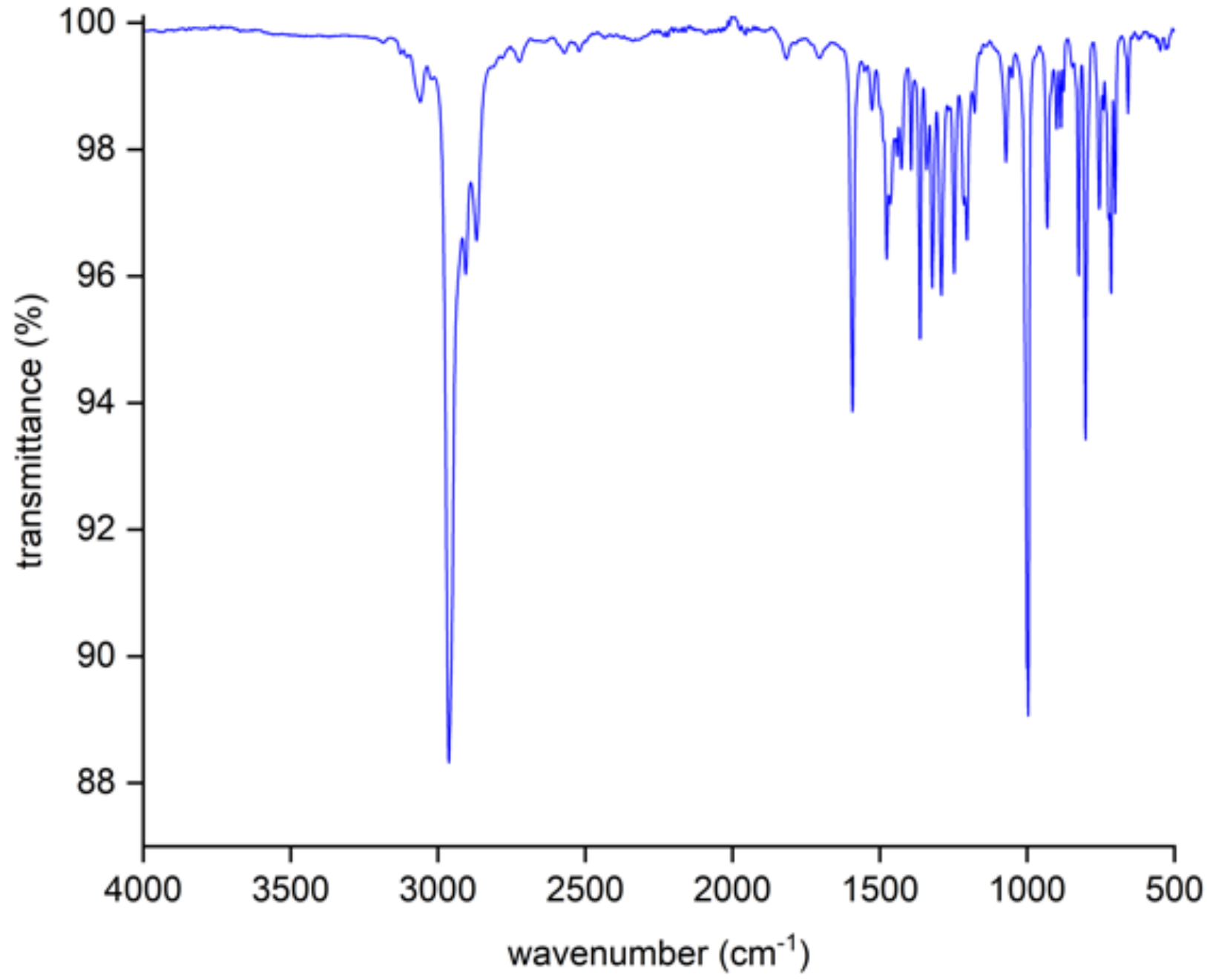

Figure S17. IR spectrum of ***sl*-P2·FeCl** dry film.

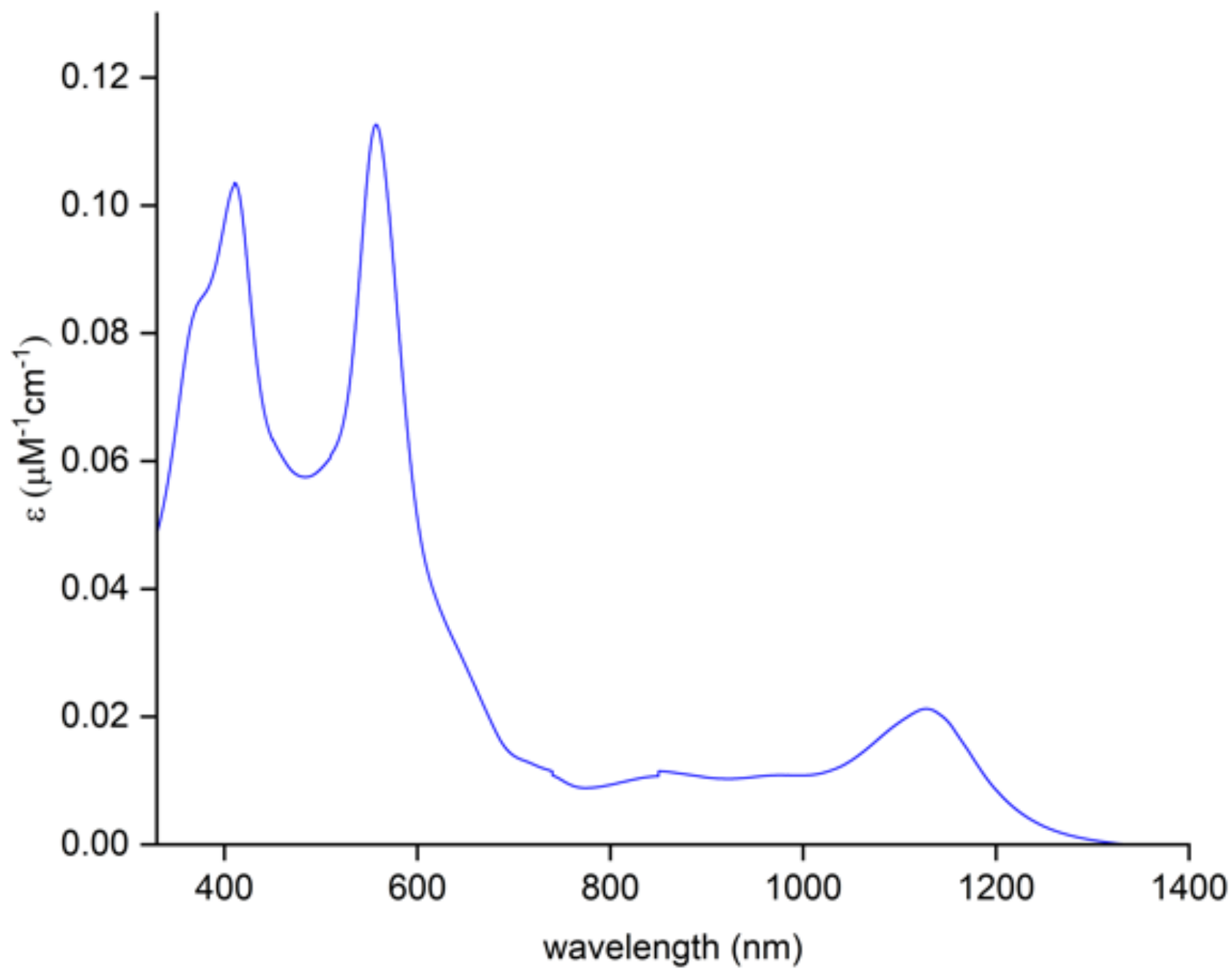

Figure S18. UV-vis-NIR spectrum of ***f*-P2·FeCl** in dichloromethane (298 K).

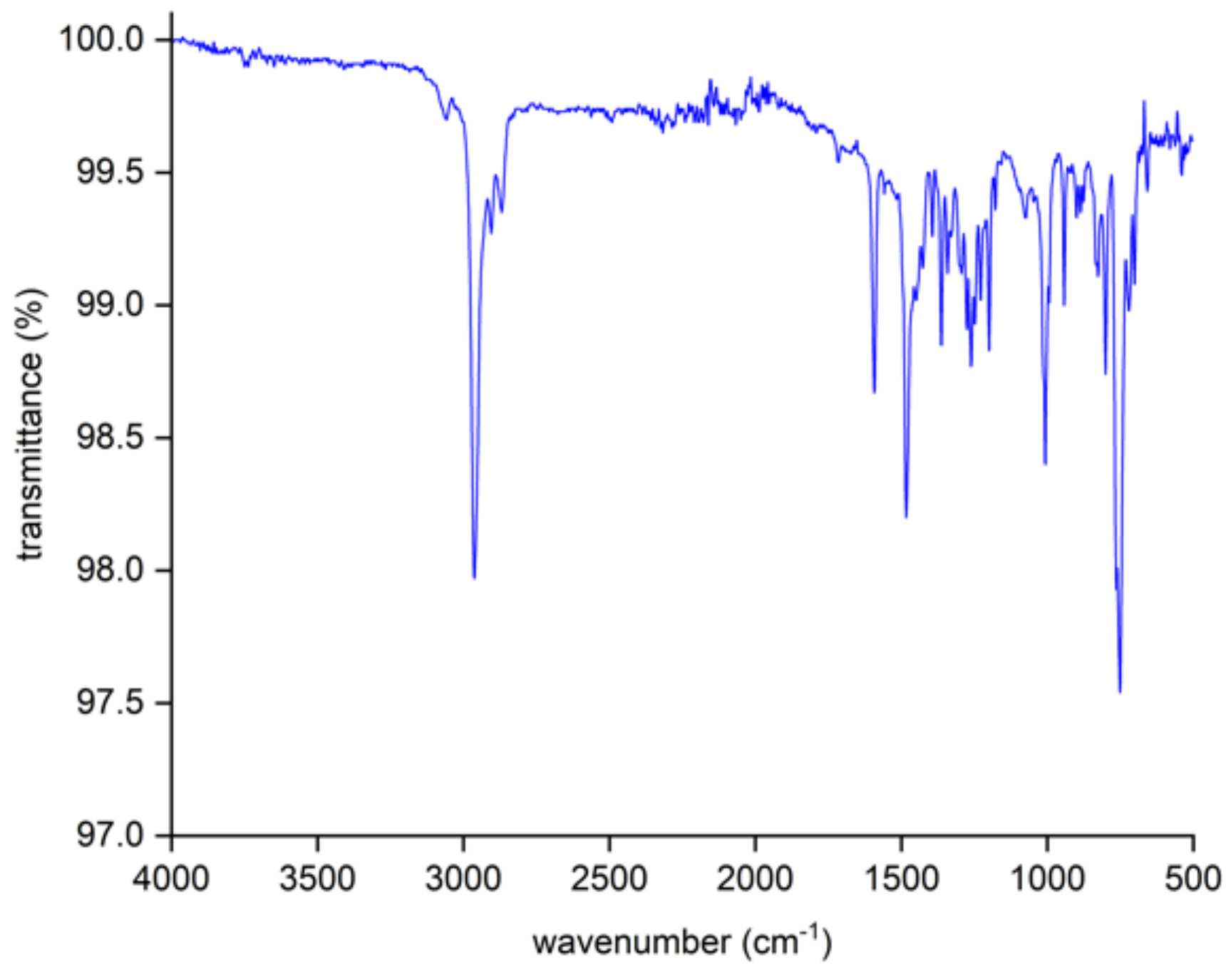

Figure S19. IR spectrum of ***f*-P2·FeCl** dry film.

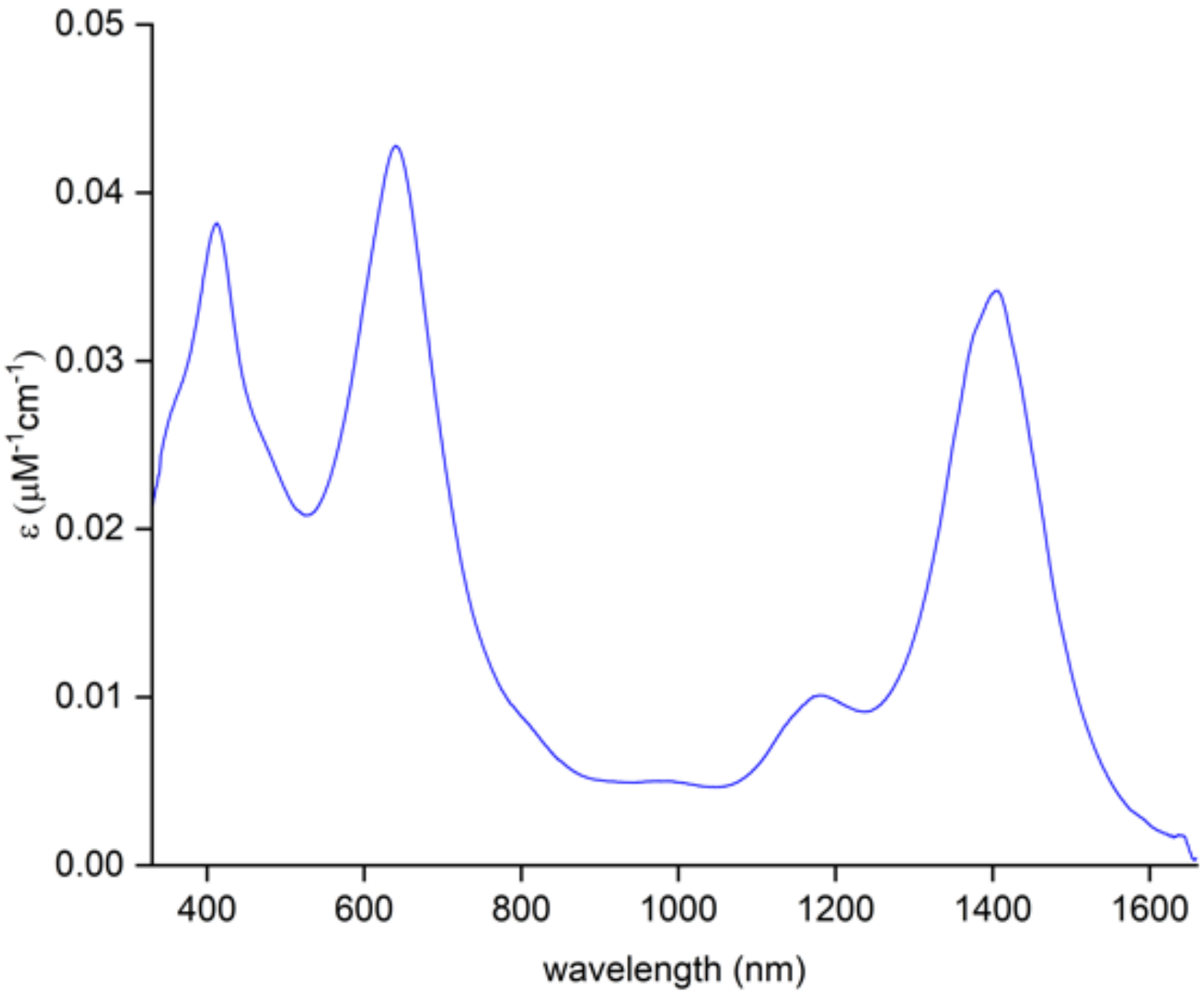

Figure S20. UV-vis-NIR spectrum of ***f*-P3·FeCl** in dichloromethane (298 K).

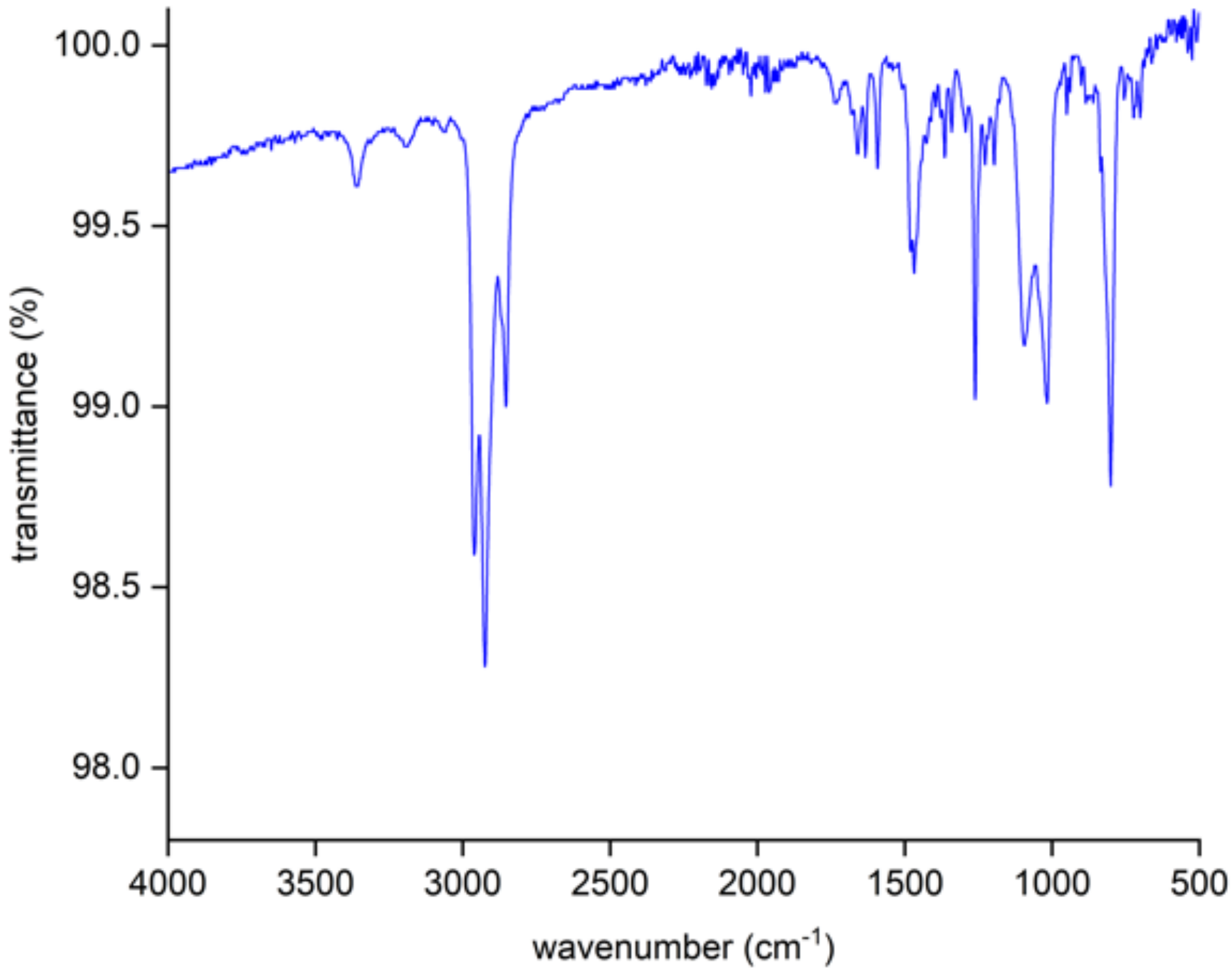

Figure S21. IR spectrum of ***f*-P3·FeCl** dry film.

5. MALDI Mass Spectra

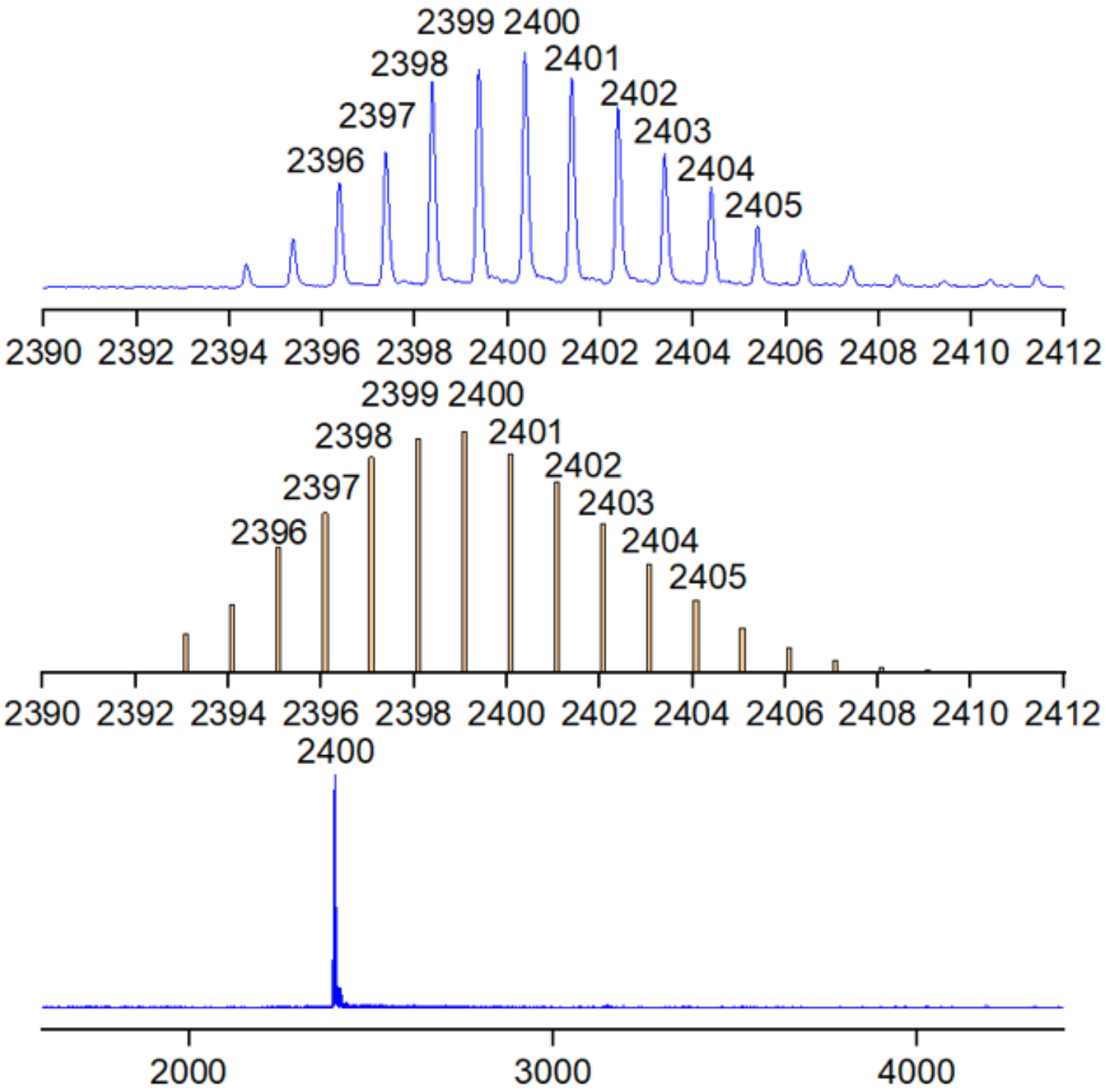

Figure S22. MALDI spectrum of ***sl*-P3·Zn** (reflection and positive mode, DCTB matrix).

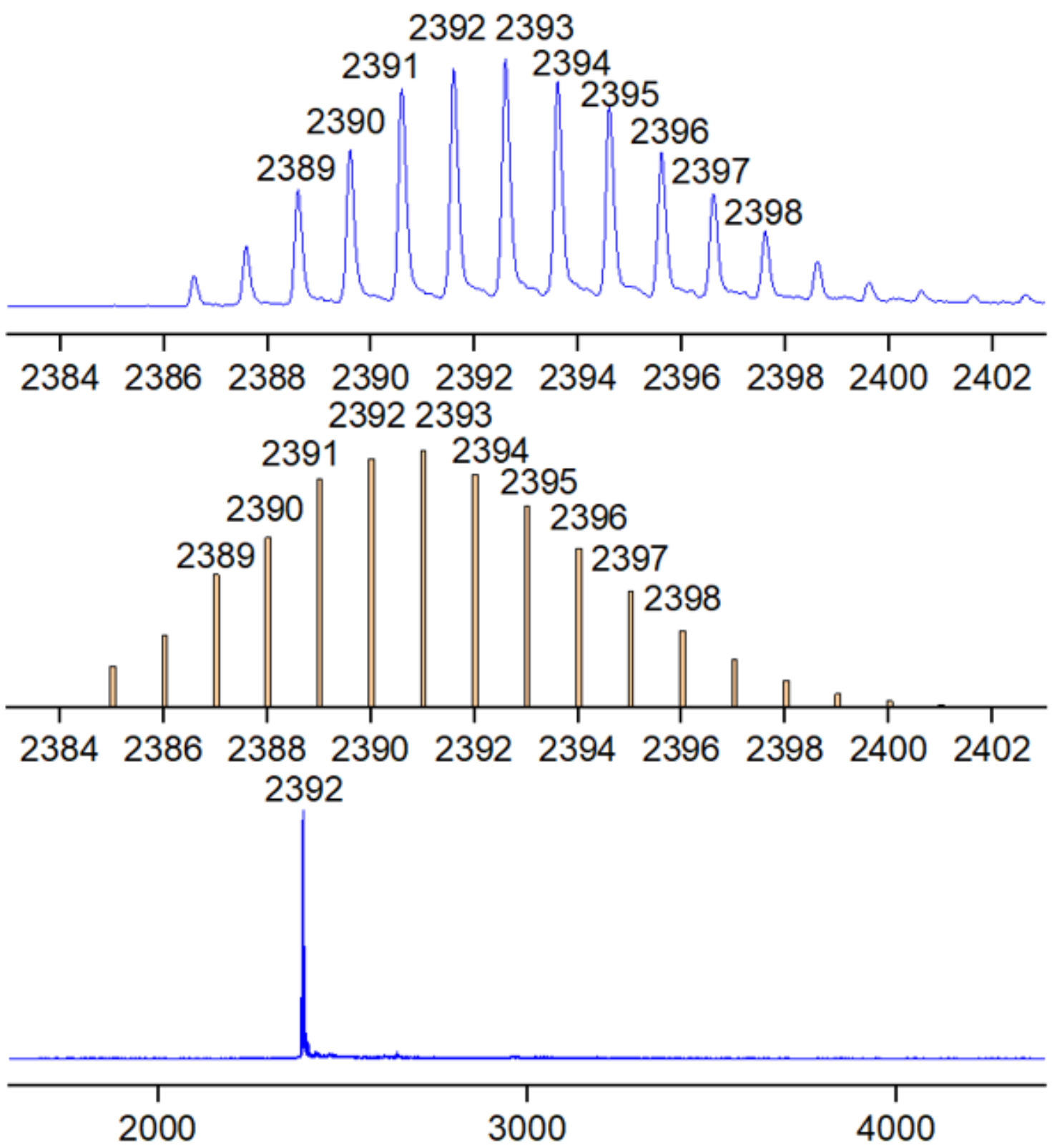

Figure S23. MALDI spectrum of ***f*-P3·Zn** (reflection and positive mode, DCTB matrix).

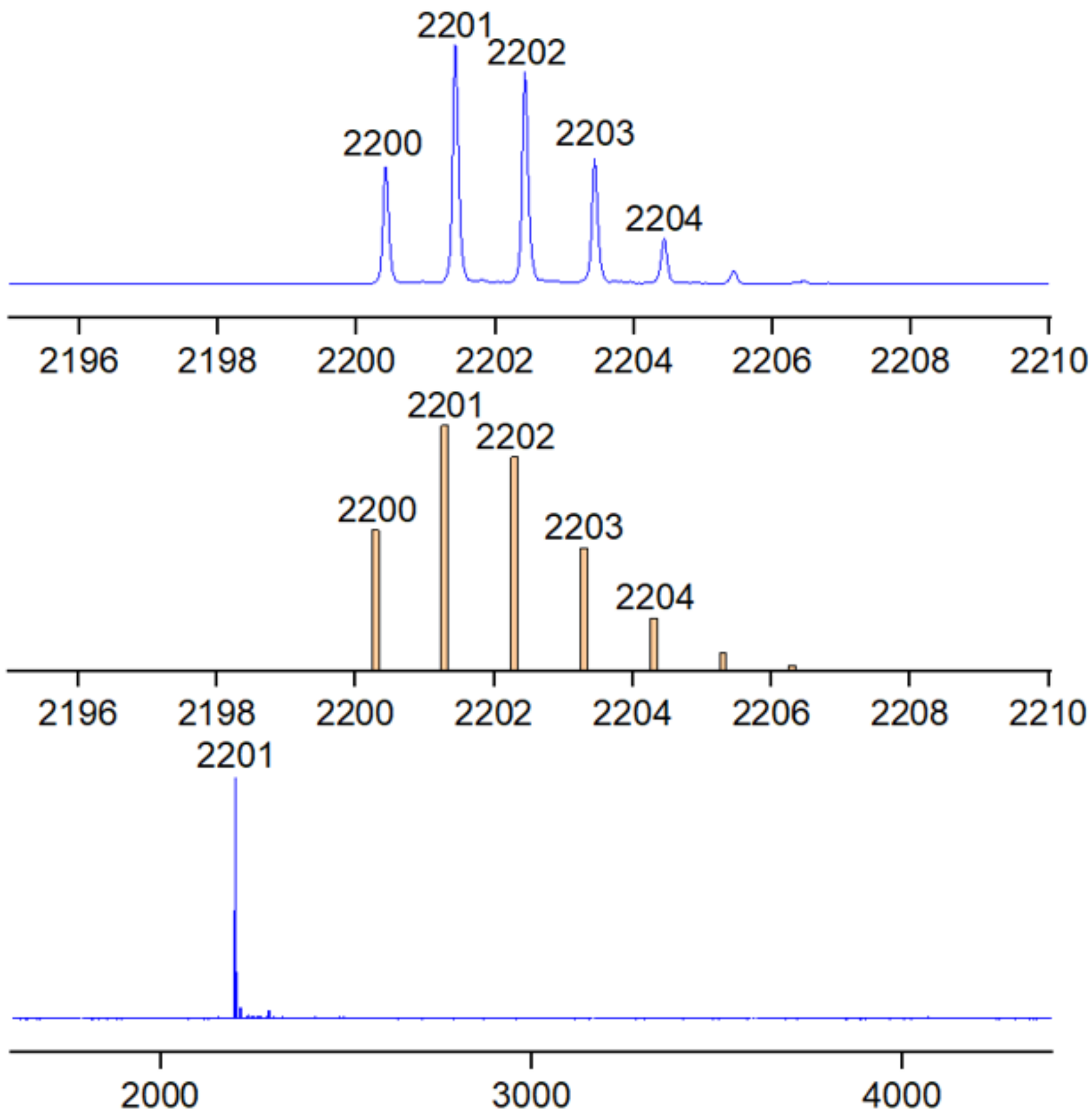

Figure S24. MALDI spectrum of ***f*-P3·H₂** (reflection and positive mode, DCTB matrix).

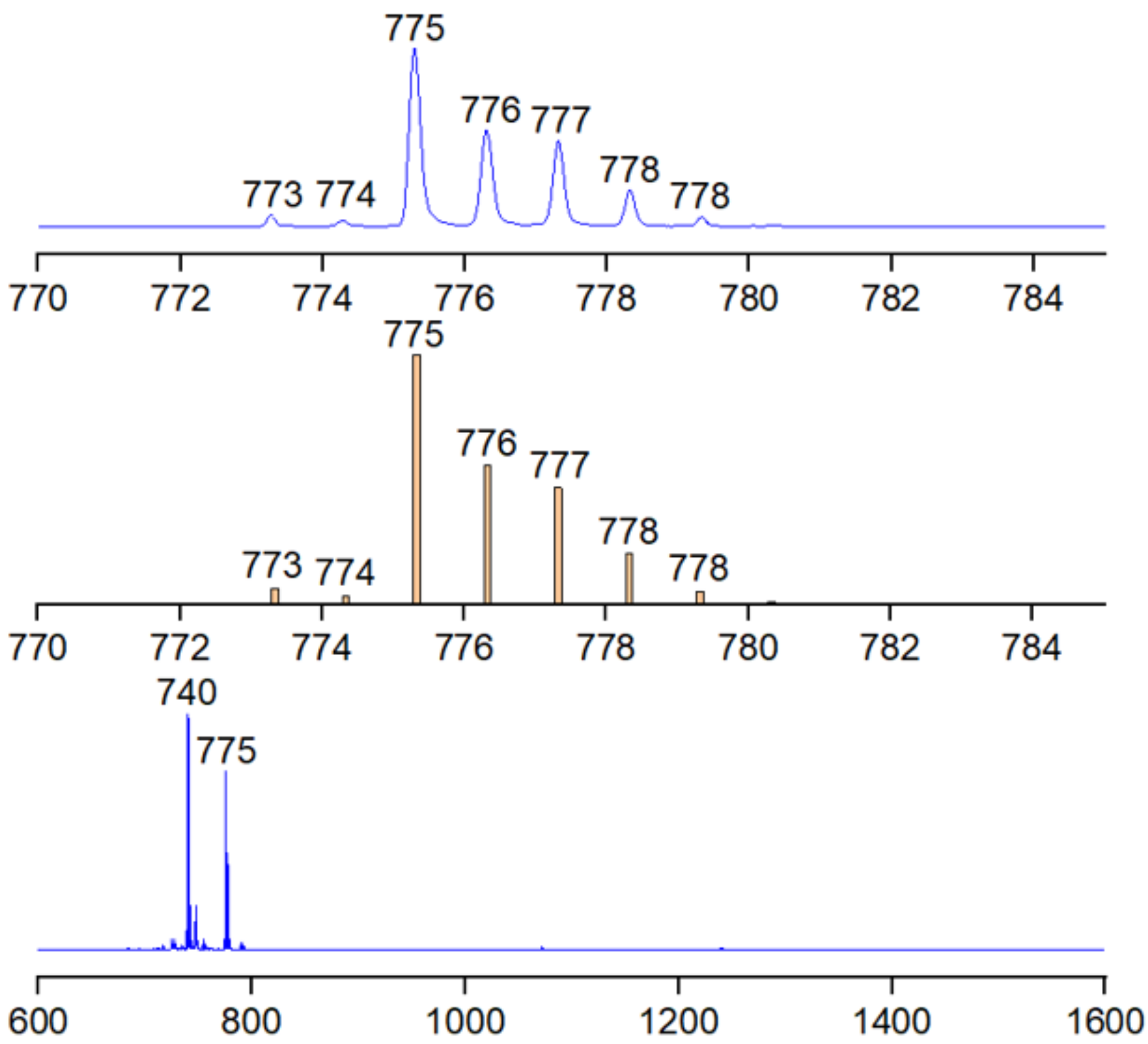

Figure S25. MALDI spectrum of **P1·FeCl** (reflection and positive mode, DCTB matrix).

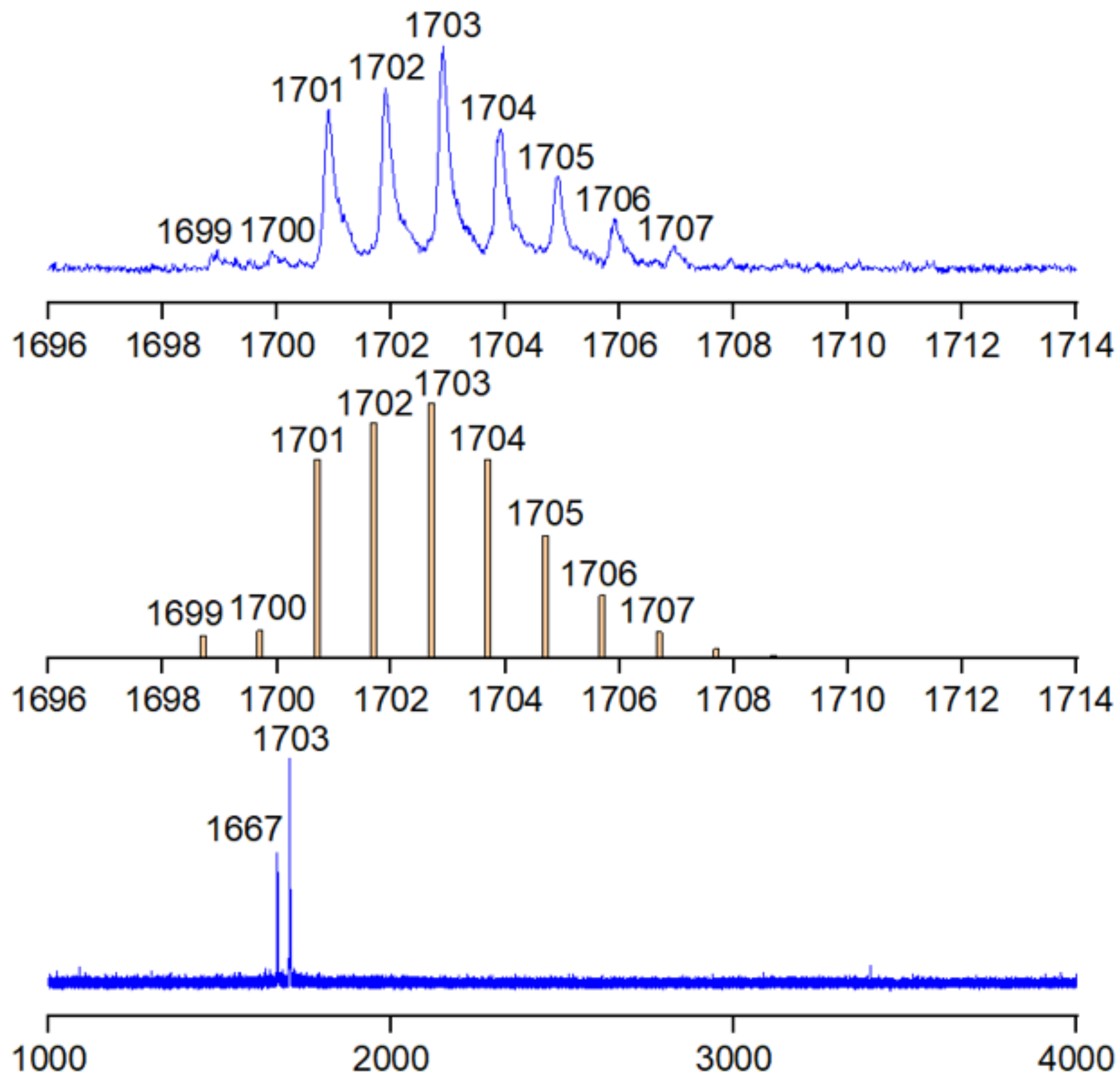

Figure S26. MALDI spectrum of ***sl*-P2·FeCl** (reflection and positive mode, DCTB matrix).

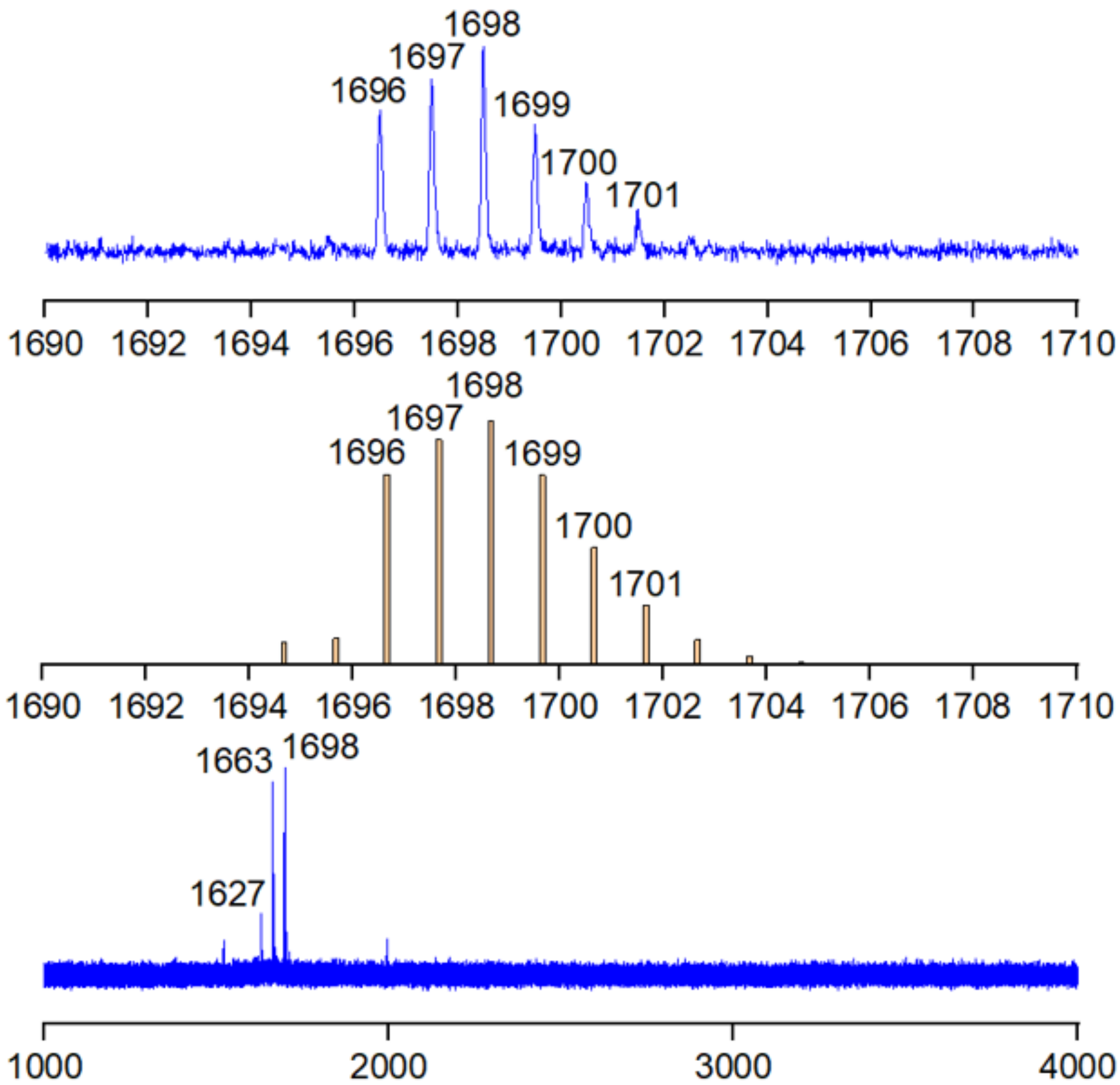

Figure S27. MALDI spectrum of ***f*-P2·FeCl** (reflection and positive mode, DCTB matrix).

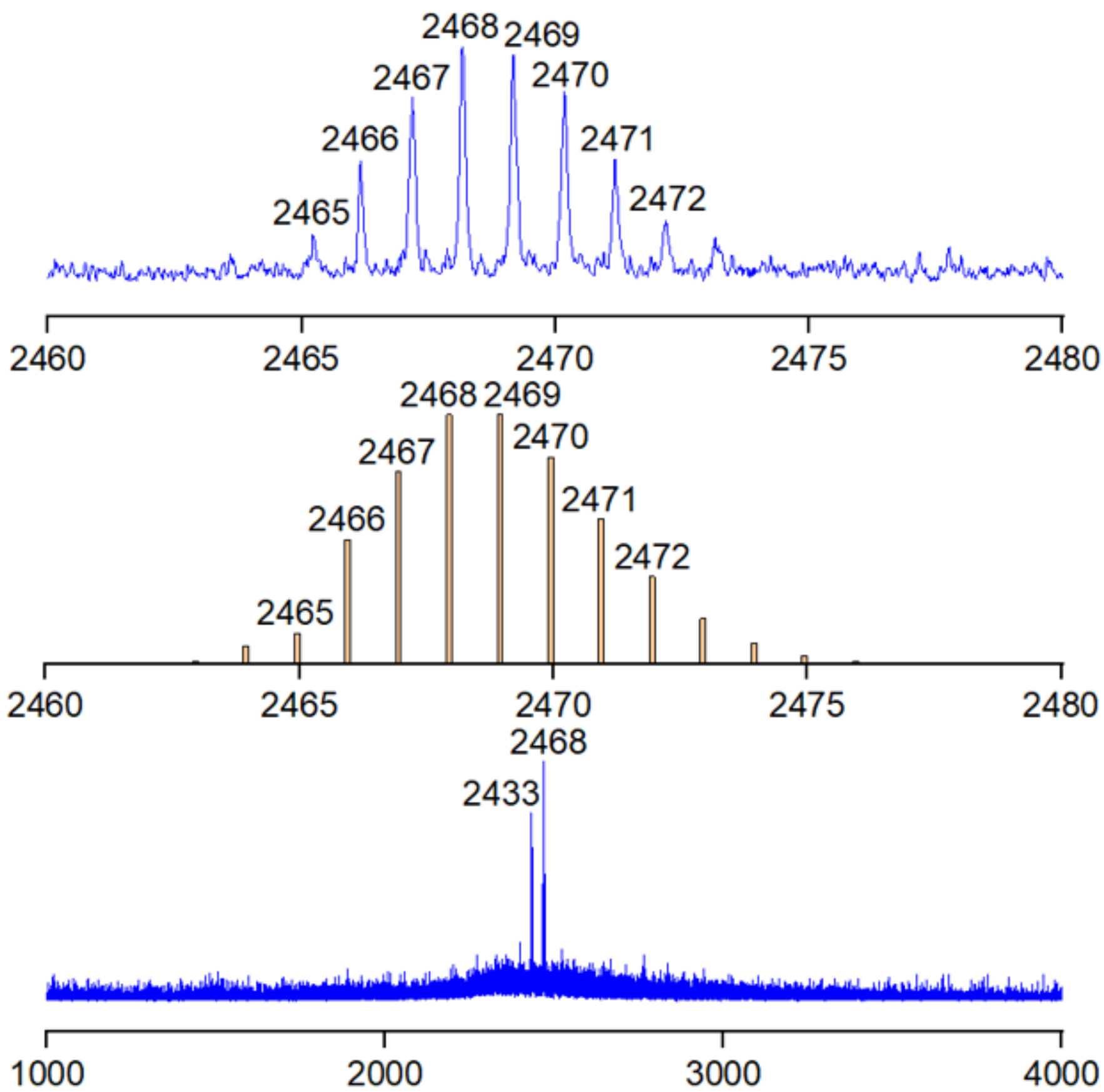

Figure S28. MALDI spectrum of ***f*-P3·FeCl** (reflection and positive mode, DCTB matrix).

6. X-Ray Crystallography

Identification code	P1·FeCl	*sl*-P2·FeCl	*f*-P2·FeCl *o*-DCB	*f*-P2·FeCl $CHCl_3$
CCDC number	**2493444**	**2493446**	**2493447**	**2493445**
Empirical formula	$C_{48}H_{52}N_4ClFe$	$C_{115}H_{118}Cl_2Fe_2N_8$	$C_{156}H_{138}N_8Cl_{18}Fe_2$	$C_{108}H_{106}Cl_2Fe_2N_8$
Formula weight	776.23	1794.77	2874.54	1698.60
Temperature/K	100.00(10)	100.00(10)	100.00(10)	100.00(10)
Crystal system	orthorhombic	monoclinic	triclinic	triclinic
Space group	$P2_12_12_1$	$P2_1/c$	P-1	P-1
a/Å	12.28790(10)	13.07880(10)	10.20809(4)	12.5285(2)
b/Å	15.70090(10)	23.24740(10)	18.30651(8)	14.8731(2)
c/Å	21.29770(10)	34.4190(2)	19.71719(7)	16.8459(2)
α/°	90	90	103.6902(3)	111.9460(10)
β/°	90	94.0060(10)	98.8717(3)	103.2570(10)
γ/°	90	90	101.8902(4)	100.9040(10)
Volume/Å^3	4108.99(5)	10439.46(11)	3422.37(3)	2699.43(7)
Z	4	4	1	1
ρ_{calc} g/cm^3	1.255	1.142	1.395	1.045
μ/mm^{-1}	3.824	3.076	5.371	2.950
F(000)	1644	3800	1488	896
Crystal size/mm^3	0.267 × 0.099 × 0.055	0.3 × 0.12 × 0.006	0.148 × 0.068 × 0.043	0.195 × 0.058 × 0.017
Radiation	Cu Kα (λ = 1.54184)	Cu Kα (λ = 1.54184)	Cu Kα (λ = 1.54184)	Cu Kα (λ = 1.54184)
Collection 2Θ range /°	6.994 to 151.954	4.59 to 117.868	4.722 to 152.004	5.99 to 152.212
Reflections collected	33542	148569	157367	54019
Independent reflections	8185	14995	14071	11027
R_{int}	0.0233	0.0248	0.0251	0.0442
R_{sigma}	0.021	0.0142	0.0108	0.0282
Data/restraints/parameters	8185/203/533	14995/2734/1481	14071/0/841	11027/0/553
Goodness-of-fit on F^2	1.031	1.039	1.104	1.050
Final R [I>=2σ (I)]	R_1 = 0.0362, wR_2 = 0.0986	R_1 = 0.0641, wR_2 = 0.1973	R_1 = 0.0355, wR_2 = 0.1025	R_1 = 0.0564, wR_2 = 0.1637
Final R [all data]	R_1 = 0.0377, wR_2 = 0.0996	R_1 = 0.0736, wR_2 = 0.2085	R_1 = 0.0365, wR_2 = 0.1033	R_1 = 0.0629, wR_2 = 0.1690
Largest diff. peak / e Å^{-3}	1.47	0.83	1.43	0.61
Largest hole / e Å^{-3}	-0.52	-0.37	-0.49	-0.69
Flack parameter	-0.0060(18)	n/a	n/a	n/a

Table S2. Crystallographic data for compounds **P1·FeCl**, ***sl*-P2·FeCl** and ***f*-P2·FeCl**.

Suitable crystals of **P1·FeCl**, ***sl*-P2·FeCl** and ***f*-P2·FeCl** were selected upon placing crystals in oil. In each case, the selected crystal was mounted on a MiTeGen 50 µm loop onto a Rigaku DW Synergy diffractometer equipped with a high-flux rotating anode source and HyPix Arc 150° detector. The crystals were kept at 100 K during data collection using an Oxford Cryosystems low-temperature device. Data were processed using CrysAlisPro software and the structures were solved with the ShelXT[S3] structure solution program using the Intrinsic Phasing solution method and by using Olex2[S4] as the graphical interface, following by refinement using least squares with ShelXL.[S5]

Crystals of **P1·FeCl** were grown by slow evaporation of a solution in a mixture of dichloromethane and methanol and then quickly manipulated in NVH immersion oil. One of the *tert*-butyl group was modelled as disordered over two positions using geometric restraints on appropriate 1,2- and 1,3-distances within split parts, either SADI or DFIX/DANG, with support of the restraints on the displacement parameters (SIMU, RIGU). There is a maximum of electron density close to iron (heavy atom) on the opposite side of the porphyrin ring, which in general is not unexpected when Cu radiation is used. Attempts to model it as a second orientation (minor disorder component) of iron using a split layer model did not provide satisfactory results.

***sl*-P2·FeCl** has been a subject of more than 15 data collections and even more crystallization attempts, screening crystals obtained from different solvents. The crystal that led to this structure was grown by vapor diffusion of methanol into a concentrated solution in toluene. The crystal was selected immediately after transferring suspension of crystals in mother liquor into Paratone oil. It was a very thin plate that diffracted poorly; hence the data collection was trimmed to 0.9 A resolution. Most of the aryl substituents and one Fe-Cl center are disordered and appropriate 1,2- and 1,3- distances within split parts were modelled using restraints, either SADI or DFIX/DANG, with support of the restraints on the displacement parameters (SIMU, RIGU). The toluene molecule is disordered and was modelled using DFIX restrains, based on the optimized distances available online at www.xray.chem.wisc.edu. Solvent masking (Olex2 implementation of BYPASS)[S6] was used to account for the electron density in voids, which indicated presence of 24 electrons per asymmetric unit (possibly methanol - 18 electrons).

***f*-P2·FeCl** was crystallized as two different solvent adducts. In both cases, only crystals of the *anti*- isomer were obtained despite multiple crystals being checked for the presence of the *syn*- isomer.

***Ortho*-dichlorobenzene solvate**
The compound was crystallized by vapor diffusion of methanol into a concentrated solution in *o*-dichlorobenzene. Suspension of crystals in mother liquor was transferred to NVH immersion oil and a suitable crystal was selected. There is an electron density peak with a weight of 1.43 next to Fe ion. Attempts to model it as another orientation of the Fe-Cl motif indicated occupancy of ca. 2% and therefore it was not included in the model as it also provided unreasonable proximity to the neighboring *o*-dichlorobenzene molecule.

Chloroform solvate
The compound was crystallized by slow evaporation of a solution in chloroform containing a few drops of methanol. Suspension of crystals was transferred to Paratone oil and a suitable crystal was selected. Initial solution indicated presence of two chloroform molecules per asymmetric unit. Unfortunately, despite many attempts to model them (splitting and modelling as disordered over two or three positions) did not provide satisfactory results. As such, solvent masking (SQUEEZE, Platon)[S7] was employed which indicated presence of 233 electrons per unit cell, which corresponds to two chloroform molecules per asymmetric unit (232 electrons per unit cell). As the presence of chloroform was clear from the initial solution, it was included in the formula which results in a discrepancy seen in the CheckCIF because the reported (with chloroform) parameters such as density, atom numbers and absorption coefficient are different than the calculated ones, based on the model which does not include the masked solvent.

Cambridge Crystallographic Data Centre (CCDC) **2493444**, **2493446, 2493445** and **2493447** records contain the supplementary crystallographic data for compounds **P1·FeCl**, ***sl*-P2·FeCl** and ***f*-P2·FeCl** (two solvatomorphs), respectively. These data can be obtained free of charge from http://www.ccdc.cam.ac.uk.

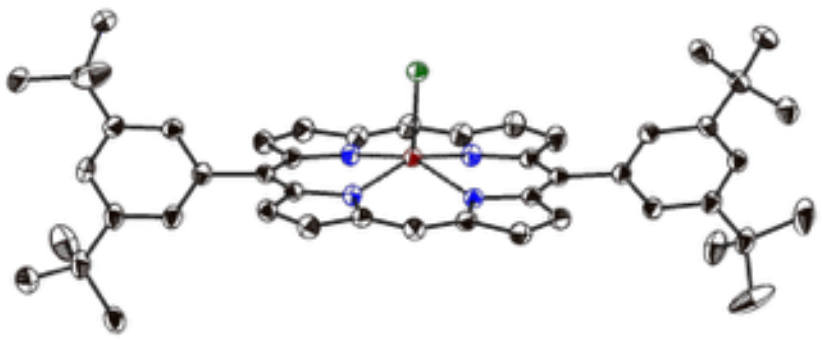

P1·FeCl
CCDC: **2493444**
Crystallized from dichloromethane
by slow evaporation at 20 °C

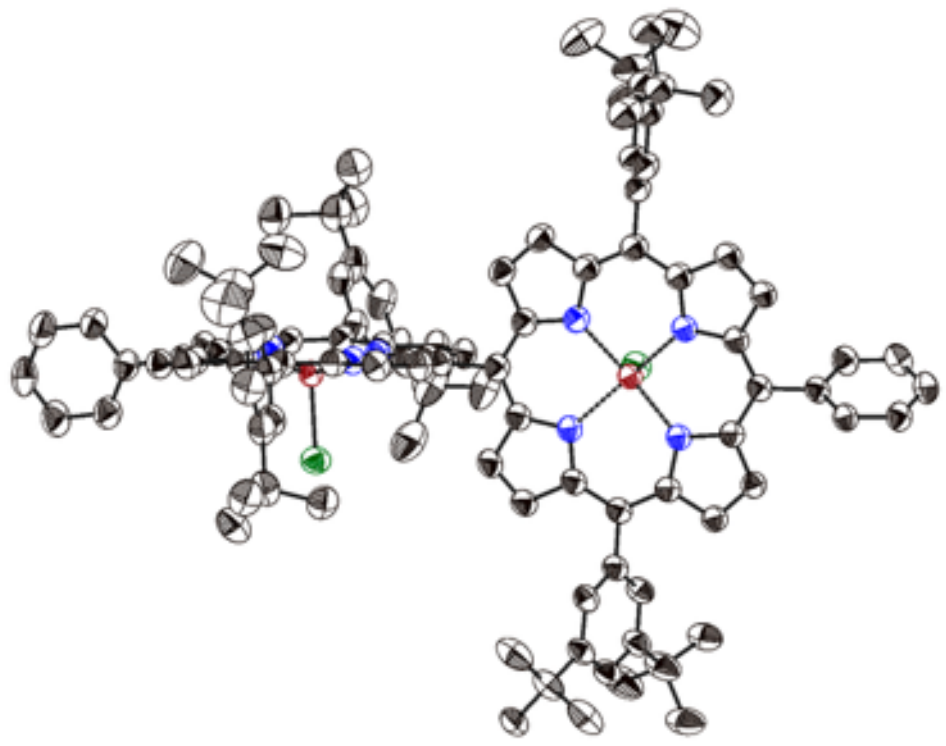

***sl*-P2·FeCl**
CCDC: **2493446**
Crystallized from toluene/MeOH
by vapor diffusion at 20 °C

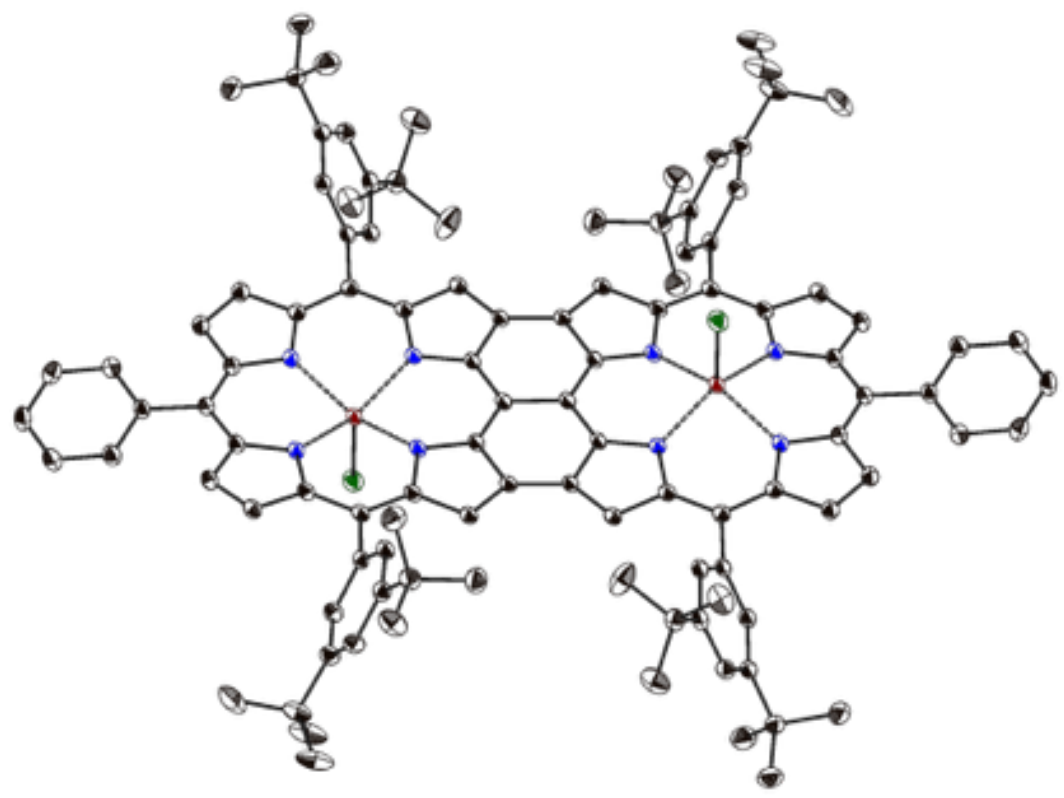

***f*-P2·FeCl**
CCDC: **2493445** and **2493447**
Crystallized from o-dichlorobenzene/MeOH of chloroform/MeOH
by vapor diffusion or slow evaporation at 20°C

Additional information of the crystals

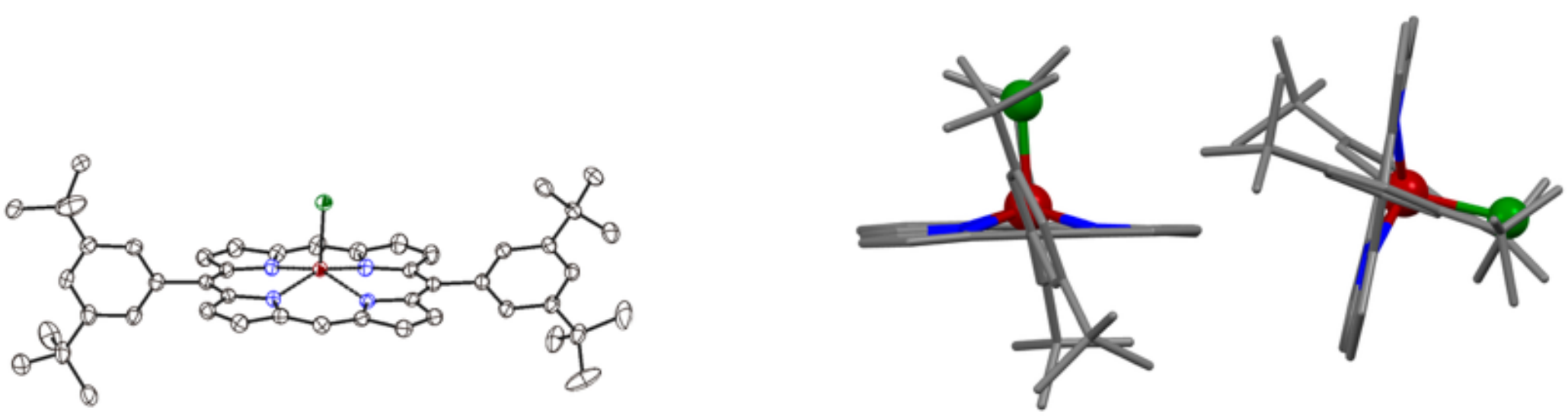

Figure S29. Asymmetric unit and relative orientation of neighboring porphyrins in **P1·FeCl** crystals. The displacement ellipsoids were set at 50% probability level. Hydrogen atoms and minor disorder components were removed for clarity.

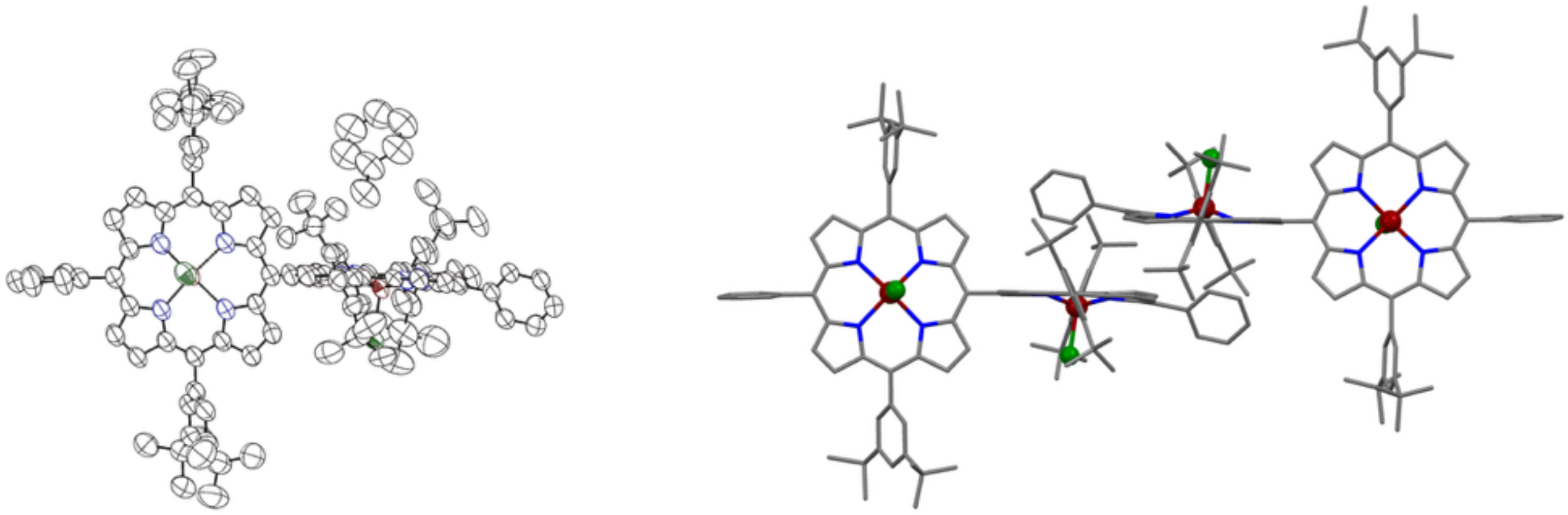

Figure S30. Asymmetric unit and relative orientation of neighboring porphyrins in ***sl*-P2·FeCl** crystals. The displacement ellipsoids were set at 50% probability level. Hydrogen atoms were removed for clarity.

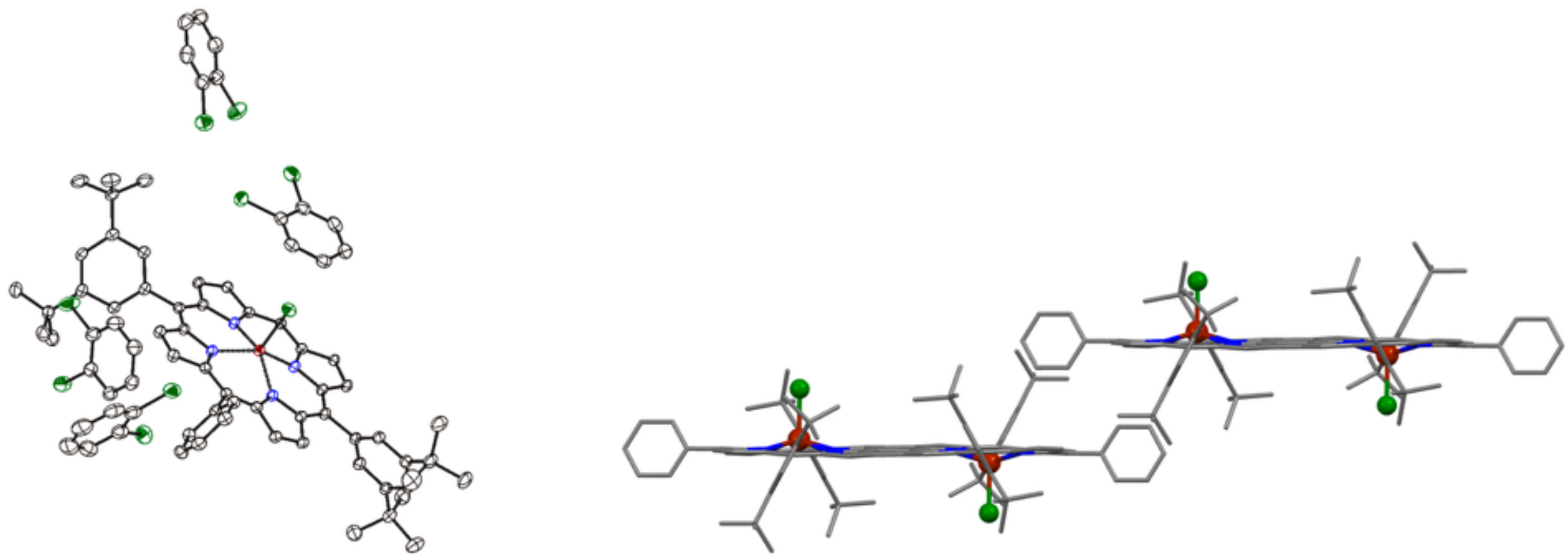

Figure S31 Asymmetric unit and relative orientation of neighboring porphyrins in ***f*-P2·FeCl *o*-dichlorobenzene solvate** crystals. The displacement ellipsoids were set at 50% probability level. Hydrogen atoms and minor disorder components were removed for clarity.

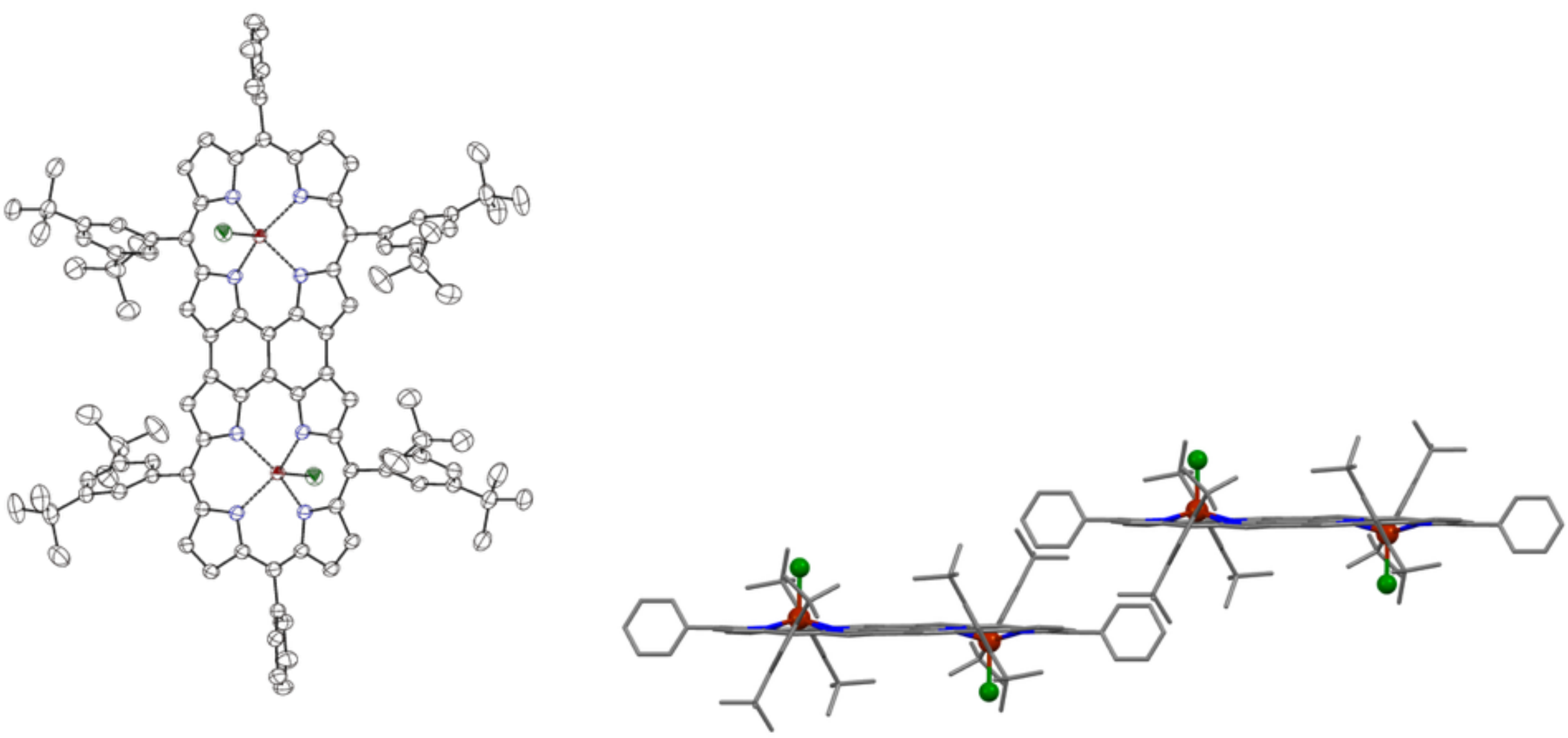

Figure S32. Asymmetric unit and relative orientation of neighboring porphyrins in ***f*-P2·FeCl $CHCl_3$ solvate** crystals. The displacement ellipsoids were set at 50% probability level. Hydrogen atoms and minor disorder components were removed for clarity.

7. EPR Spectroscopy

We recorded CW EPR at 100 K of **P1·FeCl**, ***sl*-P2·FeCl**, ***f*-P2·FeCl** and ***f*-P3·FeCl** using solid-state powder samples. In general Fe(III) porphyrins give broad EPR spectra compared with other *d* metal ions due to the fast relaxation time. A spectrum of ***f*-P3·FeCl** could not be recorded due to the limited quantity of material. The EPR spectra of **P1·FeCl**, ***sl*-P2·FeCl**, and ***f*-P2·FeCl** can be satisfactory simulated using similar parameters to those obtained from SQUID magnetometry (Figs. S33, S34 and S35 respectively) despite the use of an oversimplified model.

CW-EPR spectra were simulated using EasySpin software.[S8]

7.1 Experimental Methods

All EPR measurements were recorded on a Bruker Elexsys E580 spectrometer X-band (9.5 GHz). Low temperature measurements were obtained using an Oxford Instruments ESR900 continuous flow nitrogen cryostat. Temperatures were controlled with an Oxford Instruments ITC503. The samples **P1·FeCl**, ***sl*-P2·FeCl**, ***f*-P2·FeCl** and ***f*-P3·FeCl** were prepared as pure solids under argon atmosphere.

Optimization of the systems for EPR

All CW spectra were recorded at 100 K. For the compounds **P1·FeCl**, ***sl*-P2·FeCl**, ***f*-P2·FeCl** and ***f*-P3·FeCl** we recorded spectra for the pure solids under argon atmosphere due to the low solubility. These iron compounds give broad spectra and poor signal compared with other *d* metal ions, presumably due to the fast relaxation time. The amount available of ***f*-P3·FeCl** was not enough to obtain a suitable spectrum.

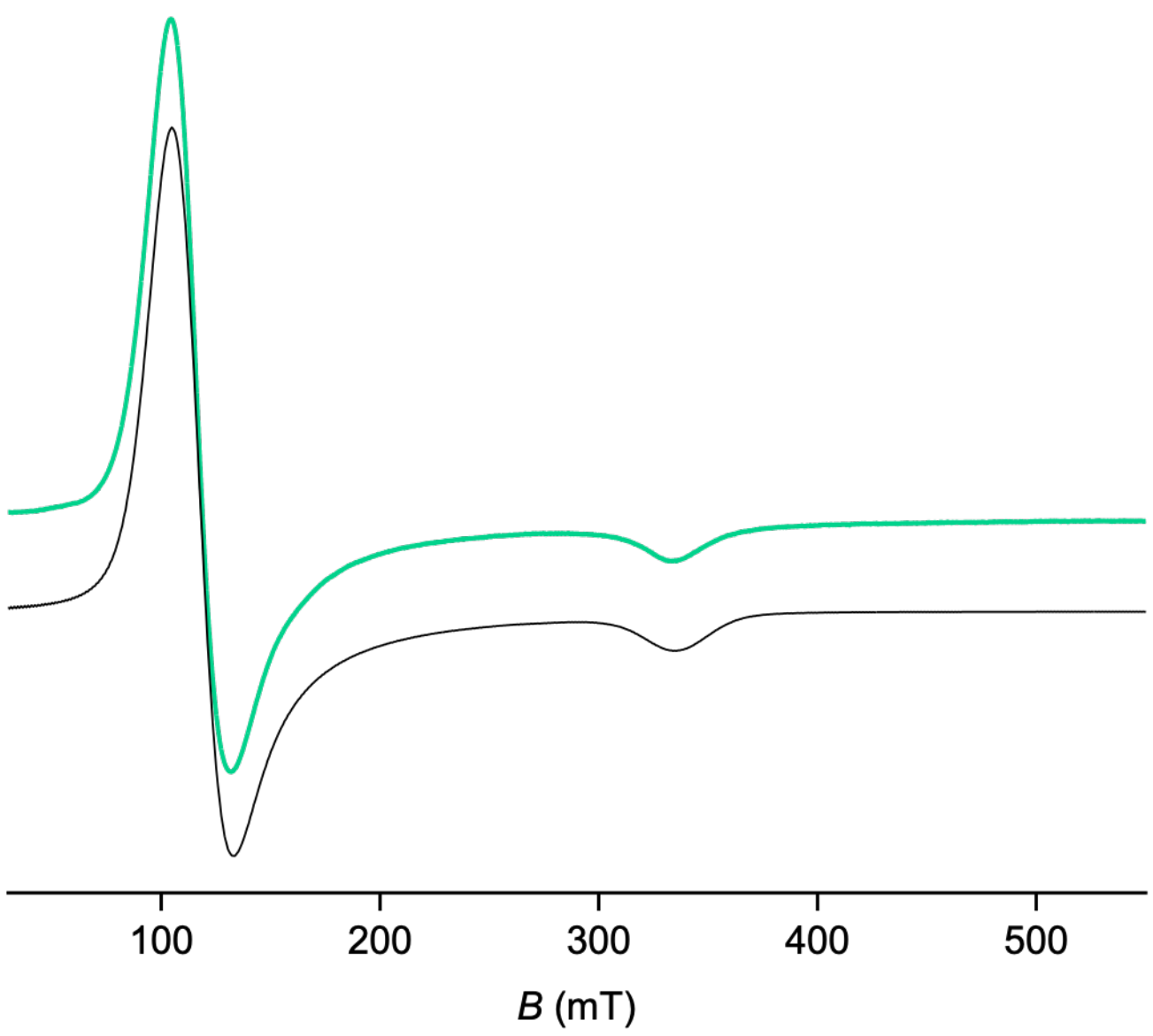

Figure S33. CW spectra at X-Band of **P1·FeCl** in solid state at 100 K. Simulation parameters: $g_{xy} = g_z = 2.00$ and $D \geq 50$ GHz.

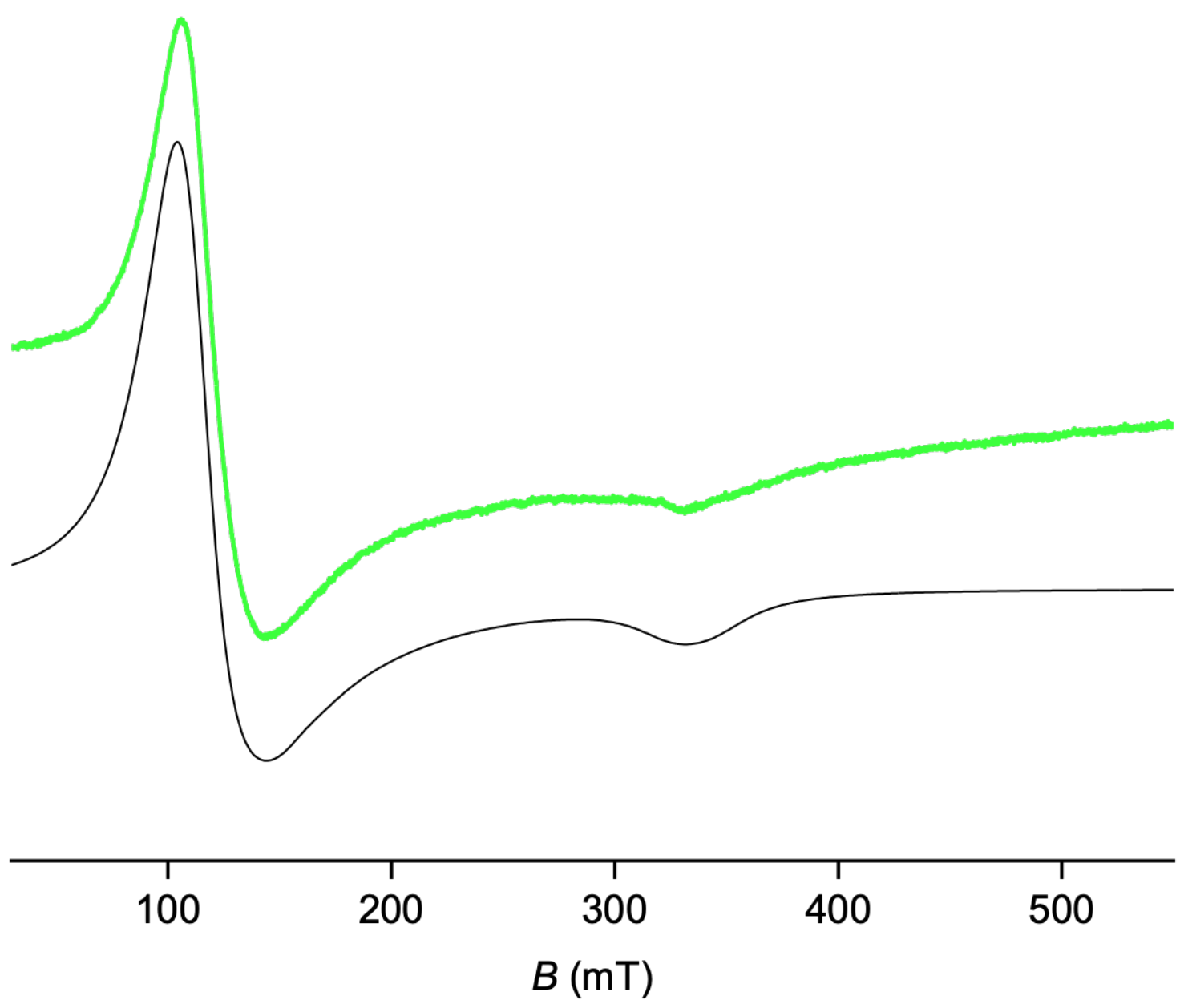

Figure S34. CW spectra at X-Band of ***sl*-P2·FeCl** in solid state at 100 K. Simulation parameters: g(Fe$_1$) = g(Fe$_2$): $g_{xy} = g_z = 2.00$, D(Fe$_1$) = D(Fe$_2$) ≥ 73 GHz, –130 ≤ J ≥ 130 MHz (-2JS_1S_2 antiferromagnetic, positive values are ferromagnetic).

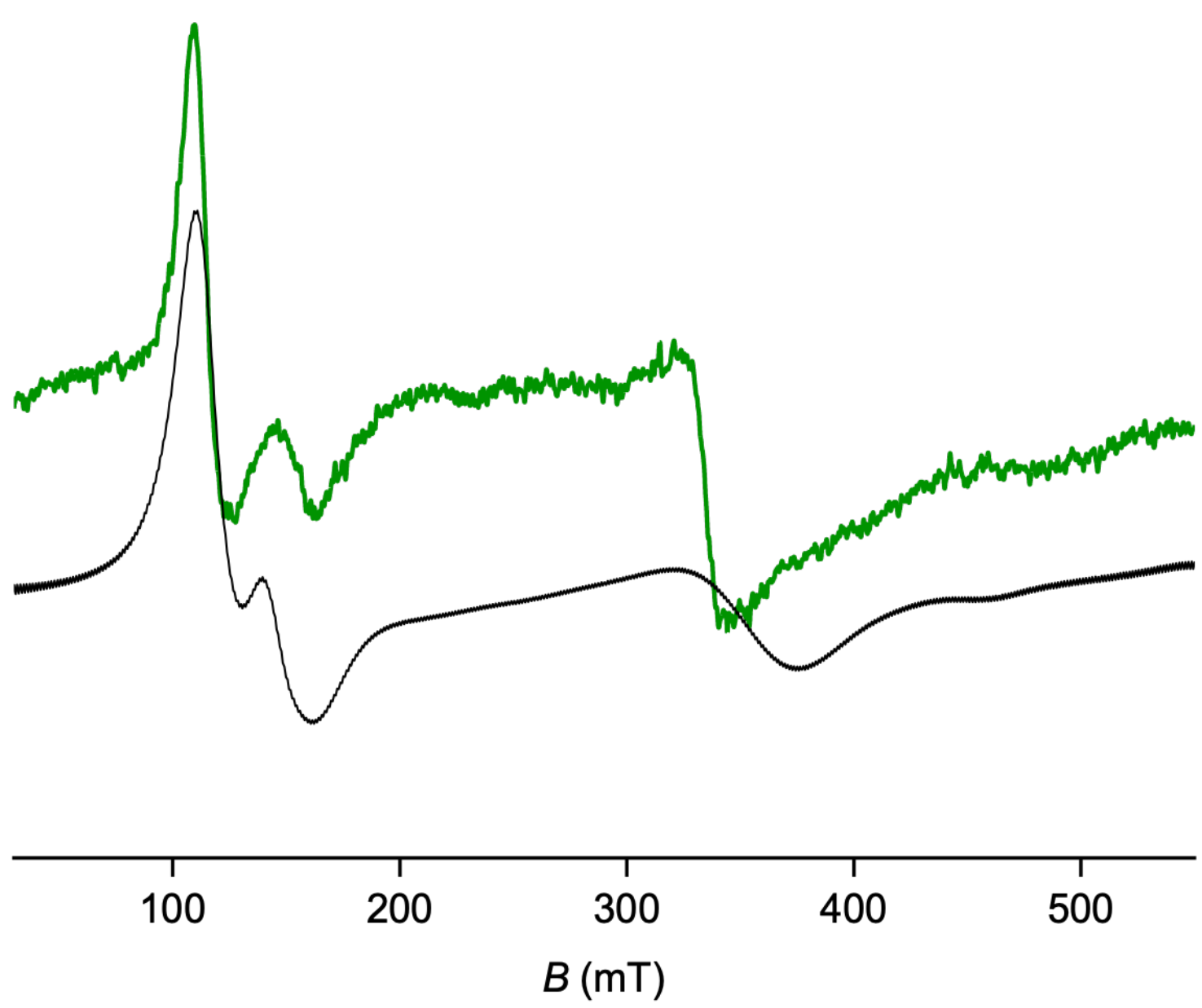

Figure S35. CW spectra at X-Band of ***f*-P2·FeCl** in solid state at 100 K. Simulation parameters: g(Fe$_1$) = g(Fe$_2$): $g_{xy} = g_z = 2.15$, D(Fe$_1$) = D(Fe$_2$) = 73.749 GHz, *DStrain*(Fe$_1$) = *DStrain*(Fe$_2$) = 5800 1450, J = –46.5 GHz (–2JS_1S_2 antiferromagnetic).

8. SQUID Magnetometry

8.1 Direct current SQUID measurements

Variable-temperature direct current (dc) magnetic susceptibility data were collected on a Quantum Design MPMS-XL SQUID magnetometer equipped with a 7 T magnet and operating in the 2–300 K range, under a magnetic field of 1000 Oe (0.1 T). The diamagnetic contribution of the sample holder was subtracted from the raw data. Pascal's constants were used to estimate the diamagnetic contribution of the different complexes, which were subtracted from the experimental susceptibilities to give the molar paramagnetic susceptibilities (χ_M).

Simultaneous fitting of $\chi_M T$ and M of the data was performed using PHI program.[S9] Two different models called 'Fitting 1' and 'Fitting 2' were used for data set, so as to provide insight into the reliability of the resulting data. The difference between the models relates to which parameters are fixed, as indicated below. The black lines in Figures S37–S40 are calculated curves from fitting model 1; calculated curves for fitting model 2 are not shown because they look identical.

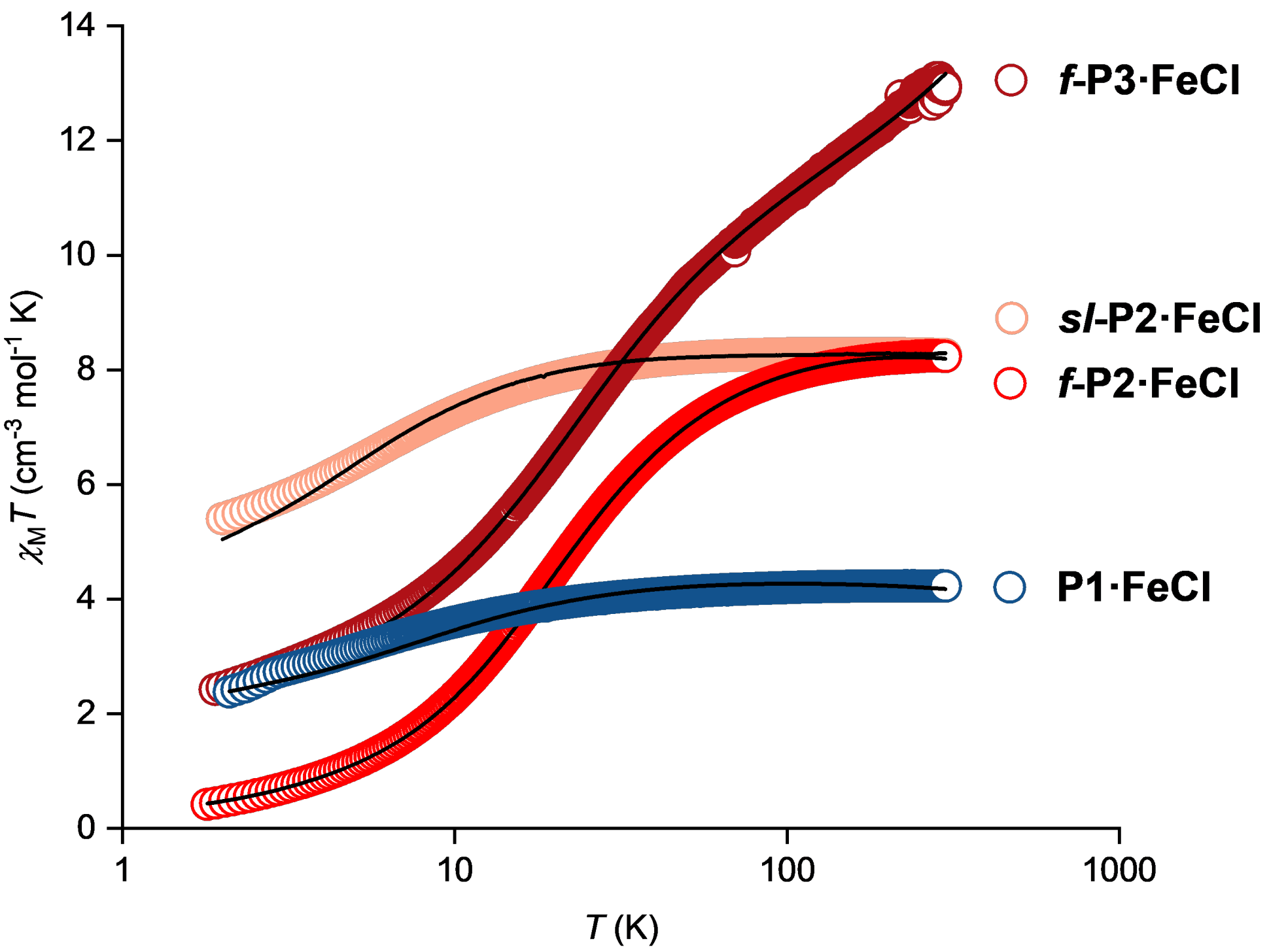

Figure S36. Magnetic susceptibility data for **P1·FeCl**, ***sl*-P2·FeCl**, ***f*-P2·FeCl** and ***f*-P3·FeCl**, in solid state, as a function of temperature, plotted on a logarithmic temperature scale. (Fitting lines in black.) **P1·FeCl** exhibits a clear decrease of $\chi_M T$ below ca. 10 K, due to the zero-field splitting of the Fe ion. This behavior is consistent with the fitted axial anisotropy parameter, for which (D/k_B) is of the order of a few kelvin. A similar low-temperature decrease is observed for ***sl*-P2·FeCl**, indicating that in this case the magnetic response is still largely dominated by the single-ion anisotropy contribution, with only weak exchange effects. In contrast, the ***f*-P2·FeCl** and ***f*-P3·FeCl** display a more pronounced decrease of $\chi_M T$, starting at higher temperatures. This indicates that, in these fused systems, the effect of zero-field splitting is partly masked by the stronger antiferromagnetic exchange interactions between the Fe centers.

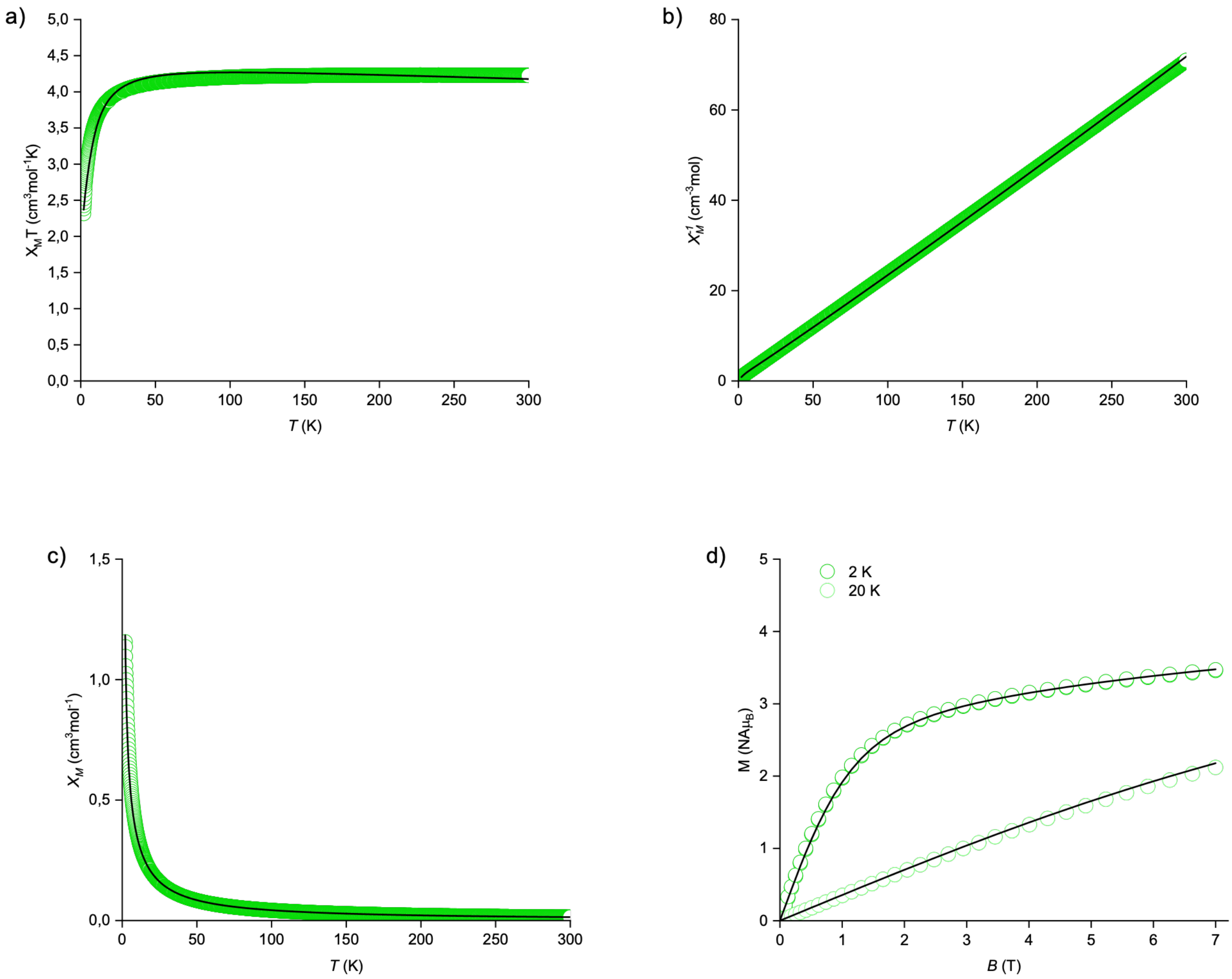

Figure S37. a) Temperature-dependent $\chi_M T$, b) χ_M^{-1}, c) χ_M, and d) magnetization vs magnetic field for complex **P1·FeCl**. The black solid line represents the simultaneous fit of all the plots using PHI program.[S9] The static magnetization was measured at an applied magnetic field H_{dc} of 1000 Oe.

Fitting 1: *g* was fixed to 2.00 (based on fitting the EPR data, Figure S33), *D* = 99.23±0.09 GHz, *zj* = –0.60± 0.01 GHz (where *zj* is a measure of the intermolecular interactions).

Fitting 2: *g* = 1.966±0.001, *D* = 85.5±0.1 GHz; *zj* fixed to 0.00 GHz.

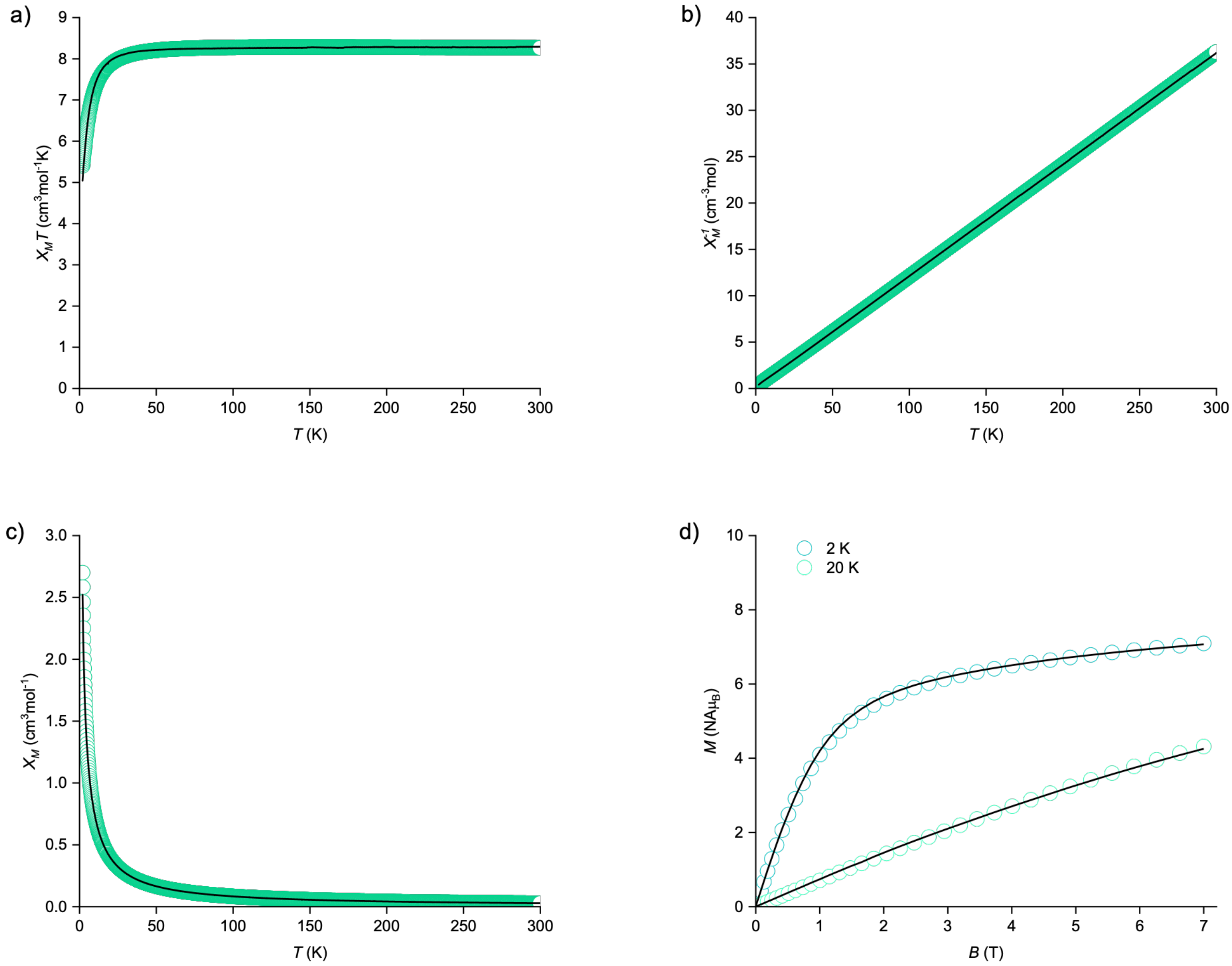

Figure S38. a) Temperature-dependent $\chi_M T$, b) χ_M^{-1}, c) χ_M and d) magnetization vs magnetic field for complex ***sl*-P2·FeCl**. The black solid line represents the simultaneous fit of all the plots using PHI program.[S9] The static magnetization was measured at an applied magnetic field H_{dc} of 1000 Oe.

Fitting 1: g was fixed to 2.00, $D_1 = D_2$ = 73.75±0.15 GHz, J = 0.60±0.01 GHz (ferromagnetic), zj = –0.256± 0.004 GHz, 17.8% of monomeric impurity was considered.

Fitting 2: g was fixed to 2.00, $D_1 = D_2$ = 72.28±0.08 GHz, J = 0.91±0.01 GHz (ferromagnetic), 10.8% of monomeric impurity was considered; zj fixed to 0.00 GHz.

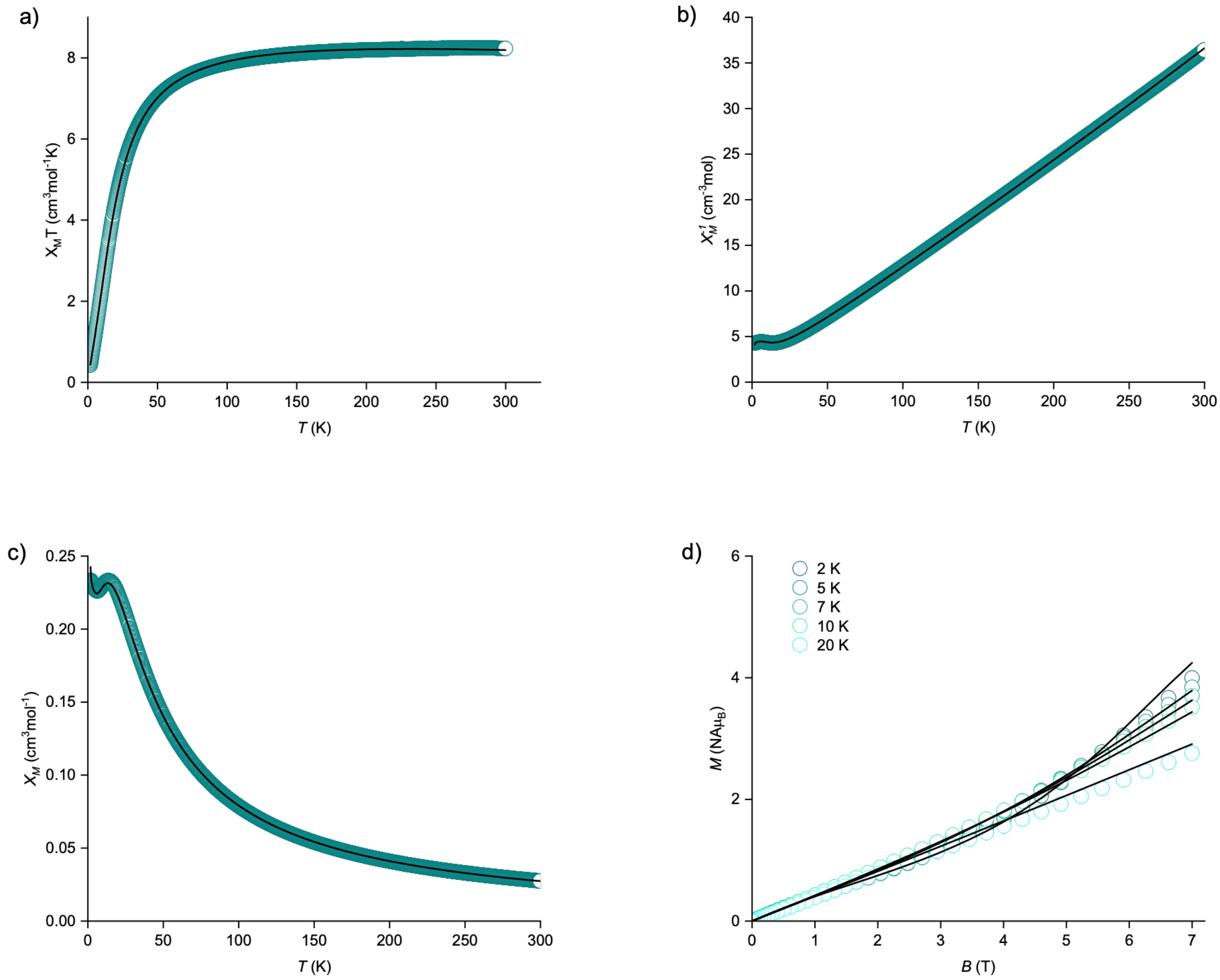

Figure S39. a) Temperature-dependent $\chi_M T$, b) χ_M^{-1}, c) χ_M and d) magnetization vs magnetic field (d) for complex ***f*-P2·FeCl**. The black solid line represents the simultaneous fit of all the plots using PHI program.[S9]. The static magnetization was measured at an applied magnetic field H_{dc} of 1000 Oe.

Fitting 1: g was fixed to 2.00, zero-field splitting was fixed to $D_1 = D_2 = 73.75\pm0.15$ GHz, $J = -32.98\pm0.02$ GHz (antiferromagnetic), $zj = -0.75\pm0.01$ GHz, 8.0% of monomeric impurity was considered.

Fitting 2: g was fixed to 2.00, $D_1 = D_2 = 62.39\pm0.07$ GHz, $J = -32.02\pm0.04$ GHz (antiferromagnetic), 3.5% of monomeric impurity was considered; zj fixed to 0.00 GHz.

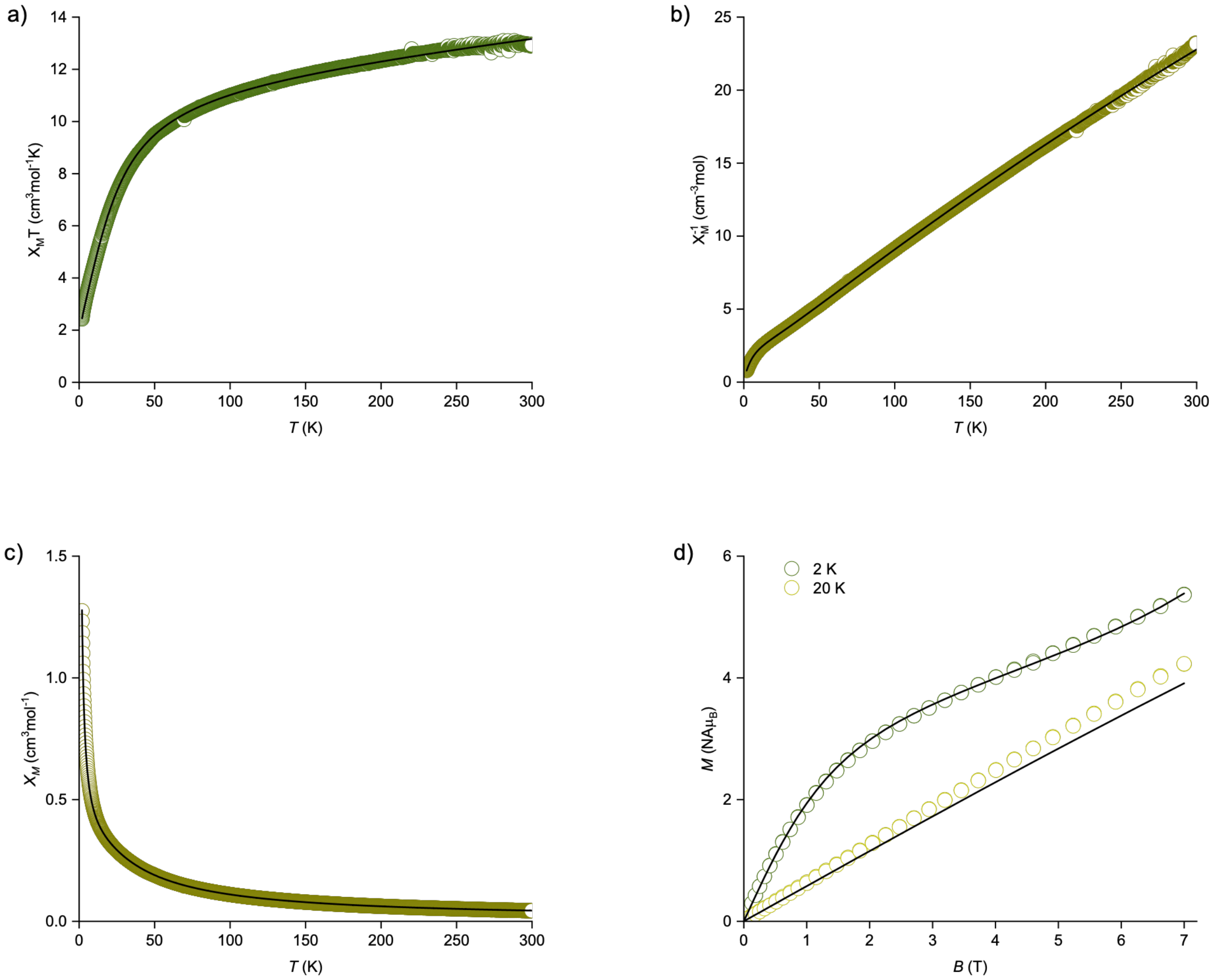

Figure S40. a) Temperature-dependent $\chi_M T$, b) χ_M^{-1}, c) χ_M and d) magnetization vs magnetic field (d) for complex ***f*-P3·FeCl**. The black solid line represents the simultaneous fit of all the plots using PHI program.[S9] The static magnetization was measured at an applied magnetic field H_{dc} of 1000 Oe.

Fitting 1: g was fixed to 2.00, zero-field splitting was fixed to $D_1 = D_2 = D_3$ =73.75±0.15 GHz, $J_{12} = J_{23}$ = –24.28±0.06 GHz (antiferromagnetic), J_{13} = –10.79±0.10 GHz (antiferromagnetic), zj = –0.21±0.02 GHz, 17.0% of monomeric impurity was considered. See residuals analysis in Figure S41.

Fitting 2: g was fixed to 2.00, $D_1 = D_2 = D_3$ =81.08±1.06 GHz, $J_{12} = J_{23}$ = –24.20±0.06 GHz (antiferromagnetic), J_{13} = –3.89±0.24 GHz (antiferromagnetic), zj = –2.88±0.02 GHz, 7.6% of monomeric impurity was considered.

8.2 Summary of SQUID results

Table S3. Summary of SQUID parameters **Fitting 1**.

**Fitting 1**	*g*	*D /GHz*	*zj /GHz*	*Monomer Impurity/ %*	J_{12} /GHz	J_{13} /GHz	R^2
**P1·FeCl**	2.00 (fixed)	99.23±0.09	−0.60± 0.01	-	-	-	0.9897
***sl*-P2·FeCl**	2.00 (fixed)	$D_1 = D_2 =$ 73.75±0.15	−0.256± 0.004	17.8	0.60±0.01	-	0.9983
***f*-P2·FeCl**	2.00 (fixed)	$D_1 = D_2 =$ 73.75±0.15 (fixed)	−0.75±0.01	8.0	−32.98±0.02	-	0.9991
***f*-P3·FeCl**	2.00 (fixed)	$D_1 = D_2 = D_3 =$ 73.75±0.15 (fixed)	−0.21±0.02	17.0	−24.28±0.06	−10.79±0.10	0.9988

Table S4. Summary of SQUID parameters **Fitting 2**.

**Fitting 2**	*g*	*D /GHz*	*zj /GHz*	*Monomer Impurity/ %*	J_{12} /GHz	J_{13} /GHz	R^2
**P1·FeCl**	1.966±0.001	85.45±0.1	-	n/a	-	-	0.9978
***sl*-P2·FeCl**	2.00 (fixed)	$D_1 = D_2 =$ 72.28±0.08	-	10.8	0.91±0.01	-	0.9973
***f*-P2·FeCl**	2.00 (fixed)	$D_1 = D_2 =$ 62.39±0.07	-	3.5	−32.02±0.04	-	0.9987
***f*-P3·FeCl**	2.00 (fixed)	$D_1 = D_2 = D_3 =$ 81.08±1.06	−2.88±0.02	7.6	−24.20±0.06	−3.89±0.24	0.9962

8.3 Analysis of the residuals

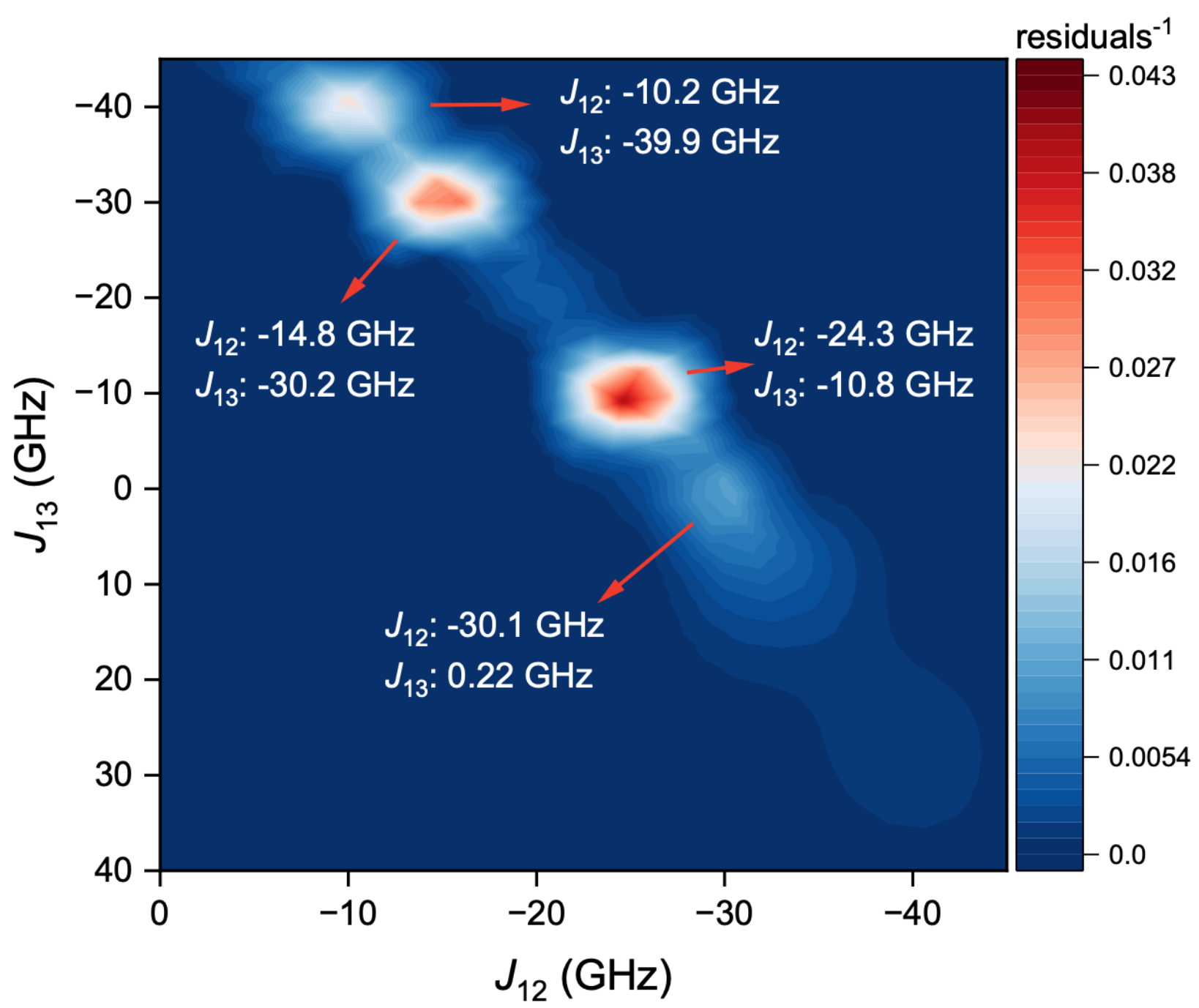

Figure S41. Analysis of the goodness of the fit for ***f*-P3·FeCl**. Reverse residuals of the fittings for different $J_{12} = J_{23}$ and J_{13} values (Fitting Method 1 with fixed values of g = 2.00 and $D_1 = D_2 = D_3$ = 73.75±0.15).

9. Computational Details

All structures were optimized at the M06-2X/def2-SVP level of theory, which was also used for computing chemical shifts. To investigate the effect of exact exchange (EE), broken symmetry calculations were done using a series of functionals: TPSSh (10% EE),[S10] B3LYP* (15%),[S11] B3LYP (20%),[S12] OX-B3LYP (19–100%),[S13] PBE0 (25%),[S14] and M06-2X (54%).[S15] PBE0, OX-B3LYP, and M06-2X (which is highly empirical) produced good agreement with experiment, suggesting that ~25% exchange is appropriate for these systems. *J* values were computed using Yamaguchi's equation.[S10,S16] All calculations were done using ORCA.[S17]

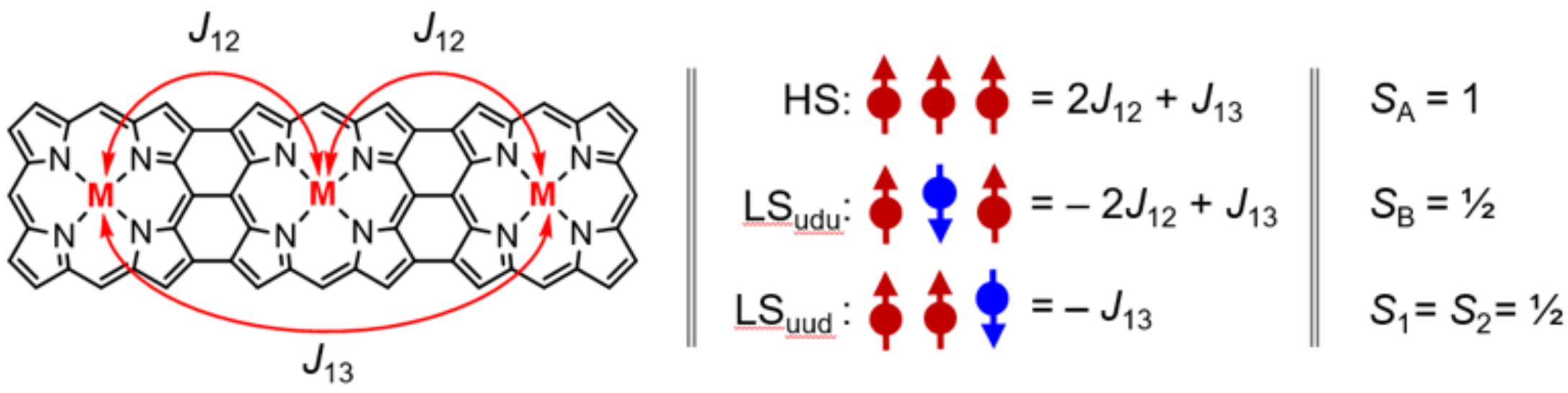

$$J_{BS} = \frac{E_{HS} - E_{LS}}{\langle S^2_{HS} \rangle - \langle S^2_{LS} \rangle}$$

$$HS - LS = 2J_{BS}S_AS_B$$

$$HS - LS_{udu} = 4J_{12}S_1S_2 = 2J_{BS}S_AS_B \,;\; J_{12} = J_{BS}$$

$$HS - LS_{uud} = 2(J_{12} + J_{13})S_1S_2 = 2J_{BS}S_AS_B$$

$$J_{12} + J_{13} = 2J_{BS}$$

Figure S42. Theoretical modelling of the trimeric systems.

The NMR shift calculations were performed considering the population of energy levels according to the Boltzmann distribution and including Zeeman effect at 298 K (M06-2X).

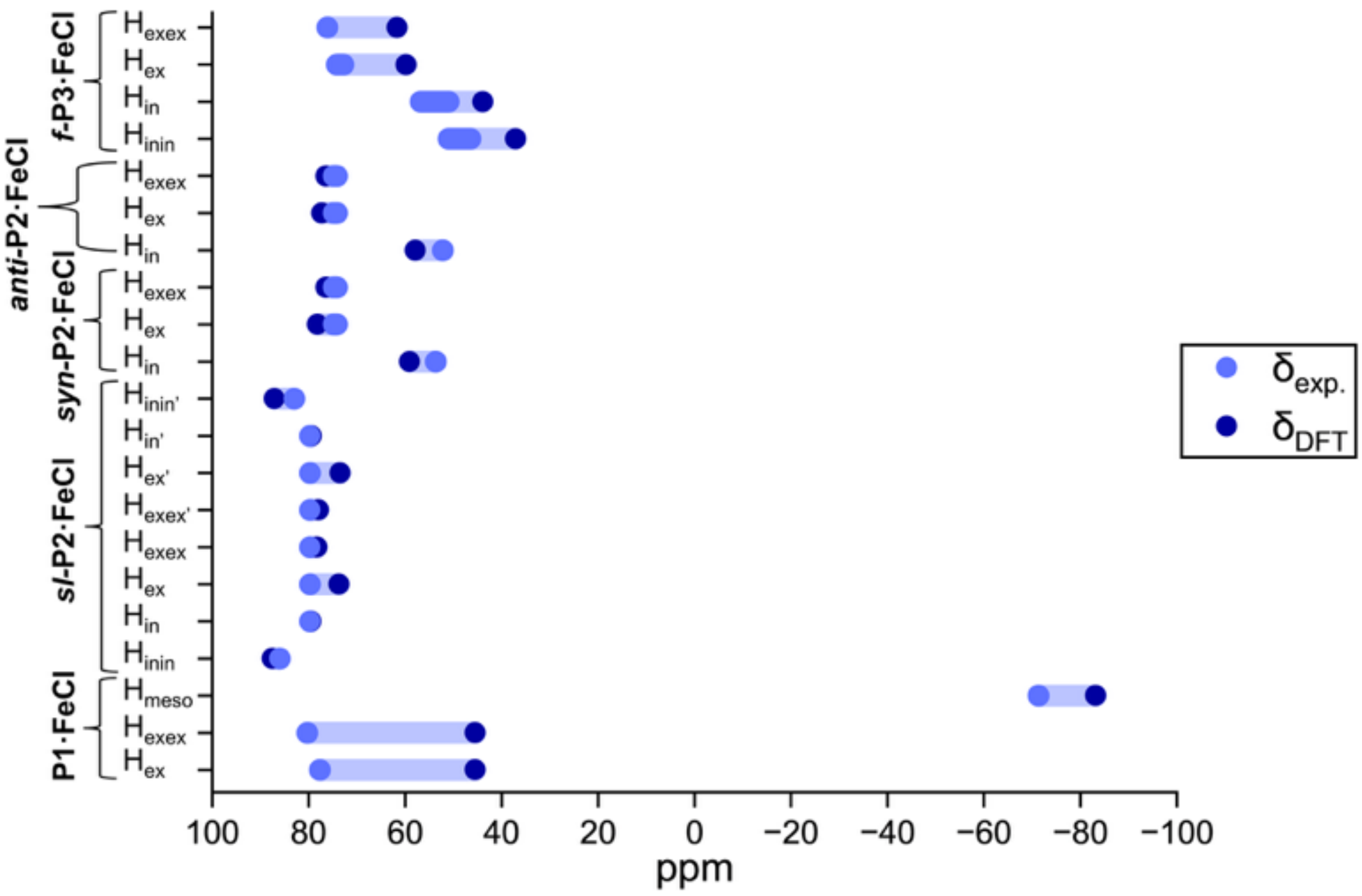

Figure S43. Calculated and experimental NMR shifts (100 to –100 ppm) of Fe(III) porphyrins studied. For ***f*-P3·FeCl**, we included values obtained from the averaging of the three stereoisomers.

Table S5. J_{12} and J_{13} values calculated using various density functional approximations (data from Figure 5a). Proportion of exact exchange is denoted in parentheses.

	SQUID[a]	OX-B3LYP	B3LYP (20%)	B3LYP* (15%)	PBE0 (25%)	M06-2X (54%)
***sl*-P2·FeCl**						
J_{12} (GHz)	0.60±0.01	0.27	0.37	0.20	0.30	0.37
***f*-P2·FeCl**						
J_{12} (GHz)	–32.98±0.02	–45.32	–43.86	–46.35	–34.66	–27.53
***f*-P3·FeCl**[b] J_{12} (GHz)	–24.28±0.06	–38.57	–38.50	–38.7	–29.04	–21.32
J_{13} (GHz)	–10.79±0.10	–10.22	–8.51	–10.30	–10.24	–17.77

a) Experimental value from Fitting 1 (Table S3). b) Values of J_{12} and J_{13} were calculated for the most stable stereoisomers (*anti-**f**-***P2·FeCl** and *anti-anti-**f**-***P3·FeCl**), but other stereoisomers give almost the same values, as demonstrated by the data in Tables S6 and S7.

Table S6. J_{12} values and relative energies for isomers of ***f*-P2·FeCl** calculated using various density functional approximations (with proportion of exact exchange in parentheses), named according to the relative position of the chlorine atoms. The main text, Figure 5a, and Table S5 only discuss *J* values for lowest-energy isomers.

***f*-P2·FeCl**	*OX-B3LYP*	*B3LYP (20%)*	*B3LYP*(15%)*	*PBE0 (25%)*	*M06-2X (54%)*
J_{12} (GHz) *anti*	–45.32	–43.86	–46.35	–34.66	–27.53
J_{12} (GHz) *syn*	–48.27	–46.68	–37.05	–37.05	–28.84
J_{12} (GHz) average	–46.79	–45.27	–41.70	–35.86	–28.19
$E_{syn} – E_{anti}$ (kcal/mol)	0.131	0.126	0.120	0.152	0.149

Table S7. J_{13} values for isomers of ***f*-P2·FeCl** calculated using M06-2X, named according to the relative position of the chlorine atoms. Relative energies of isomers (in kcal/mol) are denoted in parentheses. The main text, Figure 5a, and Table S5 only discuss *J* values for lowest-energy isomers.

***f*-P3·FeCl**	*anti-anti* (0.00)	*syn-anti* (0.19)	*syn-syn* (0.41)	*average*
J_{12} (GHz) M06-2X	–21.32	–21.88	–22.18	–21.79
J_{13} (GHz) M06-2X	–17.77	–17.09	–17.99	–17.62

10. References

S1. Jradi, F. M.; O'Neil, D.; Kang, X.; Wong, J.; Szymanski, P.; Parker, T. C.; Anderson, H. L.; El-Sayed, M. A.; Marder, S. R. A Step toward efficient panchromatic multi-chromophoric sensitizers for dye sensitized solar cells. *Chem. Mater.* **2015***, 27*, 6305–6313. DOI: 10.1021/acs.chemmater.5b02006.

S2. Pozo, I.; Huang, Z.; Lombardi, F.; Alexandropoulos, D. I.; Kong, F.; Slota, M.; Tkach, I.; Bennati, M.; Deng, J.-R.; Stawski, W.; Horton, P. N.; Coles, S. J.; Myers, W. K.; Bogani, L.; Anderson, H. L. Enhanced coherence by coupling spins through a delocalized π-system: Vanadyl porphyrin dimers. *Chem* **2024**, *10*, 299–316. DOI: 10.1016/j.chempr.2023.09.013.

S3. Sheldrick, G. M. SHELXT - Integrated space-group and crystal-structure determination. *Acta Cryst.* **2015**, *A71*, 3–8. DOI: 10.1107/S2053273314026370.

S4. Dolomanov, O. V.; Bourhis, L. J.; Gildea, R. J.; Howard, J. A. K.; Puschmann, H. OLEX2: a complete structure solution, refinement and analysis program. *J. Appl. Cryst.* **2009**, *42*, 339–341. DOI: 10.1107/S0021889808042726.

S5. Sheldrick, G. M. Crystal structure refinement with SHELXL. *Acta Cryst.* **2015**, *C71*, 3–8. DOI: 10.1107/S2053229614024218.

S6. van der Sluis, P.; Spek A. L. BYPASS: an effective method for the refinement of crystal structures containing disordered solvent regions. *Acta Cryst.* **1990**, *A46*, 194–201. DOI: 10.1107/S0108767389011189.

S7. Spek, A. L. PLATON SQUEEZE: a tool for the calculation of the disordered solvent contribution to the calculated structure factors. *Acta Cryst.* **2015**, *C71*, 9–18. DOI: 10.1107/S2053229614024929.

S8. Stoll, S.; Schweiger, A. EasySpin, a comprehensive software package for spectral simulation and analysis in EPR. *J. Magn. Reson.* **2006**, *178*, 42–55. DOI: 10.1016/j.jmr.2005.08.013.

S9. Chilton, N. F.; Anderson, R. P.; Turner, L. D.; Soncini, A.; Murray, K. S. PHI: A powerful new program for the analysis of anisotropic monomeric and exchange-coupled polynuclear d- and f-block complexes. *J. Comput. Chem.* **2013**, *34*, 1164–1175. DOI: 10.1002/jcc.23234.

S10. Perdew, J. P.; Tao, J.; Staroverov, V. N.; Scuseria, G. E. Meta-generalized gradient approximation: Explanation of a realistic nonempirical density functional. *J. Chem. Phys.* **2004**, *120*, 6898–6911. DOI: 10.1063/1.1665298.

S11. Salomon, O.; Reiher, M.; Hess, B. A. Assertion and validation of the performance of the B3LYP* functional for the first transition metal row and the G2 test set. *J. Chem. Phys.* **2002**, *117*, 4729–4737. DOI: 10.1063/1.1493179.

S12. Becke, A. D. Density functional thermochemistry. III. The role of exact exchange. *J. Chem. Phys.* **1993**, *98*, 5648–5652. DOI: 10.1063/1.464913.

S13. Vitek, M.; Deng, J.-R.; Anderson, H. L.; Rončević, I. Global Aromatic Ring Currents in Neutral Porphyrin Nanobelts. *ACS Nano* **2025**, *19*, 1, 1405–1411. DOI: 10.1021/acsnano.4c14100.

S14. Perdew, J. P.; Ernzerhof, M.; Burke, K. Rationale for mixing exact exchange with density functional approximations. *J. Chem. Phys.* **1996**, *105*, 9982–9985. DOI: 10.1063/1.472933.

S15. Zhao, Y.; Truhlar, D. G. The M06 suite of density functionals for main group thermochemistry, thermochemical kinetics, noncovalent interactions, excited states, and transition elements: two new functionals and systematic testing of four M06-class functionals and 12 other functionals. *Theor Chem Account* **2008**, *120*, 215–241. DOI: 10.1007/s00214-007-0310-x.

S16. Yamaguchi, K.; Takahara, Y.; Fueno, T. in: V.H. Smith (Ed.). Applied Quantum Chemistry. Reidel, Dordrecht (1986), pp 155–184.

S17. Neese, F.; Wennmohs, F.; Becker, U.; Riplinger, C. The ORCA quantum chemistry program package. *J. Chem. Phys.* **2020**, *152*, 224108. DOI: 10.1063/5.0004608.
